# Dicyano- and tetracyanopentacene: foundation of an intriguing new class of easy-to-synthesize organic semiconductors†
†Electronic supplementary information (ESI) available: Experimental details (synthesis and solubility, NMR spectra, cyclic voltammetry, UV-Vis absorption/stability tests, thermal analysis, X-ray diffraction, theoretical calculations, optical microscopy and scanning electron microscopy, transistor fabrication and electrical characterization). CCDC 1443834 and 1519904. For crystallographic data in CIF or other electronic format see DOI: 10.1039/c7tc00143f



**DOI:** 10.1039/c7tc00143f

**Published:** 2017-02-16

**Authors:** Florian Glöcklhofer, Andreas Petritz, Esther Karner, Michael J. Bojdys, Barbara Stadlober, Johannes Fröhlich, Miriam M. Unterlass

**Affiliations:** a Institute of Applied Synthetic Chemistry , TU Wien , Getreidemarkt 9/163 , 1060 Vienna , Austria . Email: florian.gloecklhofer@tuwien.ac.at; b Joanneum Research , MATERIALS-Institute for Surface Technologies and Photonics , Franz-Pichler Straße 30 , 8160 Weiz , Austria; c Charles University in Prague , Faculty of Science , Department of Organic Chemistry , Hlavova 8 , 128 43 Prague 2 , Czech Republic; d Institute of Materials Chemistry , TU Wien , Getreidemarkt 9/165 , 1060 Vienna , Austria; e Institute of Organic Chemistry and Biochemistry ASCR , v.v.i. , Flemingovo nám. 2 , 166 10 Prague 6 , Czech Republic

## Abstract

Cyanated pentacenes are very promising candidate materials for ambipolar and n-type transistors. However, only a few examples have been obtained to date – all requiring lengthy, multi-step processes. Herein, we present the first preparation of 5,7,12,14-tetracyanopentacene (TCP) and a facile, scaled-up preparation of 6,13-dicyanopentacene (DCP). Both compounds are prepared by a one-pot synthesis using cheap quinones as starting materials. Detailed crystallographic investigations evince that the bulk assemblies of both cyanated pentacenes are dominated by non-covalent interactions, resulting in a dense, stable, face-to-face packing and in an intriguing packing motif for TCP. Very low frontier molecular orbital energy levels and a reversible bleaching of TCP are revealed by cyclic voltammetry. Finally, both cyanated pentacenes are used in proof-of-concept organic thin-film transistors (OTFTs) operating under ambient conditions. This work highlights the potential of cyanation for larger acenes and presents a straightforward route to the rational design of this promising class of materials.

## Abstract 2

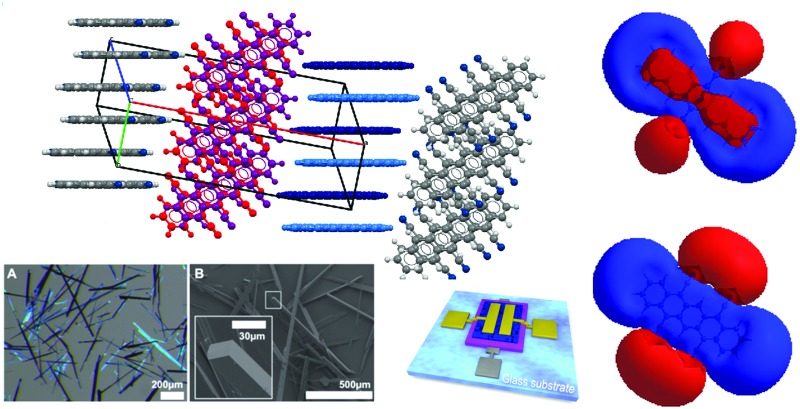
Extraordinarily low LUMO levels, dense molecular packing, an intriguing packing motif, reversible bleaching and OTFT operability under ambient conditions are revealed in a detailed investigation of multi-cyanated pentacenes.

## Introduction

Attaching electron-withdrawing groups (EWGs) to larger acenes is an important strategy to increase their stability. The highest occupied and lowest unoccupied molecular orbital (HOMO and LUMO) levels are lowered by EWGs, resulting in higher stability and making EWG incorporation a valuable strategy for the development of n-type and ambipolar organic semiconductors.^1,2^ A low LUMO level is particularly useful for facilitating electron injection at the electrodes and for stabilizing the resulting anionic species.^3^ High crystallinity, dense molecular face-to-face packing, and an edge-on orientation on the substrate promote overall transport of the injected charges.^1,4,5^ A dense molecular packing can also increase the device stability by preventing the penetration of water and oxygen into the organic material, thus forming a kinetic barrier.^6^

The cyano group is one of the functional groups with the largest electron-withdrawing inductive (-I) and mesomeric (-M) effects,^7,8^ while at the same time it is thermally very stable, enabling processing at elevated temperatures. Cyanation significantly lowers the LUMO level of an aromatic compound; the impact on the HOMO level is considered to be less pronounced due to the resulting extended π-electron system.^3^ Moreover, cyanated aromatic molecules crystallize in densely packed phases due to dipole interactions and hydrogen bonding.^4,9^ Small reorganization energies, another important parameter for efficient charge transport,^1^ were predicted computationally for cyanated pentacenes, in particular for 5,7,12,14-tetracyanopentacene (TCP).^10,11^ All this makes cyanation of larger acenes and, in particular, of pentacene the perfect strategy for the development of air-stable n-type and ambipolar organic semiconductors.

Although computationally-led design of molecular semi-conductors regularly features pentacene and its derivatives, a rational synthesis of cyanated pentacenes proves challenging. Hitherto, 6,13-dicyanopentacene (DCP) and its *N*,*N*′-dialkylated 2,3:9,10-bis(dicarboximide) are the only pentacenes reported that are cyanated at the inner rings.^12,13^ However, cyano groups close to or at the central ring are particularly useful, because degradation by oxidation or dimerization usually occurs at this position, if not prevented by functional groups.^14,15^ In our opinion, the complicated, multi-step synthesis is the reason for the lack of further cyanated pentacenes. Fortunately, our recently demonstrated new one-pot synthesis of cyanoarenes from quinones allows for expanding the promising group of cyanated acenes in a straightforward fashion.^16–18^ It is the aim of this work to develop a facile, scalable preparation protocol for cyanated pentacenes and to obtain a better understanding of the properties and non-covalent interactions (NCIs) of such compounds. Two different cyanated pentacenes, DCP and TCP, were intended for these investigations; the results facilitate the rational molecular design and preparation of further cyanated larger acenes.

## Experimental

### General procedure for the synthesis of DCP and TCP

*Taking into account the results of stability tests, light-shielding is recommended for the synthesis and work-up of TCP*. *n*-Butyllithium (DCP: 0.1 equiv./TCP: 0.2 equiv., 2.5 M in hexanes) was added carefully to rigorously stirred TMSCN (DCP: 2.2 equiv./TCP: 4.4 equiv.) in a reaction vial (equipped with a septum and an argon balloon for an inert gas atmosphere) at room temperature. *Safety note: n-butyllithium is a hazardous reagent, reacts violently with water, and should be handled with care*. After 15 min, the resulting suspension was transferred to stirred 6,13-pentacenequinone/5,7,12,14-pentacenetetrone (1.0 equiv.) in a 100 mL round bottom flask (again equipped with a septum and an argon balloon for an inert gas atmosphere), which was precooled to 0 °C with an ice bath. Dry dimethylformamide (DCP: 0.5 mL mmol^–1^ starting material/TCP: 1.0 mL mmol^–1^ starting material) was used to transfer the residues of the mixture and added immediately. The reaction stirred at 0 °C for 6 h. Dry CH_2_Cl_2_ (DCP: 3.0 mL mmol^–1^ starting material/TCP: 6.0 mL mmol^–1^ starting material) and PBr_3_ (DCP: 1.2 equiv./TCP: 2.4 equiv.) were then added slowly and the balloon was removed (to prevent contamination of the reaction by corrosion of the cannula). The reaction was placed in a covered ice-filled dewar flask (still stirred) and allowed to slowly warm up overnight (to about 15 °C). The dewar flask was removed and the reaction stirred at room temperature for another 4 h. Subsequently, the reaction was filtered (vacuum filtration) under a flow of nitrogen (or argon). The resulting dark blue solid was transferred into a round bottom flask using CH_2_Cl_2_. Silica was added, the solvent was evaporated *in vacuo* using a rotary evaporator and the resulting dry powder was used to load a small column for flash chromatography. CH_2_Cl_2_ was used as the eluent. The pure product fractions were combined and the solvent was evaporated *in vacuo* using a rotary evaporator. The solid residue was recrystallized twice from nitrobenzene and washed with dry EtOH after filtration. Yields: 25% (DCP)/12% (TCP).

#### Alternative work-up procedure

Direct purification by high-vacuum sublimation instead of flash chromatography has been tested. After the reaction has been filtered under a flow of nitrogen, the dark blue solid was washed with very small amounts of CH_2_Cl_2_ and dried *in vacuo*. The resulting powder was gradually heated at a pressure of 5 × 10^–6^ mbar or below, resulting in initial sublimation of the starting material and later sublimation of the cyanated product. Sublimation of the polar impurities has not been observed. Thus, purification by thermal gradient sublimation instead of flash chromatography is a feasible alternative.

## Results and discussion

### One-pot synthesis: scalable and low-cost

**Scheme 1:**
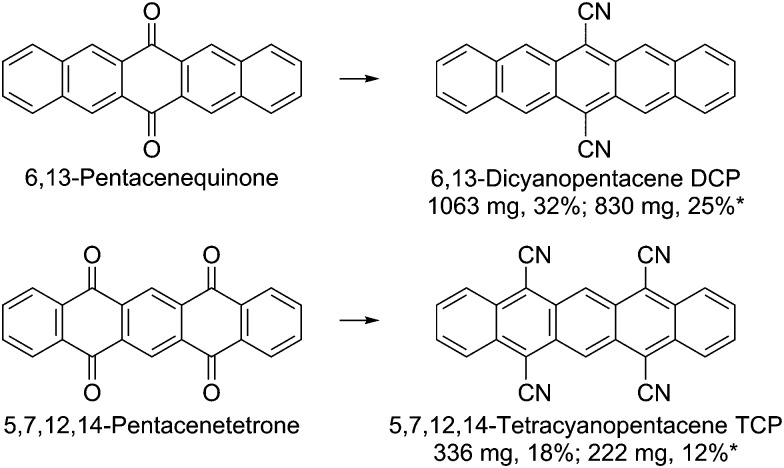
Synthesis of 6,13-dicyanopentacene (DCP) and 5,7,12,14-tetracyanopentacene (TCP). Reaction conditions: TMSCN, *n*BuLi, Ar, 15 min, rt; 6,13-pentacenequinone/5,7,12,14-pentacenetetrone, dimethylformamide, Ar, 6 h, 0 °C; CH_2_Cl_2_, PBr_3_, Ar, overnight, 0 °C to 15 °C; 4 h, rt. *Yields after recrystallizing twice from nitrobenzene.

Herein, we report a scaled-up one-pot synthesis of DCP and the first preparation of TCP (Scheme 1, see ESI† for a detailed step-by-step guide). The latter showcases the scope of the new protocol by the one-pot introduction of four cyano groups. The starting materials and the required reagents are commercially available at comparatively low cost and neither transition metal catalysts nor special reaction equipment are needed. The conversion proceeds at mild conditions *via* the intermediate formation of silylated cyanohydrins using trimethylsilyl cyanide (TMSCN); catalyzed by lithium cyanide, which can be easily prepared *in situ* by adding some drops of *n*-butyllithium to the TMSCN. Subsequent addition of PBr_3_ for the reductive aromatization yielded the cyanated pentacenes.

The reaction yields were not affected by the scale-up. Work-up was carried out by flash chromatography and subsequent recrystallization from nitrobenzene for higher purity. Since the solubility of the target compounds is low (DCP: 200 mg L^–1^, TCP: 60 mg L^–1^ in CH_2_Cl_2_), large amounts of solvent were required. Therefore, purification by sublimation instead of flash chromatography has been tested as an alternative. Based on the results, purification by thermal gradient sublimation is considered feasible. We want to emphasize that the optimized, facile synthetic protocol and the step-by-step guide are also aimed at non-chemists, minimizing the general issue of difficult access to newly published materials for physicists and device engineers. However, special care should be taken when handling *n*-butyllithium.

The stability of DCP and TCP in CH_2_Cl_2_ under air and exposure to white light was assessed by measuring UV-Vis absorption as a function of time (Fig. S8–S10, ESI†). For DCP, the UV-Vis absorption did not change within 5 h, revealing full stability. Despite the four EWGs, TCP is not fully stable under the same conditions; likely because there are no functional groups at the central ring. The absorbance decreased by 12% within 5 h.

### Cyclic voltammetry: revealing the lowest LUMO level of any pentacene derivative

**Fig. 1:**
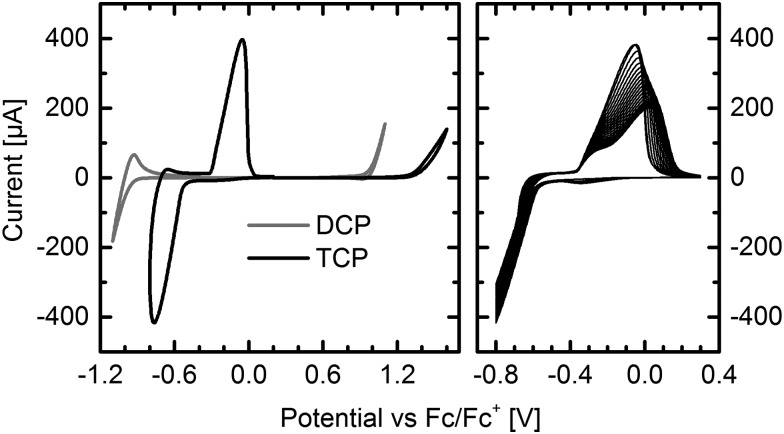
Cyclic voltammograms of DCP and TCP thin-films on ITO electrodes. Reduction and oxidation of DCP and TCP (left). Fifty reduction cycles of TCP (right). Supporting electrolyte: tetrabutylammonium tetrafluoroborate (*n*-Bu_4_NBF_4_) in acetonitrile (0.1 M). See Fig. S6 (ESI†) for measurements in solution.

Thin-films of DCP and TCP were vacuum deposited onto indium tin oxide (ITO) electrodes for cyclic voltammetry (CV) measurements. The results impressively demonstrate the impact of the two additional cyano groups and the different substitution positions (Fig. 1 left). In contrast to the reduction of DCP, which occurred at higher reduction potentials, TCP exhibits a very intense, reversible reduction and excellent electron injection. The delay of the reverse oxidation indicates a stabilization of the LUMO after injecting an electron. This stabilization of 0.23 eV may be attributed to a reversible dimerization of the radical anions, which has been reported for several smaller cyanated acenes.^19,20^ In contrast to our experiments, these measurements were carried out in solution, and a significantly larger stabilization of about 0.6 to 0.8 eV has been observed. The reversible bleaching of the otherwise deeply blue TCP thin-film, which was observed upon reduction (Fig. S5, ESI†), may be a result of the supposed dimerization. Running fifty measurement cycles (Fig. 1 right) resulted in a flattening of the reverse oxidation peak, which is attributed to partial detachment of the thin-film from the ITO electrode during the measurement. In contrast to the reduction, the oxidation was found to be irreversible for both compounds.

The frontier molecular orbital energy levels deduced from CV and absorption measurements are summarized in Table 1. Measuring thin-films, the HOMO level of DCP and the LUMO level determined by adding the optical bandgap are in excellent agreement with the previously reported values.^12^ The LUMO level deduced from CV measurements, which has not been previously determined, is about 0.3 eV higher. This is important, because a LUMO level of –4.0 to –4.1 eV or below is believed to enable air-stable electron transport.^1,2^ Depending on the method of determination, the LUMO values are either above or below this threshold.

**Table 1:** HOMO and LUMO levels of DCP and TCP compared to unsubstituted pentacene

	DCP thin-film	DCP solution	TCP thin-film	TCP solution	*Pentacene*
LUMO [eV]	–3.84^*a*^ –4.16^*b*^	–4.16^*a*^	–4.27^*a*^ –4.54^*b*^	–4.54^*a*^	*–2.70* ^*c*^
HOMO [eV]	–5.78^*a*^		–6.14^*a*^		*–4.88* ^*c*^

^*a*^Determined by CV from reduction and oxidation onsets on the premise that the Fc/Fc^+^ energy level is –4.80 eV.

^*b*^Determined by adding the optical bandgap energy (Fig. S7, ESI) to the HOMO level.

^*c*^From literature.^12^

The situation is less intricate for TCP. Both LUMO values deduced from thin-film measurements suggest that air-stable electron transport is possible. To the best of our knowledge, the LUMO level of –4.54 eV, determined by adding the optical bandgap to the HOMO level, is the lowest of any known pentacene derivative. Moreover, comparison with a large variety of other n-type semiconductors reveals that there are only very few organic semiconductors with a similarly low LUMO level.^1,2,6^ The LUMO level determined by CV is in a desired range for n-type organic semiconductors, between –4.1 and –4.4 eV.^2^ As expected, comparison to the unsubstituted pentacene shows that the LUMO is more strongly affected by the two cyano groups of DCP than the HOMO (Table 1). However, the two additional groups of TCP similarly affect both frontier molecular orbital energy levels.

CV measurements in solution (Fig. S6, ESI†) afforded exactly the same LUMO values for DCP and TCP as obtained from thin-films by adding the optical bandgap to the HOMO level.

### Crystal structures: dense, face-to-face packing and weak and strong H-bonds

A face-to-face lamellar molecular packing mode was described to be the most efficient for organic transistors; extensive work concentrated on tailoring molecules, in particular pentacenes, to achieve such a molecular arrangement.^1^ For tailoring further cyanated acenes, a detailed discussion of the crystallographic properties and an understanding of the non-covalent interactions (NCIs) of DCP and TCP are important.

**Fig. 2:**
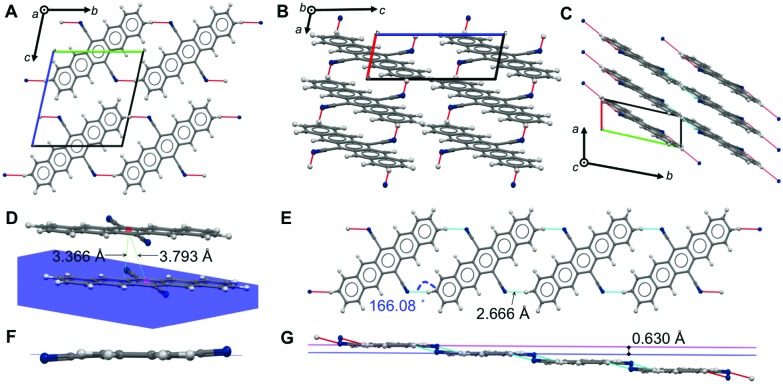
Crystal structure, packing and NCIs in DCP. View of DCP packing along *a* (A), *b* (B) and *c* (C); H-bonds are illustrated as red and blue dotted lines. (D) OFF π-stack between two DCP molecules, with a plane-plane separation of *d* = 3.366 Å, a centroid-to-centroid distance of *d* = 3.793 Å, and a parallel slip of 1.748 Å. (E and G) Adjacent DCPs H-bond into infinite, kinked ribbons with a kink height of 0.630 Å (G); *d*(N···H) = 2.666 Å, and ∢(N···H–C) = 166.08°. (F) H-Bonding forces CN groups out of coplanarity with the pentacene core.

We could recently report the crystal structure of DCP, which crystallizes in the triclinic *s*pace group *P*1 (no. 2), but the NCIs have not yet been discussed in detail.^17^ The molecular packing (Fig. 2) is most intriguing: DCP molecules form infinite π-stacks along *a* (Fig. 2C), with a plane separation distance of 3.366 Å, and a centroid-to-centroid distance of 3.793 Å (Fig. 2D). Hence, the DCPs are skewed in parallel by 1.748 Å, and the stacking corresponds to an offset face-to-face (OFF) stacking (also known as slipped/skewed stacking).^21^ Moreover, H-bonding in the molecular planes of DCP (4 H-bonds per DCP) results in infinite kinked ribbon structures (kink height is 0.630 Å) in the direction of *b* (Fig. 2E and G). The H-bonds are “weak” according to the Desiraju–Steiner classification (2.0 Å ≤ *d*(A···H–D) ≤ 3.0 Å),^22^ with *d*(N···H) = 2.666 Å, and ∢(N···H–C) = 166.08°, but have a pronounced effect on DCP's geometry: they force the cyano groups out of coplanarity with the pentacene core (Fig. 2F), *i.e.* the cyano group is slightly inclined (by 4.19(6)°).

For TCP, we can herein report (i) the single-crystal structure containing disordered solvent molecules after recrystallization from nitrobenzene (PhNO_2_) (in the following referred to as TCP·PhNO_2_) and (ii) the crystal data of TCP purified by sublimation, refined from powder XRD data.

**Fig. 3:**
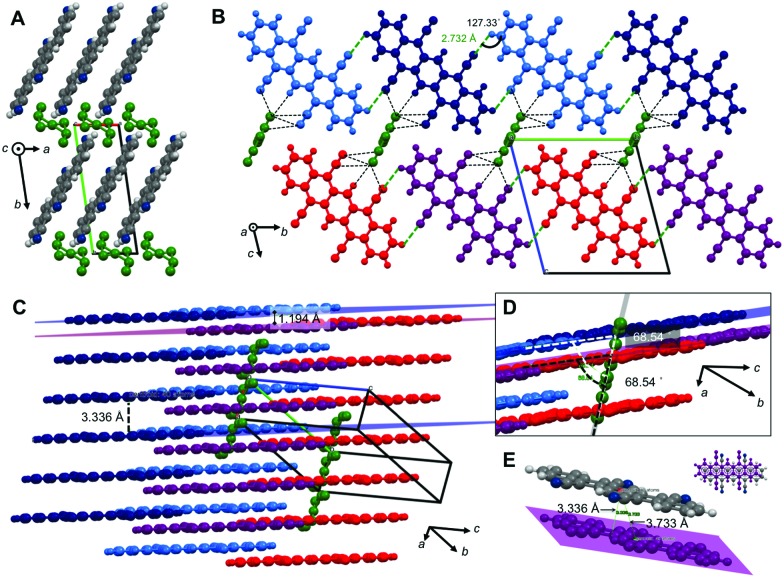
Crystal structure, packing and NCIs in TCP·PhNO_2_. Disordered PhNO_2_ molecules are shown in dark green. (A) Crystal structure of TCP·PhNO_2_ viewed along *c*. (B) View along *a*, TCP molecules π-stack into infinite columns along *a*; adjacent coplanar TCP molecules form H-bonded (H-bonds = green dotted lines) ribbons along *b*; disordered PhNO_2_ are in close-contact with TCP (*d* < sum of van der Waals radii; black dotted lines). (C) Side view of TCP columns; H-bonded TCPs are coplanar (*e.g.* violet and red; dark and light blue); coplanar TCPs are offset (parallel) by 1.194 Å with respect to neighboring molecular plane; (D) disordered PhNO_2_ form bands along *a*, in a 68.54° angle to TCP molecules. (E) OFF stack between to TCP molecules: *d*_plane–plane_ = 3.336 Å, *d*_centroid–centroid_ = 3.733 Å; the stack is parallel skewed by 1.675 Å.

TCP·PhNO_2_ crystallizes in the triclinic space group *P*1 (no. 2). The crystal structure of TCP·PhNO_2_ (disordered PhNO_2_ is highlighted in dark green) viewed along the *c*-axis (Fig. 3A) shows that the TCP molecules are π-stacked in columns of parallel TCP molecules along *a*. The plane separation distance between intra-column neighboring TCP molecules is 3.336 Å, which is shorter than the sum of van der Waals radii (Fig. 3C and E), and the centroid-to-centroid distance is 3.733 Å. Overall, the assembly corresponds to an OFF stacking, and parallel TCP molecules are displaced by 1.675 Å (Fig. 3E). In comparison, the OFF π-stacks in TCP·PhNO_2_ are very similar to those in DCP, with plane separation distances of approx. 3.4 Å, centroid-to-centroid distances of approx. 3.8 Å, and a displacement of two molecules by approx. 1.7 Å within a π-stack.

Columns in TCP·PhNO_2_ adjacent to each other in the longitudinal direction of the molecule (direction of the pentacene core, *i.e.* red and dark blue molecules in Fig. 3B and C) show an offset of 1.194 Å (Fig. 3C and D), whilst TCP molecules of adjacent columns perpendicular to the longitudinal direction (*i.e.* light and dark blue TCP molecules in Fig. 3B–D) form coplanar ribbons. The coplanarity is enabled by H-bonding between the cyano nitrogen atoms of one TCP molecule with an aromatic C–H of a neighboring TCP molecule (all of *d*_N···H_ = 2.732 Å, and ∢(N···H–C) =127.33°, Fig. 3B). These H-bonds also lie in the distance range of weak H-bonds.^22^

Overall, four H-bonds per TCP with another TCP result. Note that two of the four cyano groups do not participate in H-bonding with another TCP molecule, in favor of close-contacts with the incorporated disordered PhNO_2_. The disordered PhNO_2_ molecules are parallel to each other and form ribbons in a 68.54° angle with respect to the TCP molecule layers. Note that all TCP molecules in TCP·PhNO_2_ are parallel to each other.

The NCIs, *i.e.* both π-stacks and H-bonds are further illustrated by Hirshfeld surfaces and fingerprint plots (Fig. S16, ESI†).^23–25^

**Fig. 4:**
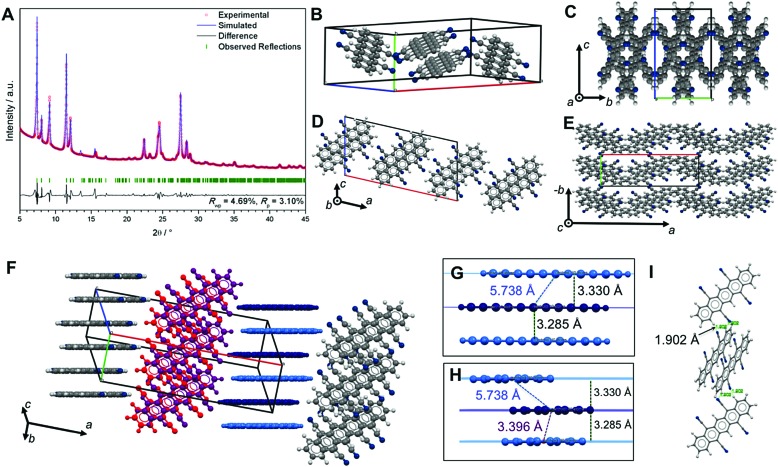
Crystal structure of sublimated TCP refined from PXRD. (A) Pawley fit performed on the PXRD pattern of TCP (*R*_wp_ = 4.69%, *R*_p_ = 3.10%) with the observed pattern in red, refined profile in blue, difference plot in black, and Bragg peak positions in green. (B) Unit cell of TCP. (C–E) Packing viewed along *a* (C), *b* (D) and *c* (E). (F–I) NCIs within the crystal structure: TCP molecules form OFF sandwich stacks that align in direction of *b* (F, dark and light blue TCPs) and *c* (F, red and violet TCPs). Detail view of π-stack viewed onto the TCP length axis (G), and onto the width axis (H): plane separation distances of two TCPs in a π-stack (*d* = 3.285 Å) and to neighboring TCP (*d* = 3.330 Å) are almost identical, however the centroid–centroid distance changes significantly (*d*_cent–cent,sandwich_ = 3.396 Å; *d*_cent–cent,neighbor_ = 5.738 Å). (I) TCP molecules of different stacks form symmetrical dimers by two H-bonds with each *d*(H···N) = 1.902 Å, and ∢(N···H–C_Ar_) = 147.16°.

For applications in organic electronics, where pentacenes are often deposited by sublimation processes, we were interested in obtaining the crystal structure of pure TCP (*i.e.* without incorporated solvent molecules). However, intense efforts to obtain solvent-free TCP single-crystals of sufficient size for single-crystal X-ray diffraction were not successful. We therefore performed powder X-ray diffraction (PXRD) of TCP obtained from high-vacuum sublimation, followed by a structure refinement. The PXRD pattern of TCP is shown in Fig. 4A. Pawley refinement of the pattern returns a monoclinic unit cell (*P*2/*a*, no. 13), but the pattern does not seem to be phase-pure with at least one additional phase contributing to the pattern, *e.g. Pc*. Monte Carlo/simulated annealing^26^ using the COMPASS optimized TCP molecule^27^ within the unit cell yields a conceivable structure.

However, *ab initio* structure solution and refinement using Rietveld methods does not return a satisfactory fit (Fig. S13, ESI†). This indicates that there is either more than one phase contributing to the PXRD pattern, or that some contaminant (*e.g.* solvent) co-crystallizes together with TCP.

The Pawley-refined structure bears OFF stacked TCP sandwiches (dark & light blue, and red & violet in Fig. 4F) that align into two different types of columns in which all TCP molecules are parallel. The angle between molecular planes within the two types of columns is 60.46° (*cf.* Fig. 4C and E). The plane–plane separation between π-stacked TCPs in a sandwich (3.285 Å) is almost identical with the plane–plane separation to the next neighbor (3.330 Å); however, centroid–centroid distances differ strongly (sandwich: 3.396 Å *vs.* neighbor: 5.738 Å, Fig. 4G and H). Moreover, the different TCP columns are connected with each other *via* H-bonds (two per TCP, see Fig. 4I), which lie in the range of strong H-bonds (*d*(N···H) = 1.902 Å and ∢(N···H–C) = 147.16°).^22^ While the action of aromatic compounds as H-bond donors is well known, such C_Ar_–H···A bonds are typically weak.^22,28^ The formation of strong C_Ar_–H···A bonds is highly unconventional and potentially rooted in the electron-poor aromatic system, which is expected to result in a strong positive polarization of the aromatic hydrogens.

**Fig. 5:**
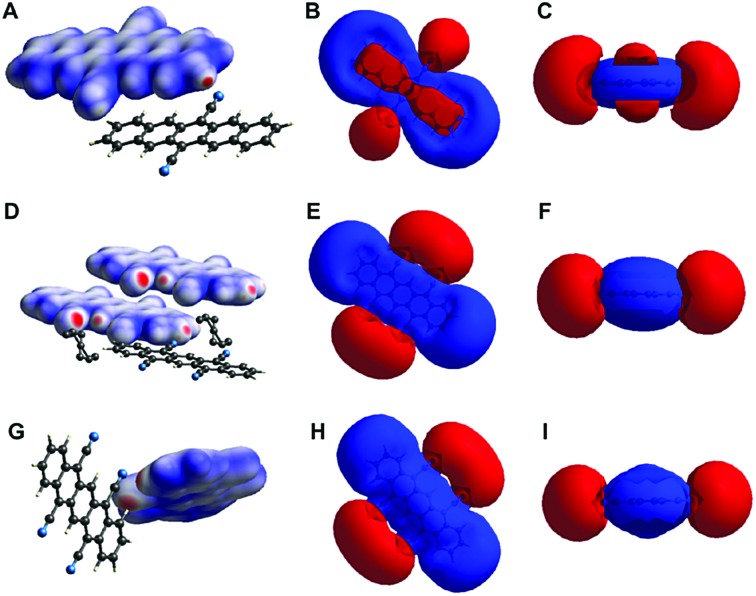
Electron density and electrostatic potential isosurfaces of DCP, TCP·PhNO_2_ and sublimated TCP. Electron density isosurfaces mapped with *d*_norm_ of DCP (A) and TCP in the crystal structure of TCP·PhNO_2_ (D) and of sublimated TCP (G). Electrostatic potential isosurfaces (red = negative; blue = positive; isovalue = 0.008) of cyanated pentacenes in the crystal structures of DCP (B and C), TCP·PhNO_2_ (E and F) and sublimated TCP (H and I) illustrate the strongly electron-withdrawing effect of cyano substituents on the pentacene core.

Overall, all the three crystal structures are dominated by NCIs: they show both (OFF) π-stacking and H-bonding. While H-bonding is enabled through the cyano group's ability to act as H-acceptor even of C_Ar_–H, π–π interactions are enabled by the pentacene core's planarity and fully aromatic nature. Given the importance of these interactions for the molecular packing (and consequently also for the electronic properties), we were interested in gathering further understanding of these NCIs in DCP and TCP. Therefore, we computed (i) the electron density isosurfaces mapped with *d*_norm_ (eqn (S1), ESI†) and (ii) the electrostatic potential isosurfaces of both compounds in the three crystal structures (Fig. 5) using CrystalExplorer.^29^ For the cyanated pentacenes in all three crystal structures, the electron density isosurfaces mapped with *d*_norm_ corroborate the existence of H-bonding (and close-contacts to disordered PhNO_2_). Red areas (indicating contacts shorter than van der Waals contacts) are found on nitrogen and hydrogen atoms participating in H-bonding/close-contacts in all three cases (Fig. 5A, D and G). The electrostatic potential isosurfaces underline the strong electron-withdrawing effect of the cyano substituents on the pentacene core: the four cyano groups of TCP are the most negative parts of the molecule (more negative than the π-electron cloud of the pentacene core) in both TCP·PhNO_2_ and sublimated TCP (Fig. 5E and H). Dipole interactions of the cyano groups and pentacene cores of neighboring sandwiches may explain the OFF stacking of the sandwiches in sublimated TCP.

### Crystal morphologies: bent needles after sublimation

**Fig. 6:**
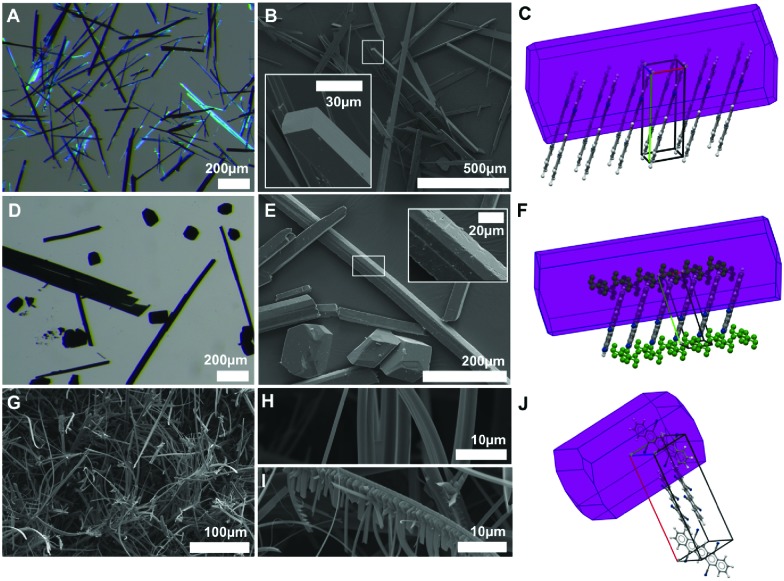
Crystal morphology of DCP, TCP·PhNO_2_ and sublimated TCP. Optical microscopy (A and D) and SEM images (B, E, G–I) of DCP (A and B), TCP·PhNO_2_ (D and E), and sublimated TCP (G–I) are in agreement with BFDH morphology predictions for DCP (needles, C) and TCP·PhNO_2_ (needles, F); and do not agree for sublimated TCP (obtained: bent needles, G–I; prediction: bulk crystallites, J).

DCP crystallizes in beautiful violet-blueish needles, as seen in optical and scanning electron microscopy (SEM) images (Fig. 6A and B). Morphology prediction for DCP based on the Bravais–Friedel–Donnay–Harker (BFDH) model corroborates needle shaped crystals, with the needles growing along the π-stacking direction (Fig. 6C). This comes as no surprise, since needles with the length axis coinciding with the stacking direction are often found for molecules that exhibit strong π–π interactions. TCP·PhNO_2_ is obtained as a mixture of needles and polygonal crystallites of approx. 100 μm diameter (Fig. 6D and E). For single crystal X-ray diffraction, a TCP·PhNO_2_ needle was selected; and the BFDH model also predicts needle morphology (Fig. 6F). Note that SEM images of TCP·PhNO_2_ needles at higher magnification evince small cracks (*cf.* close-up in Fig. 6E). These are most likely a consequence of the solvate PhNO_2_ evaporating with time. Sublimated TCP is obtained as mats of fibers of approx. 3 μm in diameter and several hundred μm in length (Fig. 6G). Interestingly, many of the fibers are bent (*cf.* close-up Fig. 6H) and a small number exhibits dendritic branching (*cf.* close-up in Fig. 6I). However, BFDH morphology prediction points at cuboid crystallites rather than fibrous structures.

### Vacuum deposition on dielectrics: investigating thin-films by scanning electron microscopy and X-ray diffraction

Further SEM investigations focused on the vacuum deposition behavior of DCP and TCP on different dielectrics, anticipating applications in organic thin-film transistors (OTFTs). SEM investigations are helpful to get a better knowledge of the vacuum deposition properties including the crystallite size. In addition, SEM studies can help to determine the orientation of the molecules with respect to the substrate surface and the source/drain electrodes. The orientation is critical for the electronic performance of OTFTs, because the electric current usually flows parallel to the substrate in these devices. Thus, a perpendicular (edge-on) orientation is beneficial. Note that the orientation of small molecules in thin-films usually depends on the substrate surface properties (surface energy, roughness) and on the deposition conditions.^30^ Consequently, the choice of the gate dielectric is essential to achieve good transistor performance.

Thin-films (50 nm) of DCP and TCP were prepared by thermal evaporation and deposition onto different dielectrics using a high vacuum system. The high thermal stability of DCP (>340 °C) and TCP (>400 °C) (Fig. S11 and S12, ESI†) facilitates this process.

**Fig. 7:**
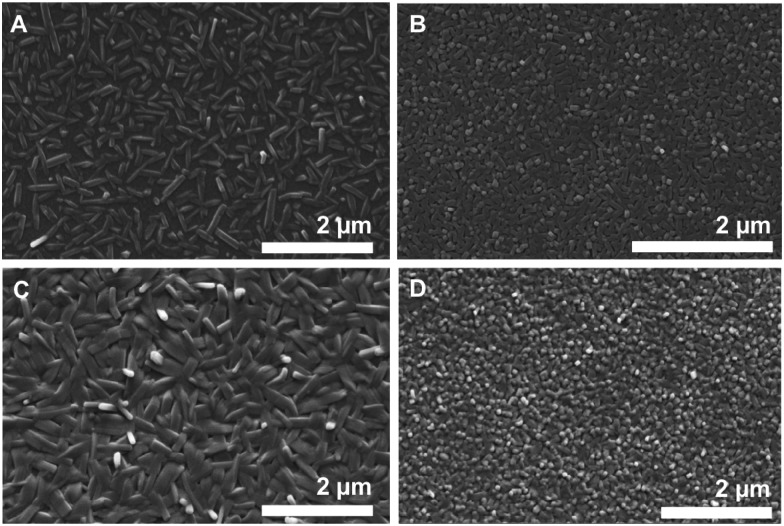
Vacuum deposited DCP and TCP on different gate dielectrics. SEM images of DCP (A and C) and TCP (B and D) layers on Al_2_O_3_ (A and B), Al_2_O_3_ + PNDPE (C and D).

For all dielectrics (Fig. 7 and Fig. S17–S48, ESI†), we find that vapor-deposited DCP crystallizes in needles in the colloidal size-range (about 0.5–1 μm in length and 100 nm in diameter). The size of DCP needles follows a rather narrow size-distribution, and the needles’ length axis orients in parallel to the substrate. The needle morphology is in agreement with the optical microscopy and SEM analysis of solution grown DCP, as well as BFDH morphology prediction (*cf.* Fig. 6A–C). Therefrom, we conclude that the DCP molecules, with their π-stacking along the needles’ principle axis, are oriented perpendicularly rather than parallel to the surface.

For TCP, we find much smaller crystallites: the majority phase on the different substrates are cuboid nanocrystallites of approx. 100 × 100 nm. Therefrom, one can conclude that (i) TCP is more difficult to crystallize than DCP, also from the vapor phase (tendency to smaller crystal sizes); and (ii) the morphology found for TCP on the dielectrics is in agreement with the BFDH morphology prediction of the Pawley-refined crystal structure of sublimated TCP. Moreover, the dielectric seems to have an impact on the TCP morphology and crystallite sizes: when Al_2_O_3_ is used (Fig. 7B), the TCP nanocrystallite morphology changes towards a needle-type one. This nicely illustrates the impact of the dielectric on the obtained morphology – and hence on the final transistor performance, as we discuss in the following. The observed variations in morphologies of TCP nano-assemblies make it difficult to draw up any conclusions regarding the orientation of the TCP molecules with respect to the substrate. However, the two different directions of the columns observed in the crystal structure of sublimated TCP increase the probability of a stacking in the desired direction.

The vapor-deposited thin-films were additionally studied by XRD measurements in grazing incidence geometry (Fig. S14 and S15, ESI†). Both DCP and TCP can be clearly identified by the presence of their major reflections. The observed reflections are broad and of low intensity, which is a consequence of the film thickness in the nano-range and the corresponding small crystallite sizes. Nevertheless, it is evident that vapor-deposited DCP crystallizes on the dielectrics in the same structure as DCP single crystals and vapor-deposited TCP in the same structure as sublimated TCP.

### Organic thin-film transistors: operable under ambient conditions

Preliminary organic thin-film transistor (OTFT) tests were carried out to investigate the applicability of DCP and TCP for devices operated under ambient conditions. The OTFTs were fabricated in a bottom gate, top contact device architecture (see ESI† for further details). Twice recrystallized DCP and TCP were used for the transistor fabrication without further purification. The best results were obtained using the high surface energy gate dielectrics Al_2_O_3_ (28 nm) and Al_2_O_3_ modified with an additional organic PNDPE (poly((±)*endo*,*exo*-bicyclo[2.2.1]hept-5-ene-2,3-dicarboxylic acid, diphenylester)) layer (40 nm) (see ESI†).

**Fig. 8:**
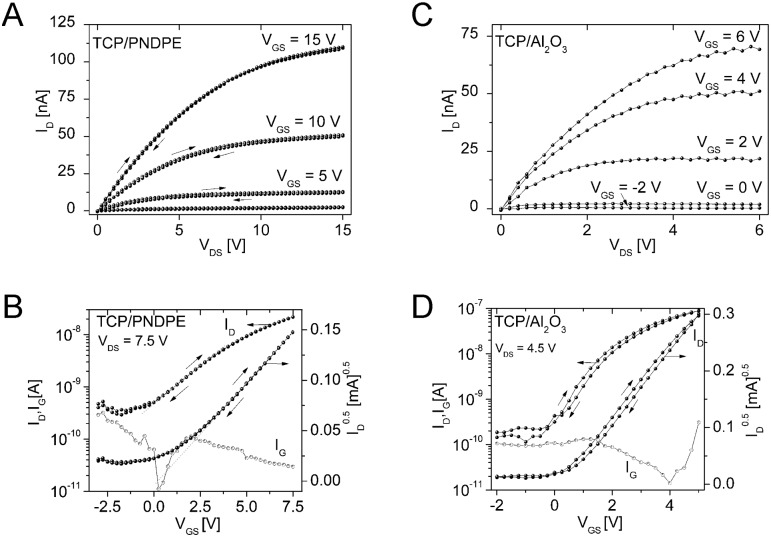
Output- (A and C) and transfer-characteristics (B and D) for TCP based OTFTs with (A and B) and without (C and D) an additional PNDPE capping layer on the Al_2_O_3_ gate dielectric. The gate leakage current characteristics *I*_G_(*V*_GS_) are also displayed (B and D).

Analysis of the electric characteristics of DCP based transistors revealed a clear ambipolar charge transport behavior for positive and negative source–drain voltages. Ambipolar behavior was also described when DCP was first reported by Kasuta *et al.* ^12^ The electrical characteristics of TCP based OTFTs were determined using the same dielectrics as for DCP, revealing n-type operation (Fig. 8). The output characteristics with a layer of PNDPE show a quadratic increase of the saturation drain current level with the gate bias and no hysteresis between the forward and reverse sweep (Fig. 8A).

For both materials, the electrical behavior is similar under inert and ambient measuring conditions (Table S3, ESI†), showing the potential of the tested materials and, generally, of larger cyanated acenes. The mobilities were low, but optimization of the processing (purification by thermal gradient sublimation, improved deposition by identifying the optimum deposition parameters, *i.e.* vacuum deposition rate and substrate temperature) is likely to improve the transistor performance significantly.

## Conclusions

The presented work provides a strong foundation for the further development of the material class of (multi-)cyanated, larger acenes. Several conclusions can be drawn:

(i) A cheap and scalable one-pot preparation of cyanated larger acenes from quinones is now easily possible and was demonstrated by the preparation of DCP and TCP. This is intriguing, since low synthesis costs are crucial for applications in organic electronics and elsewhere. Even the one-pot introduction of four cyano groups is possible following the new protocol.

(ii) Cyanation results in very low frontier molecular orbital energy levels (the lowest of any known pentacene derivative) and enables to obtain acenes that are suitable for n-type and ambipolar transistors operated under ambient conditions. This was demonstrated for DCP and TCP by preliminary transistor tests. It is expected that the transistor performance can be significantly improved by an optimization of the processing parameters. We are considering an application of the air-stable materials for (bio)sensors as particularly interesting.

(iii) The electrochemical reduction of TCP thin-films on ITO electrodes is highly reversible and goes along with a reversible bleaching and supposed dimerization. These features are interesting for applications such as data storage or for organic cathodes in rechargeable batteries. For the latter, bands of disordered solvent molecules like in TCP·PhNO_2_ may provide channels for counter ions, if such a structure can be obtained selectively.

(iv) High surface energy dielectrics are best suited for the vacuum deposition of DCP and TCP under the tested conditions, as shown by SEM investigations of thin-films deposited on different dielectrics. The processing of the materials is facilitated by the high thermal stability of the cyanated pentacenes.

(v) Weak and strong H-bonds, dipole interactions, and strong π–π interactions are common features of larger cyanated acenes as observed by investigating the crystal structures of DCP and TCP. Since these NCIs have a strong impact on the molecular packing, the obtained knowledge significantly facilitates a rational molecular design of further cyanated larger acenes.
