# Supplementary material for: Dicyano- and tetracyanopentacene: foundation of an intriguing new class of easy-to-synthesize organic semiconductors
Source: J Mater Chem C Mater. 2017 Feb 16;5(10):2603–10. doi: 10.1039/c7tc00143f (PMC5358504; doi:10.1039/c7tc00143f)
Supplement: Supplementary file 1 [file TC-005-C7TC00143F-s001.pdf]

Electronic Supplementary Material (ESI) for Journal of Materials Chemistry C.
This journal is © The Royal Society of Chemistry 2017

# Electronic supplementary information (ESI)

## Dicyano- and Tetracyanopentacene: Foundation of an Intriguing New Class of Easy-to-Synthesize Organic Semiconductors

Florian Glöcklhofer, Andreas Petritz, Esther Karner, Michael J. Bojdys, Barbara Stadlober, Johannes Fröhlich, and Miriam M. Unterlass

**Table of contents:**

1. Synthesis and solubility -S2-
2. $^{1}$H-, $^{13}$C-NMR spectra -S7-
3. Cyclic voltammetry -S9-
4. UV-Vis absorption / stability tests -S11-
5. Thermal analysis -S14-
6. X-ray diffraction -S15-
7. Theoretical calculations -S19-
8. Optical microscopy and scanning electron microscopy -S21-
9. Transistor fabrication and electrical characterization -S50-
10. References -S55-

# 1. Synthesis and solubility

Reagents and solvents were purchased from commercial suppliers and used without further purification, except for $PBr_3$, which was purified by distillation prior to use. Reactions were monitored by TLC (Merck, silica gel 60 $F_{254}$). Flash column chromatography was performed using silica 60 (Merck, 40-63 µm).

**1.1 6,13-Dicyanopentacene DCP**

*n*-Butyllithium (0.40 ml, 1.0 mmol, 0.1 eq, 2.5 M in hexanes) was added carefully to rigorously stirred TMSCN (2.18 g, 2.75 ml, 22.0 mmol, 2.20 eq) in a reaction vial (equipped with a septum and an argon balloon for an inert gas atmosphere) at room temperature. *Safety note: n-butyllithium is a hazardous reagent, reacts violently with water, and should be handled with care.* After 15 min, the resulting suspension was transferred to stirred 6,13-pentacenequinone (3.08 g, 10.0 mmol, 1.0 eq) in a 100 ml round bottom flask (again equipped with a septum and an argon balloon for an inert gas atmosphere), which was precooled to 0°C with an ice bath. Dry dimethylformamide (5.0 ml) was used to transfer the residues of the mixture and added immediately. The reaction stirred at 0°C for 6 h. Dry $CH_2Cl_2$ (30.0 ml) and $PBr_3$ (3.25 g, 1.13 ml, 12.0 mmol, 1.20 eq) were then added slowly and the balloon was removed (to prevent contamination of the reaction by corrosion of the cannula). The reaction was placed in a covered ice-filled dewar flask (still stirred) and allowed to slowly warm up overnight (to about 15°C). The dewar flask was removed and the reaction stirred at room temperature for another 4 h. Subsequently, the reaction was filtered (vacuum filtration) under a flow of nitrogen (or argon). The resulting dark blue solid was transferred into a round bottom flask using 500 ml of $CH_2Cl_2$. 15 g silica were added, the solvent was evaporated in vacuo using a rotary evaporator and the resulting dry powder was used to load a small column (25 g of fresh silica) for flash chromatography. $CH_2Cl_2$ was used as the eluent. *Note: the flash chromatography does not result in a perfect separation of the product and the starting material, but efficiently removes polar impurities.* The pure product fractions were combined and the solvent was evaporated in vacuo using a rotary evaporator. *Note: the low solubility of the product results in a large number of product fractions, but the eluent can be recycled after evaporation.* The solid residue (1.06 g, 3.2 mmol, 32 %) was recrystallized twice from 100 ml nitrobenzene and washed with dry EtOH after

filtration. DCP (830 mg, 2.5 mmol, 25%) was obtained as dark blue needles. The $^{1}$H NMR data obtained in $CD_2Cl_2$ is in accordance with our previous reports;[S1] however, in contrast to our previous assumption, DCP is fully stable in $CDCl_3$, enabling $^{1}$H and $^{13}$C NMR spectroscopy in this solvent. $^{1}$H NMR (600 MHz, $CDCl_3$, δ): 9.16 (s, 4H), 8.12 – 8.09 (m, 4H), 7.60 – 7.57 (m, 4H) ppm; $^{13}$C NMR (150 MHz, $CDCl_3$, δ): 133.9, 129.7, 128.6, 128.3, 125.2, 117.2, 110.8 ppm. Found: C, 87.3; H, 3.6; N, 8.4. Calc. for $C_{24}H_{12}N_2$: C, 87.8; H, 3.7; N, 8.5%.

*Alternative work-up procedure*: Direct purification by high-vacuum sublimation instead of flash chromatography has been tested. After the reaction has been filtered under a flow of nitrogen, the dark blue solid was washed with very small amounts of $CH_2Cl_2$ and dried in vacuo. The resulting powder was gradually heated at a pressure of $5x10^{-6}$ mbar, resulting in sublimation of the starting material at about 220°C and of DCP at about 270°C. Sublimation of the polar impurities has not been observed. Thus, purification by thermal gradient sublimation instead of flash chromatography is considered a feasible alternative.

The solubility of DCP in $CH_2Cl_2$ at room temperature was determined. A sample of DCP was sonicated for 20 min, residual solids were filtered off and 50 ml of the resulting solution were evaporated in vacuo. 10 mg of solid DCP were obtained, corresponding to a solubility of approx. 200 mg $L^{-1}$ (0.61 mmol $L^{-1}$).

**1.2 5,7,12,14-Tetracyanopentacene TCP**

*Taking into account the results of stability tests (section 4.2.) light shielding is recommended for the synthesis and work-up of TCP.*

*n*-Butyllithium (0.40 ml, 1.0 mmol, 0.2 eq, 2.5 M in hexanes) was added carefully to rigorously stirred TMSCN (2.18 g, 2.75 ml, 22.0 mmol, 4.40 eq) in a reaction vial (equipped with a septum and an argon balloon for an inert gas atmosphere) at room temperature. *Safety note: n-butyllithium is a hazardous reagent, reacts violently with water, and should be handled with care.* After 15 min, the resulting suspension was transferred to stirred 5,7,12,14-pentacenetetrone (1.69 g, 5.0 mmol, 1.0 eq) in a 100 ml round bottom flask (again equipped with a septum and an argon balloon for an inert gas atmosphere), which was precooled to 0°C with an ice bath. Dry dimethylformamide (5.0 ml) was used to transfer the residues of the mixture and

added immediately. The reaction stirred at 0°C for 6 h. Dry $CH_2Cl_2$ (30.0 ml) and $PBr_3$ (3.25 g, 1.13 ml, 12.0 mmol, 2.40 eq) were then added slowly and the balloon was removed (to prevent contamination of the reaction by corrosion of the cannula). The reaction was placed in a covered ice-filled dewar flask (still stirred) and allowed to slowly warm up overnight (to about 15°C). The dewar flask was removed and the reaction stirred at room temperature for another 4 h. Subsequently, the reaction was filtered (vacuum filtration) under a flow of nitrogen (or argon). The resulting dark blue solid was transferred into a round bottom flask using 500 ml of $CH_2Cl_2$. 8 g silica were added, the solvent was evaporated in vacuo using a rotary evaporator and the resulting dry powder was used to load a small column (8 g of fresh silica) for flash chromatography. $CH_2Cl_2$ was used as the eluent. *Note: the flash chromatography does not result in a perfect separation of the product and the starting material, but efficiently removes polar impurities.* The pure product fractions were combined and the solvent was evaporated in vacuo using a rotary evaporator. *Note: the low solubility of the product results in a large number of product fractions, but the eluent can be recycled after evaporation.* The solid residue (336 mg, 0.9 mmol, 18%) was recrystallized twice from 30 ml nitrobenzene and washed with dry EtOH after filtration. TCP (222 mg, 0.6 mmol, 12%) was obtained as a dark crystalline solid. $^1$H NMR (600 MHz, $CDCl_3$, δ): 9.94 (s, 2H), 8.59 – 8.57 (m, 4H), 7.88 – 7.87 (m, 4H) ppm; $^{13}$C NMR (150 MHz, $CDCl_3$, 65500 scans): no signals due to the very low solubility of TCP. Found: C, 81.3; H, 2.5; N, 14.2 (prior to sublimation). C, 82.5; H, 2.6; N, 14.5 (after sublimation). Calc. for $C_{26}H_{10}N_4$: C, 82.5; H, 2.7; N, 14.8%. The molecular structure was confirmed by single-crystal X-ray diffraction.

*Alternative work-up procedure*: Direct purification by high-vacuum sublimation instead of flash chromatography has been tested. After the reaction has been filtered under a flow of nitrogen, the dark blue solid was washed with very small amounts of $CH_2Cl_2$ and dried in vacuo. The resulting powder was gradually heated at a pressure of $3x10^{-6}$ mbar, resulting in sublimation of the starting material at about 250°C and of TCP at about 290°C. Sublimation of the polar impurities has not been observed. Thus, purification by thermal gradient sublimation instead of flash chromatography is considered a feasible alternative.

The solubility of TCP in $CH_2Cl_2$ at room temperature was determined. A sample of TCP was sonicated for 20 min, residual solids were filtered off and 50 ml of the resulting solution were evaporated in vacuo. 3 mg of solid TCP were obtained, corresponding to a solubility of approx. 60 mg $L^{-1}$ (0.16 mmol $L^{-1}$).

### 1.3 Step-by-step guide

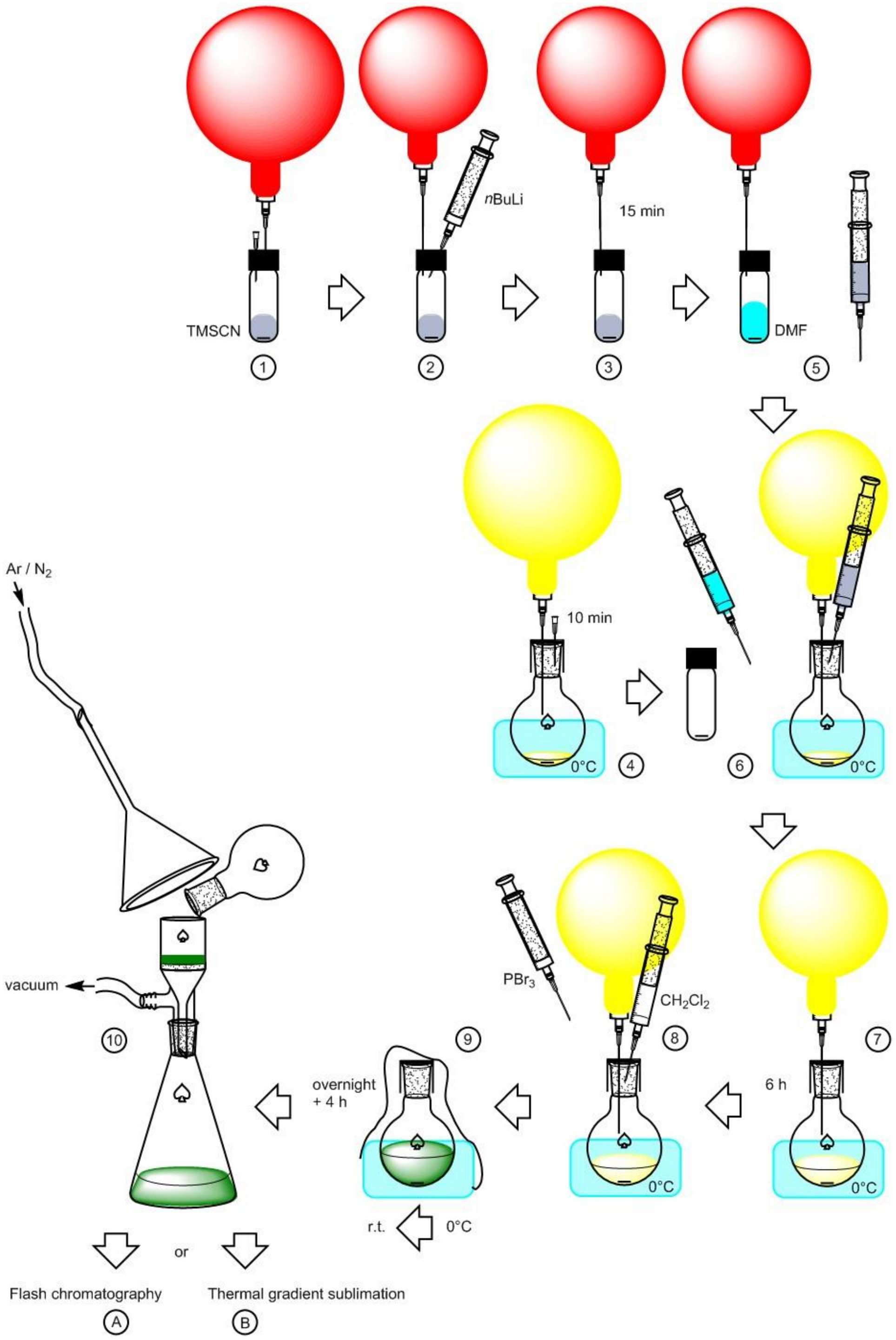


**Figure S1:** Illustration of the preparation of DCP and TCP.

① Provide TMSCN in a reaction vial equipped with a septum. Purge the vial briefly with argon to ensure an inert atmosphere.
② Carefully add n-butyllithium to the TMSCN under rigorous stirring at room temperature. A suspension is formed immediately. *Safety note: n-butyllithium is a hazardous reagent, reacts violently with water, and should be handled with care.*
③ Stir the suspension for 15 min.
④ Precool the quinone/tetrone starting material to 0°C in a 100 ml round bottom flask equipped with a septum using an ice bath. Purge the flask with argon for about 10 min while stirring the TMSCN suspension.
⑤ Pull the TMSCN suspension into a syringe. Add dry DMF to the reaction vial to dissolve residues.
⑥ Pull the DMF into a second syringe. Add the TMSCN suspension and the DMF to the precooled and stirred starting material one after another.
⑦ Stir for 6h at 0°C.
⑧ Slowly add dry $CH_2Cl_2$ and $PBr_3$ at 0°C. Remove the balloon to prevent contamination of the reaction by corrosion of the cannula.
⑨ Place the reaction in a covered ice filled dewar flask, but keep stirring. Allow the reaction to slowly warm up overnight (to about 15°C). Remove the dewar flask and stir for another 4 h at room temperature.
⑩ Filter off the solid by vacuum filtration under a flow of nitrogen.

Work-up Ⓐ:

Transfer the solid into a round bottom flask using $CH_2Cl_2$. Add silica and evaporate the solvent in vacuo using a rotary evaporator. Load a small column with the resulting dry powder. Use $CH_2Cl_2$ as the eluent for flash chromatography. Combine the pure product fractions and evaporate the solvent in vacuo again using a rotary evaporator. Recrystallize the solid residue form nitrobenzene. Filter off the resulting dark blue needles and finally wash them with dry EtOH. A second recrystallization can further improve the purity of the product.

Alternative work-up Ⓑ:

Dry the solid in vacuo at about 50°C and subject the solid to repeated thermal gradient sublimation. The product is obtained as a dark blue solid.

## 2. $^{1}$H-, $^{13}$C-NMR spectra

NMR spectra were recorded at 600 MHz for $^{1}$H and 150 MHz for $^{13}$C on a Bruker Avance III HD spectrometer. Data for $^{1}$H NMR is reported as follows: chemical shift in parts per million from TMS (tetramethylsilane), with the residual solvent signal as an internal reference ($CDCl_3$: δ = 7.26 ppm), multiplicity (s = singlet, m = multiplet) and integration. $^{13}$C NMR data is reported in ppm from TMS using the central peak of the solvent as reference ($CDCl_3$: δ = 77.16 ppm).

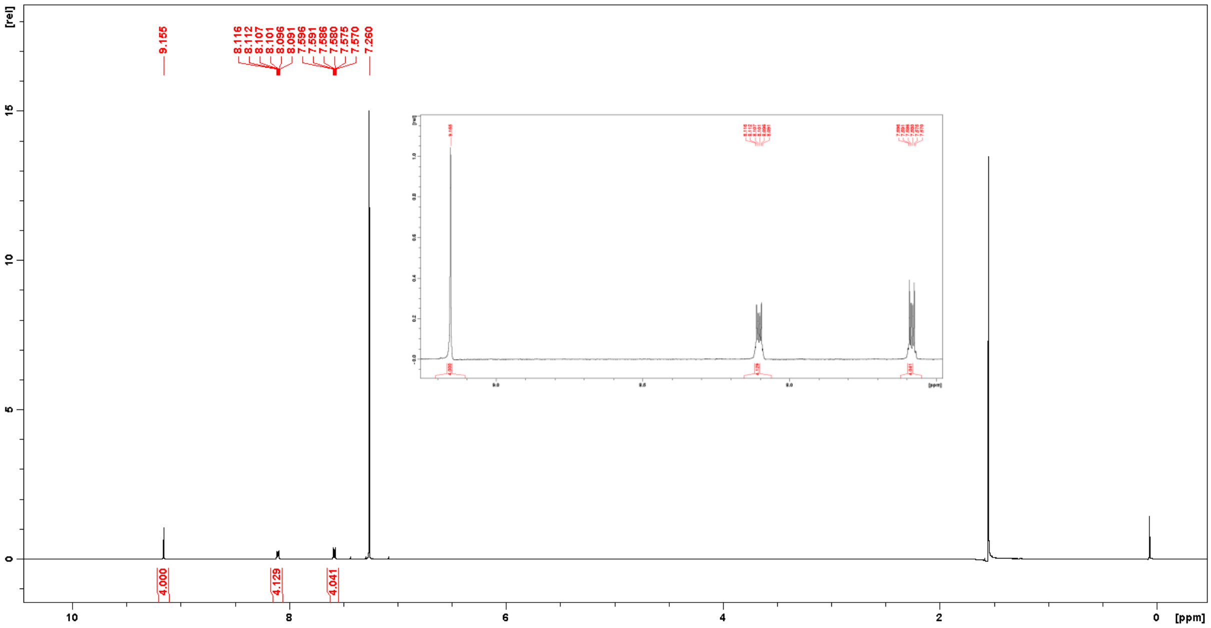


**Figure S2:** $^{1}$H NMR spectrum (600 MHz, $CDCl_3$) of DCP

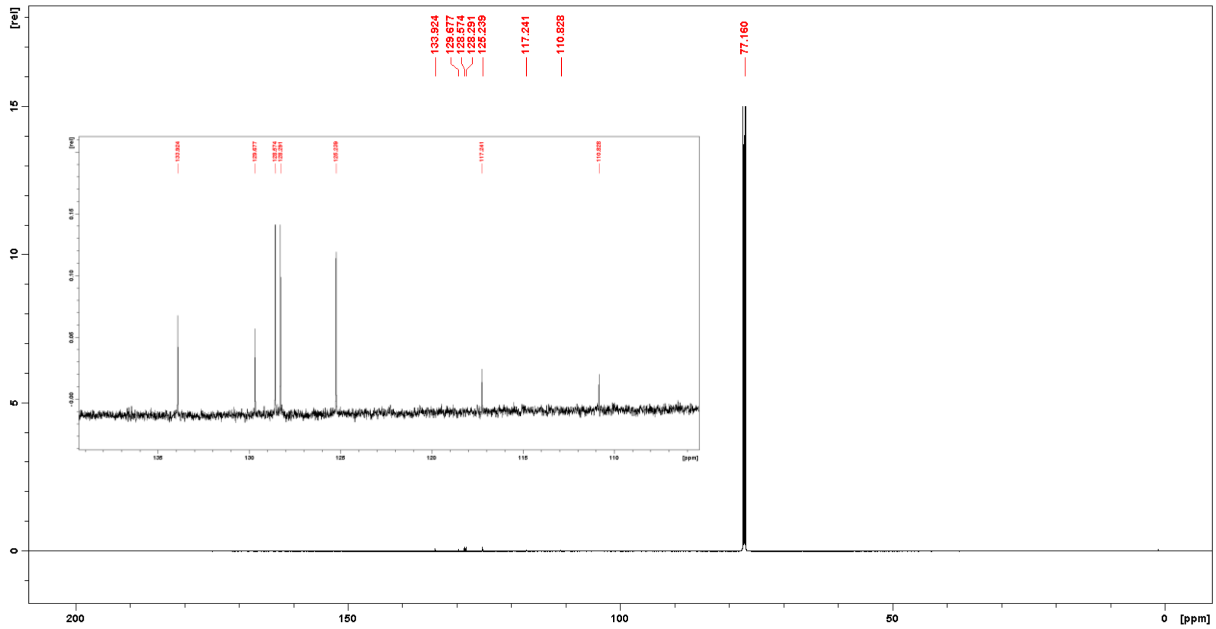


**Figure S3:** $^{13}$C NMR spectrum (150 MHz, $CDCl_3$) of DCP

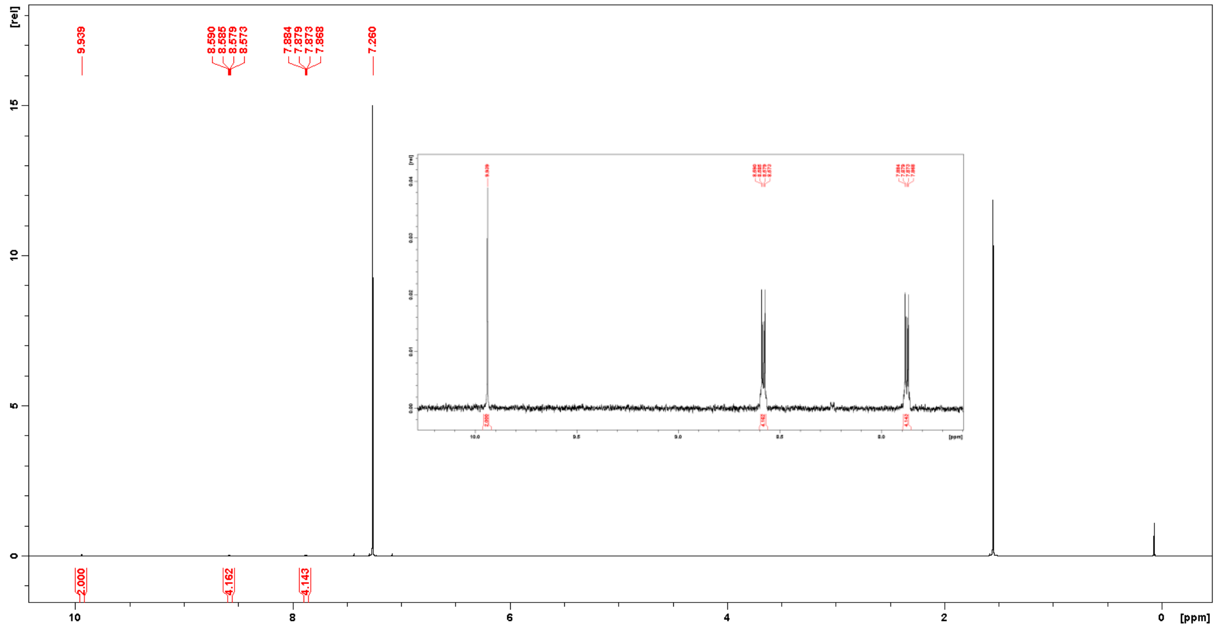


**Figure S4:** $^{1}$H NMR spectrum (600 MHz, $CDCl_3$) of TCP

## 3. Cyclic voltammetry

Cyclic voltammetry (CV) measurements of thin-films and solutions were carried out at room temperature using Metrohm Autolab B.V equipment (PGSTAT128N, differential electrometer amplifier). The measurements were performed at a scan rate of 0.05 $Vs^{-1}$. A three electrode configuration consisting of an ITO (thin-film measurements) or platinum (measurements in solution) working electrode, a platinum counter electrode, and a silver chloride coated silver reference electrode was used. Ferrocene (Fc) was measured as the reference material. HOMO and LUMO levels were deduced from the reduction and oxidation onset potentials of DCP and TCP on the premise that the $Fc/Fc^+$ energy level is -4.80 eV.

### 3.1 Thin-film measurements

The measurements were performed using vacuum deposited DCP and TCP thin-films on indium tin oxide (ITO) working electrodes. The thin-films were prepared under the same conditions as for the transistor fabrication. $n$-$Bu_4NBF_4$ in acetonitrile (0.1 M) was used as the supporting electrolyte. The supporting electrolyte solutions were purged with nitrogen for 10 minutes prior to the measurements.

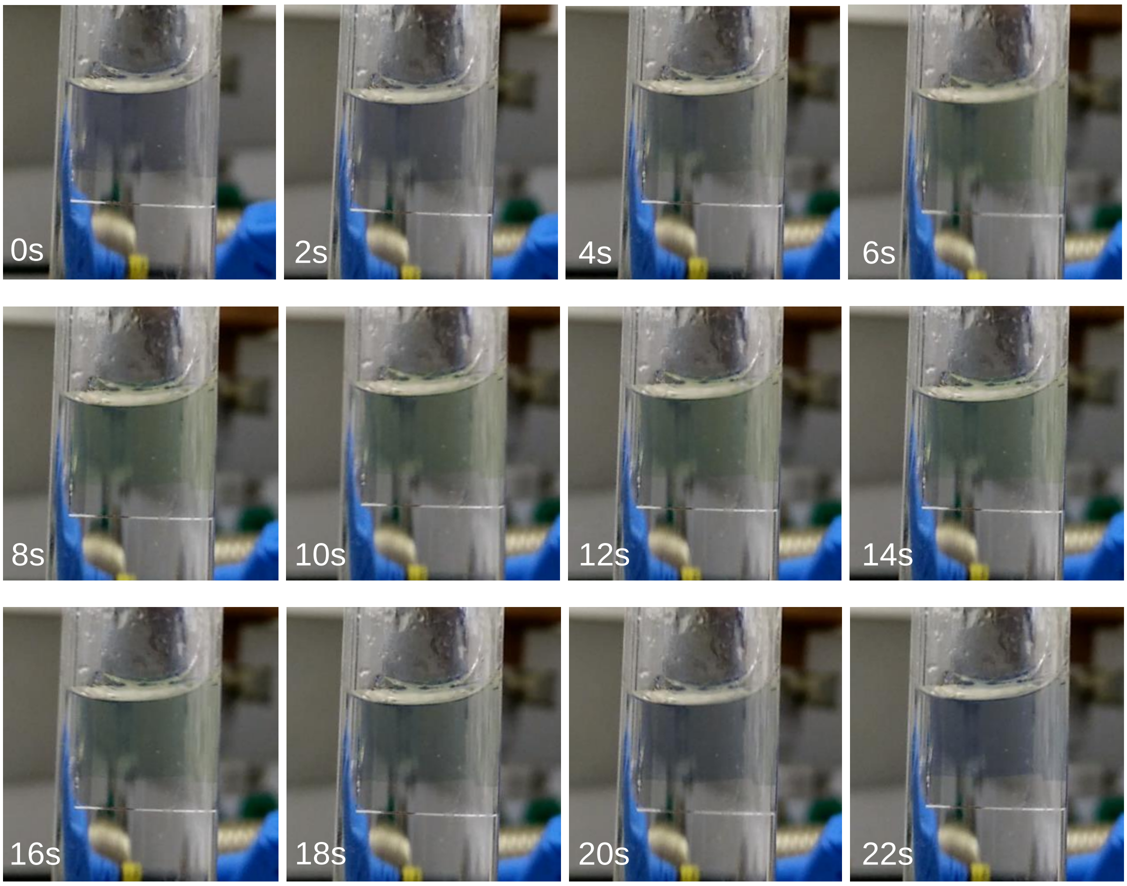


**Figure S5:** Reversible bleaching of the TCP thin-film upon reduction

### 3.2. Measurements in solution

The measurements were performed using saturated solutions of DCP and TCP in $CH_2Cl_2$. *n*-$Bu_4NBF_4$ (0.1 M) was used as the supporting electrolyte. The solutions were purged with nitrogen for 10 minutes prior to the measurements. The determined reduction onset potentials correspond to LUMO levels of -4.16 eV (DCP) and -4.54 eV (TCP), which are exactly the same values as obtained from thin-films by adding the optical bandgap to the HOMO level.

Due to the low concentration of the analytes, a reliable determination of the oxidation onset potentials was not possible.

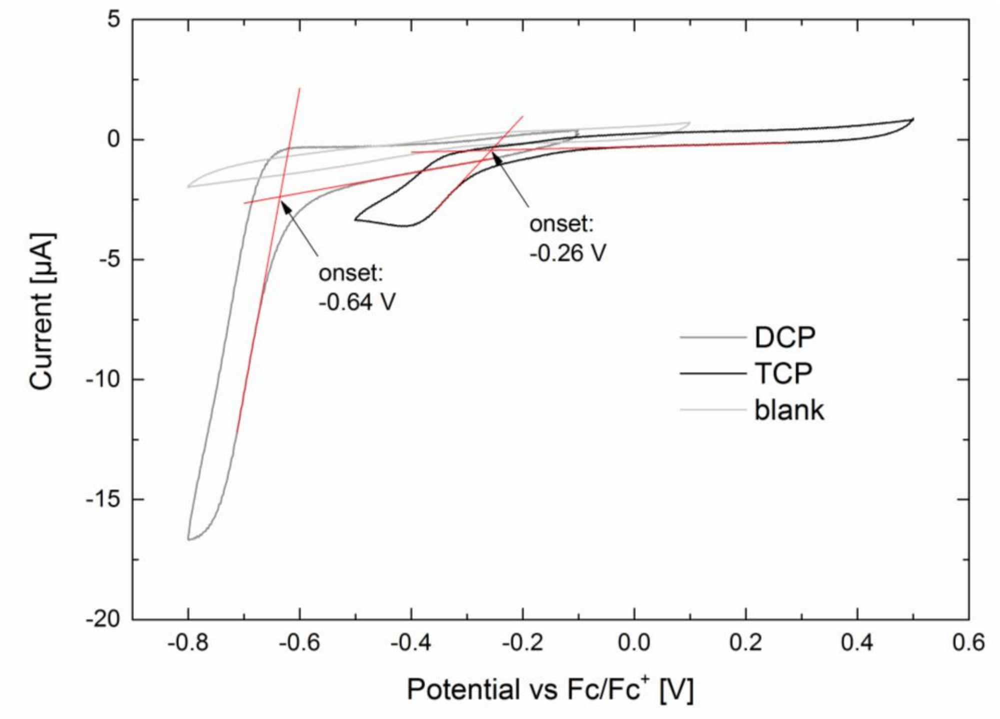


**Figure S6:** Cyclic voltammograms of DCP and TCP in solution.

## 4. UV-Vis absorption / stability tests

The absorption spectra were recorded at room temperature on a Perkin Elmer Lambda 750 spectrometer.

### 4.1. Thin-film absorption

Vacuum deposited films of DCP (100 nm film thickness) and TCP (85 nm film thickness) on glass slides were used to determine the thin-film absorption spectra. The determined absorption onsets correspond to optical bandgap energies of 1.62 eV for DCP (766 nm) and 1.60 eV for TCP (774 nm).

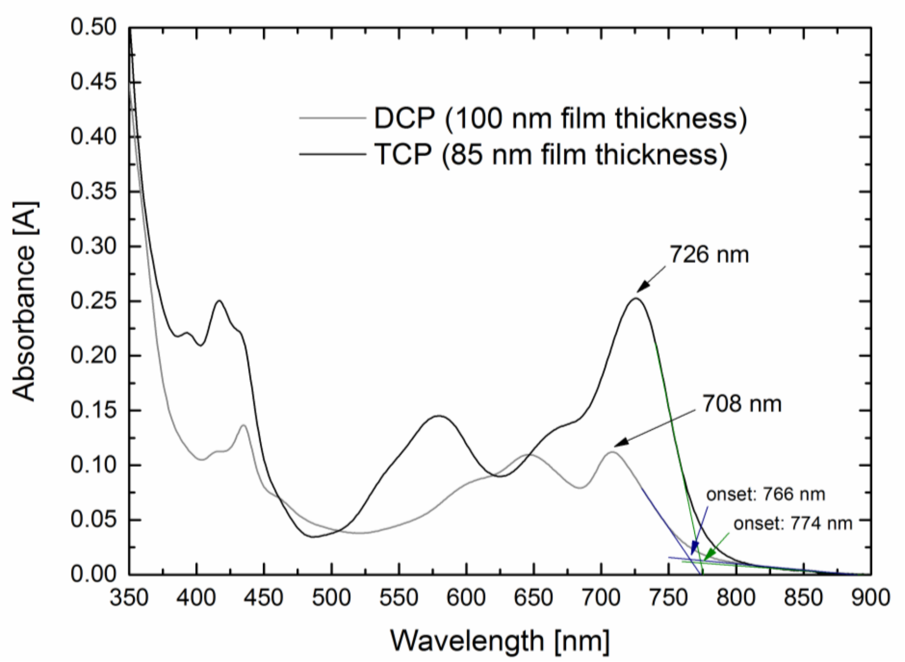


**Figure S7:** Thin-film absorption spectra of DCP and TCP

### 4.2. Stability tests in solution

Solutions of DCP and TCP in $CH_2Cl_2$ (approx. 0.05 mM) were measured in quartz glass cuvettes. The solutions (25 ml) were stored under air in 50 ml round-bottom flasks and exposed to the light of two 36 W neon tubes in a distance of 1 m. Glass stoppers were used to avoid evaporation of the solvent.

Samples were taken every hour to record absorption spectra (Figure S8 and S9). The relative absorbance ($A/A_0$) at the absorption maximum (DCP: 650 nm, TCP: 638 nm) is plotted in Figure S10.

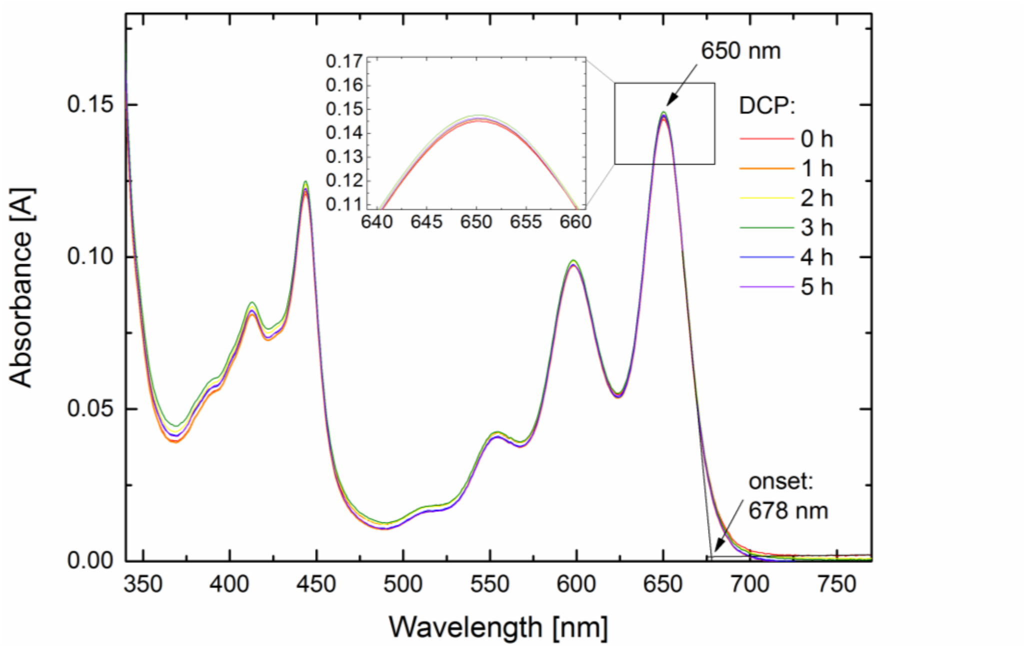


**Figure S8:** Absorption spectra of DCP in $CH_2Cl_2$

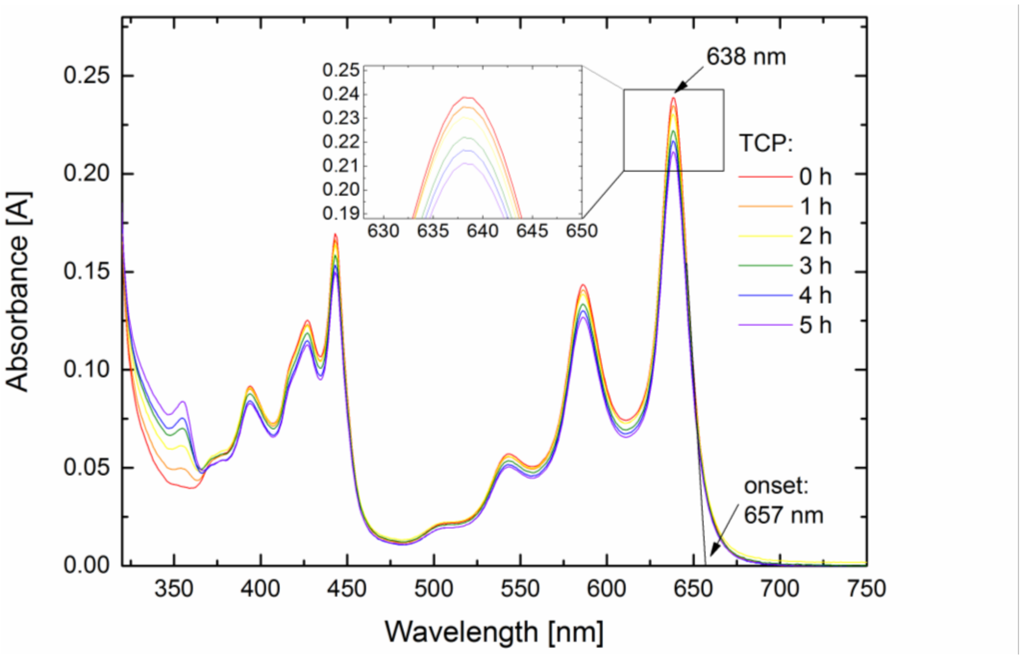


**Figure S9:** Absorption spectra of TCP in $CH_2Cl_2$

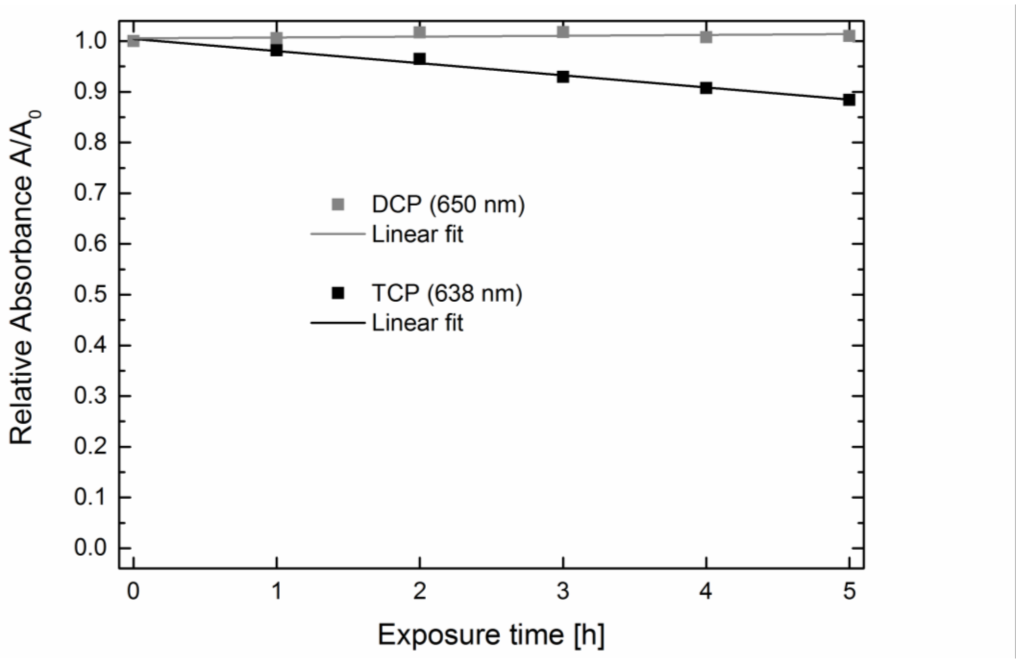


**Figure S10:** Relative absorbance of DCP and TCP at the absorption maximum as a function of time

# 5. Thermal analysis

Thermogravimetric analysis was carried out at a heating rate of 10 K min$^{-1}$ under helium atmosphere on a Netzsch STA 449F1, working with aluminum pans with pierced lids.

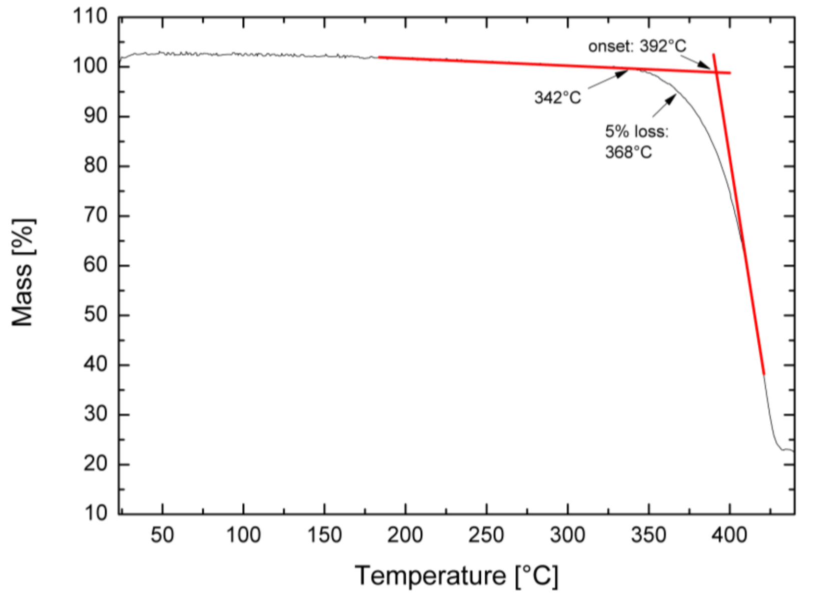


**Figure S11:** Thermogravimetric analysis of DCP

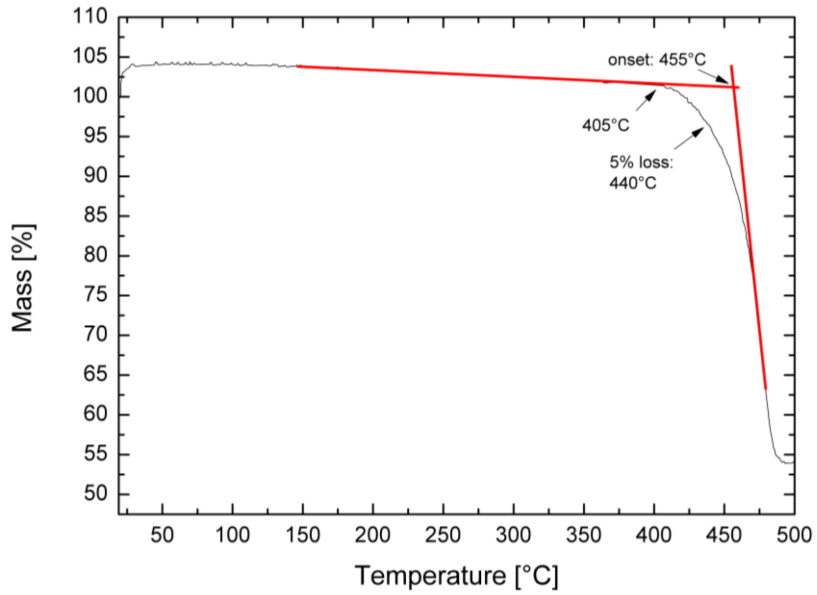


**Figure S12:** Thermogravimetric analysis of TCP

Differential scanning calorimetry did not evince glass transition or melting up to 500°C.

# 6. X-ray diffraction

## 6.1 Single-crystal X-ray diffraction (TCP·$PhNO_2$)

Crystals of a nitrobenzene solvate of TCP were obtained by recrystallization from boiling nitrobenzene. Diffraction data were collected at T = 100 K in a dry stream of nitrogen on a Bruker Kappa APEX II diffractometer system using graphite-monochromatized Mo-$K_α$ radiation (λ = 0.71073 Å) and fine-sliced φ- and ω-scans. Data were reduced to intensity values with SAINT and an absorption correction was applied with the multi-scan approach
implemented in SADABS.[S2] The structure was solved by charge flipping implemented in SUPERFLIP[S3] and refined against F with JANA2006.[S4] Non-hydrogen atoms were refined anisotropically. The H atoms were placed in calculated positions and thereafter refined as riding on the parent atoms.

CCDC 1519904 contains the supplementary crystallographic data for TCP·$PhNO_2$. The CCDC data can be obtained free of charge from The Cambridge Crystallographic Data Centre via www.ccdc.cam.ac.uk/data_request/cif.

Contributions of disordered nitrobenzene molecules were removed with the SQUEEZE routine of PLATON.[S5]

## 6.2 Powder X-ray diffraction (sublimated TCP)

High resolution powder X-ray diffraction (PXRD) data were collected on a PANalytical Empyrean equipped with a hybrid monochromator (2x Ge (220) for Cu) at 45 kV, 40 mA and a PIXcel 3D detector. $LaB_6$ was used as a standard for determination of the resolution of the diffractometer.

Structural refinement and Le Bail fitting was carried out using Materials Studio (v6.0.0) and FullProf software packages. The initial unit cell was determined by whole-pattern decomposition using the LeBail/Pawley methods.[S6] For the structural refinement of the P2/A model against the experimental diffraction data, geometric restraints were applied to all bond distances and angles in the TCP monomer that was geometry-optimized using COMPASS code.[S7] Conceivable orientations of the TCP monomer within the unit-cell were obtained using Monte Carlo/simulated annealing.[S8]

```
_chemical_name_systematic 'TCP'
_chemical_formula_sum ' C26 H10 N4 '

_cell_length_a                24.31(2)
_cell_length_b                7.65(5)
_cell_length_c                12.42(1)
_cell_angle_alpha             90.
_cell_angle_beta              102.(8)
_cell_angle_gamma             90.
_symmetry_space_group_name_H-M   'P2/A'
_symmetry_Int_Tables_number     '13'
_symmetry_cell_setting          monoclinic

loop_
_symmetry_equiv_pos_as_xyz
 x,y,z
 -x+1/2,y,-z
 -x,-y,-z
 x+1/2,-y,z

loop_
_atom_site_label
_atom_site_type_symbol
_atom_site_fract_x
_atom_site_fract_y
_atom_site_fract_z
_atom_site_U_iso_or_equiv
_atom_site_adp_type
_atom_site_occupancy
N1   N   0.03488  0.34798  0.09681  0.03015  Uani  1.00
C2   C  -0.17232  0.41050  0.02022  0.02442  Uani  1.00
H3   H  -0.18982  0.34698 -0.05943  0.02900  Uiso  1.00
N4   N   0.19963  0.51726  0.39161  0.03049  Uani  1.00
C5   C  -0.11469  0.41244  0.06195  0.02179  Uani  1.00
H6   H  -0.08797  0.34862  0.01251  0.02600  Uiso  1.00
C7   C  -0.09060  0.49329  0.16433  0.01913  Uani  1.00
C8   C  -0.03201  0.49601  0.20764  0.01829  Uani  1.00
C9   C  -0.00929  0.57701  0.30975  0.01702  Uani  1.00
C10  C   0.04887  0.58135  0.35526  0.01803  Uani  1.00
H11  H   0.07744  0.51897  0.30890  0.02200  Uiso  1.00
C12  C   0.07268  0.66094  0.45654  0.01688  Uani  1.00
C13  C   0.13137  0.66341  0.50017  0.01788  Uani  1.00
C14  C   0.15400  0.74376  0.60224  0.01832  Uani  1.00
C15  C  -0.18660  0.56906  0.17901  0.02110  Uani  1.00
H16  H  -0.21681  0.62919  0.22228  0.02500  Uiso  1.00
C17  C  -0.20837  0.48899  0.07893  0.02446  Uani  1.00
H18  H  -0.25421  0.48706  0.04544  0.02900  Uiso  1.00
C19  C   0.00522  0.41391  0.14609  0.02106  Uani  1.00
C20  C   0.16920  0.58209  0.43965  0.02153  Uani  1.00
N21  N  -0.00851  0.96985  0.73032  0.03015  Uani  1.00
C22  C   0.19856  0.90571  0.80733  0.02442  Uani  1.00
H23  H   0.21605  0.96865  0.88721  0.02900  Uiso  1.00
N24  N  -0.17322  0.80121  0.43550  0.03049  Uani  1.00
C25  C   0.14095  0.90457  0.76532  0.02179  Uani  1.00
H26  H   0.11420  0.96832  0.81472  0.02600  Uiso  1.00
C27  C   0.11689  0.82456  0.66267  0.01913  Uani  1.00
C28  C   0.05831  0.82245  0.61911  0.01829  Uani  1.00
C29  C   0.03558  0.74170  0.51691  0.01702  Uani  1.00
C30  C  -0.02259  0.73707  0.47151  0.01803  Uani  1.00
H31  H  -0.05114  0.79899  0.51807  0.02200  Uiso  1.00
C32  C  -0.04638  0.65780  0.37014  0.01688  Uani  1.00
C33  C  -0.10509  0.65520  0.32659  0.01788  Uani  1.00
C34  C  -0.12772  0.57411  0.22475  0.01832  Uani  1.00
C35  C   0.21285  0.74781  0.64830  0.02110  Uani  1.00
H36  H   0.24303  0.68748  0.60504  0.02500  Uiso  1.00
C37  C   0.23460  0.82708  0.74866  0.02446  Uani  1.00
H38  H   0.28044  0.82818  0.78244  0.02900  Uiso  1.00
C39  C   0.02108  0.90433  0.68074  0.02106  Uani  1.00
C40  C  -0.14290  0.73647  0.38719  0.02153  Uani  1.00
loop_
_atom_site_aniso_label
_atom_site_aniso_U_11
_atom_site_aniso_U_22
_atom_site_aniso_U_33
_atom_site_aniso_U_12
_atom_site_aniso_U_13
_atom_site_aniso_U_23
N1    0.02960  0.03309  0.02706  0.00750  0.00476  0.00879
C2    0.02330  0.02710  0.02199 -0.00028  0.00318  0.00008
N4    0.02880  0.02638  0.03388  0.00365  0.00176  0.00945
C5    0.01930  0.02400  0.02037 -0.00176  0.00075  0.00173
C7    0.01360  0.02064  0.02083 -0.00248 -0.00118  0.00217
C8    0.01390  0.01922  0.01955 -0.00244 -0.00100  0.00274
C9    0.01270  0.01702  0.01950 -0.00342 -0.00042  0.00088
C10   0.01480  0.01736  0.01937 -0.00211 -0.00171  0.00239
C12   0.01310  0.01532  0.02019 -0.00326 -0.00069  0.00113
C13   0.01330  0.01632  0.02185 -0.00263 -0.00079  0.00119
C14   0.01310  0.01764  0.02184 -0.00336 -0.00122 -0.00102
C15   0.01750  0.01746  0.02607 -0.00121 -0.00004 -0.00102
C17   0.01850  0.02407  0.03000  0.00196  0.00364 -0.00176
C19   0.01940  0.02375  0.01960 -0.00013  0.00341  0.00074
C20   0.01830  0.02103  0.02373 -0.00079  0.00137  0.00190
N21   0.02960  0.03309  0.02706  0.00750  0.00476  0.00879
C22   0.02330  0.02710  0.02199 -0.00028  0.00318  0.00008
N24   0.02880  0.02638  0.03388  0.00365  0.00176  0.00945
C25   0.01930  0.02400  0.02037 -0.00176  0.00075  0.00173
C27   0.01360  0.02064  0.02083 -0.00248 -0.00118  0.00217
C28   0.01390  0.01922  0.01955 -0.00244 -0.00100  0.00274
C29   0.01270  0.01702  0.01950 -0.00342 -0.00042  0.00088
C30   0.01480  0.01736  0.01937 -0.00211 -0.00171  0.00239
C32   0.01310  0.01532  0.02019 -0.00326 -0.00069  0.00113
C33   0.01330  0.01632  0.02185 -0.00263 -0.00079  0.00119
C34   0.01310  0.01764  0.02184 -0.00336 -0.00122 -0.00102
C35   0.01750  0.01746  0.02607 -0.00121 -0.00004 -0.00102
C37   0.01850  0.02407  0.03000  0.00196  0.00364 -0.00176
C39   0.01940  0.02375  0.01960 -0.00013  0.00341  0.00074
C40   0.01830  0.02103  0.02373 -0.00079  0.00137  0.00190
```

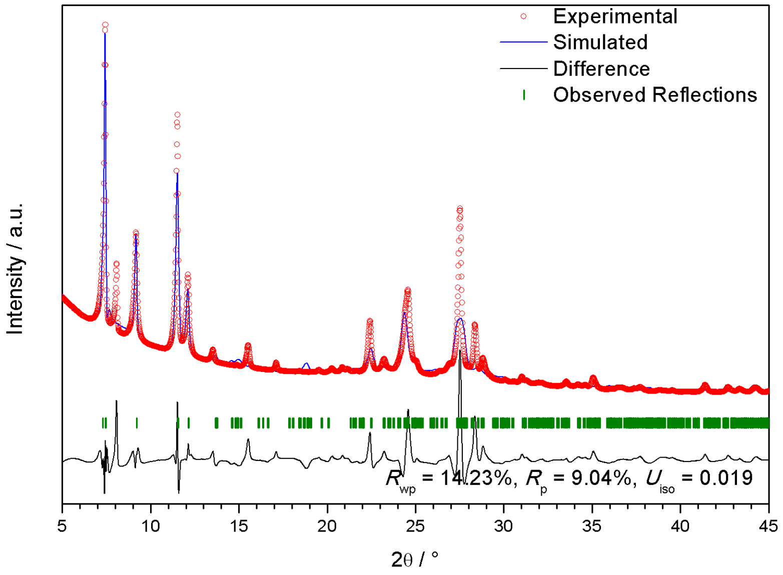


**Figure S13:** Rietveld fit performed on the PXRD pattern of TCP after Monte Carlo/simulated annealing ($R_{wp}$ = 14.23%, $R_p$ = 9.04%, $U_{iso}$ = 0.019) with the observed pattern in red, refined profile in blue, difference plot in black, and Bragg peak positions in green, and a structural refinement using unit-cell parameters and atomic coordinates for TCP in CIF format.

### 6.3 Grazing incidence diffraction (vacuum-deposited thin-films)

Grazing incidence diffraction (GID) was performed on a PANalytical Empyrean equipped with a Cu anode (K-Alpha1 wavelength = 1.540598; K-Alpha2 wavelength = 1.544426; Ratio K-Alpha2/K-Alpha1 = 0.5) operated at 45 kV, 40 mA with divergence slit set at 0.76mm. The samples were placed on a reflection-transmission spinner, and step size was set to 0.02˚.

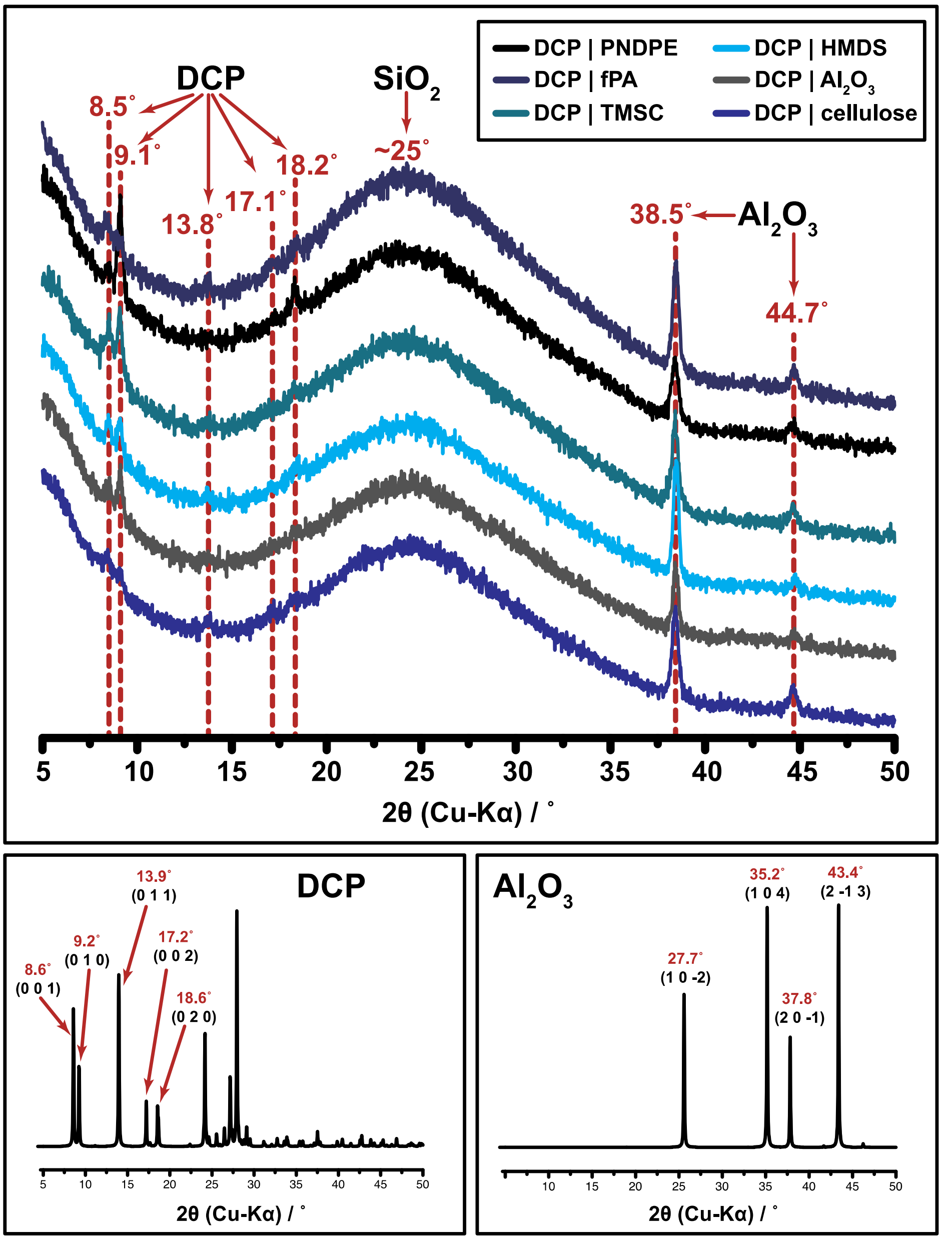


**Figure S14:** GID of DCP thin-films. Top: Superposition of diffractograms of DCP thin-films on different dielectrics (scattering curves have been vertically translated for clarity). All reflections can be assigned to either DCP, $SiO_2$ or $Al_2O_3$ (corundum). Bottom: Scattering curves of DCP and $Al_2O_3$ for comparison.

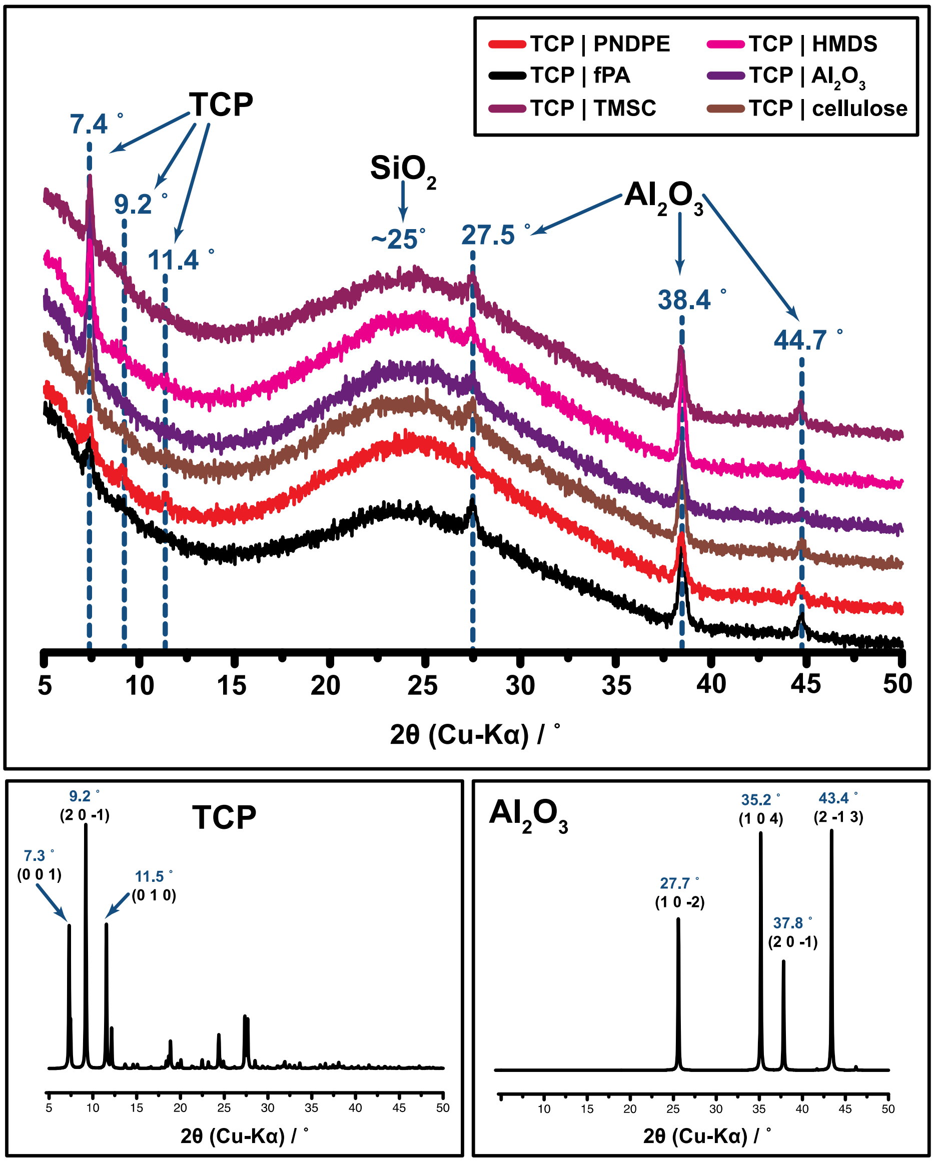


**Figure S15:** GID of TCP thin-films. Top: Superposition of diffractograms of TCP thin-films on different dielectrics (scattering curves have been vertically translated for clarity). All reflections can be assigned to either TCP, $SiO_2$ or $Al_2O_3$ (corundum). Bottom: Scattering curves of TCP (from Pawley refinement) and $Al_2O_3$ for comparison.

## 7. Theoretical calculations

Theoretical calculations (all performed at performed at HF-6-311G(d,p) level) of DCP and TCP in the different crystal structures – specifically electrostatic potential (ESP) isosurfaces (isovalue = 0.008 e/ang$^3$), Hirshfeld surfaces mapped with $d_{norm}$ (Figure S14 A) and 2D Hirshfeld fingerprint plots (Figure S14 B-H), as well as electron density isosurfaces (isovalue 0.008 e/ang$^3$) mapped with $d_{norm}$ – were generated using CrystalExplorer 3.1.[S9]

The $d_{norm}$ property is a normalized contact distance calculated according to Equation S1. $d_i$ is the distance from the surface to the nearest nucleus inside the surface, $d_e$ to the nearest nucleus outside the surface. $r_i^{vdW}$ and $r_e^{vdW}$ are the van der Waals radii of the atoms involved inside and outside the surface. Mapping $d_{norm}$ onto the surface indicates contacts shorter than van der Waals contacts in red, contacts around the van der Waals contacts in white and contacts longer than the van der Waals contacts in blue.

$$d_{norm} = \frac{d_i - r_i^{vdW}}{r_i^{vdW}} + \frac{d_e - r_e^{vdW}}{r_e^{vdW}}$$

**Equation S1**: Calculation of the $d_{norm}$ property

### 7.1 Hirshfeld Surface and Fingerprint Plot

The non-covalent interactions (NCIs), *i.e.* both π-stacks and H-bonds, are illustrated by the Hirshfeld surface and fingerprint plots,[S10] of a TCP molecule within the crystal structure of $TCP{\cdot}PhNO_2$ (Figure S16). C···C, C···H, C···N, and N···N close contacts account for 25%, 24.7%, 11.4%, and 0.6% of the Hirshfeld surface area (Figure S16 C-F), respectively. These close-contacts arise mostly (C···H, C···N) or exclusively (C···C, N···N) from π-π interactions with TCP molecules beneath and atop (intracolumn), as underlined by their position on the Hirshfeld surface (insets in Figure S16 C-F). The C···H and C···N close-contacts are in part arising from interactions with disordered $PhNO_2$ (Figure S16 D,E). N···H close-contacts (Figure S16 H, 28%) mainly reflect the intermolecular H-bonding between the nitrile N and $C_{Ar}$-H.

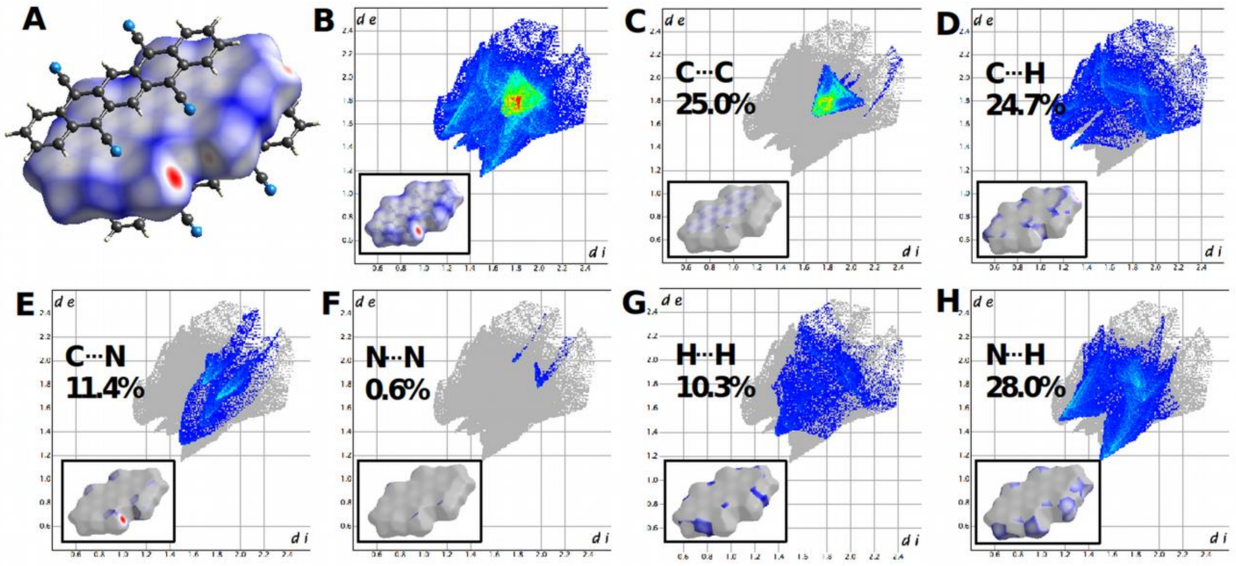

**Figure S16:** Hirshfeld surface and finger print plots of TCP in TCP·$PhNO_2$

Since the “pure” TCP structure was obtained *via* refinement and not solved from single crystal data, Hirshfeld surface analysis could not be performed with sufficient accuracy.

## 8. Optical microscopy and scanning electron microscopy

### 8.1. Optical microscopy

Optical microscopy was performed on a LEICA M 125 microscope, images were taken using the software LAS v3.7.

### 8.2. Scanning electron microscopy

Scanning electron microscopy was carried out with a Quanta 200F FEImicroscope. Typically the samples were measured at 5 or 10 kV, with a working distance of 9-10 mm and spot size 2.0. Prior to imaging, powder samples were loaded on carbon coated stubs and coated by sputtering with a 17 nm thick layer of Au/Pd 60/40 alloy with a Quarum Q105T S sample preparation system.

The thin-films on different dielectrics were prepared under the same conditions as for the transistor fabrication. Thin-film samples were equally placed on carbon-coated stubs and sputtered as described above.

### *8.2.1 DCP and TCP thin-films on different dielectrics*

For an overview of the tested dielectrics see Table S2.

*DCP on $Al_2O_3$:*

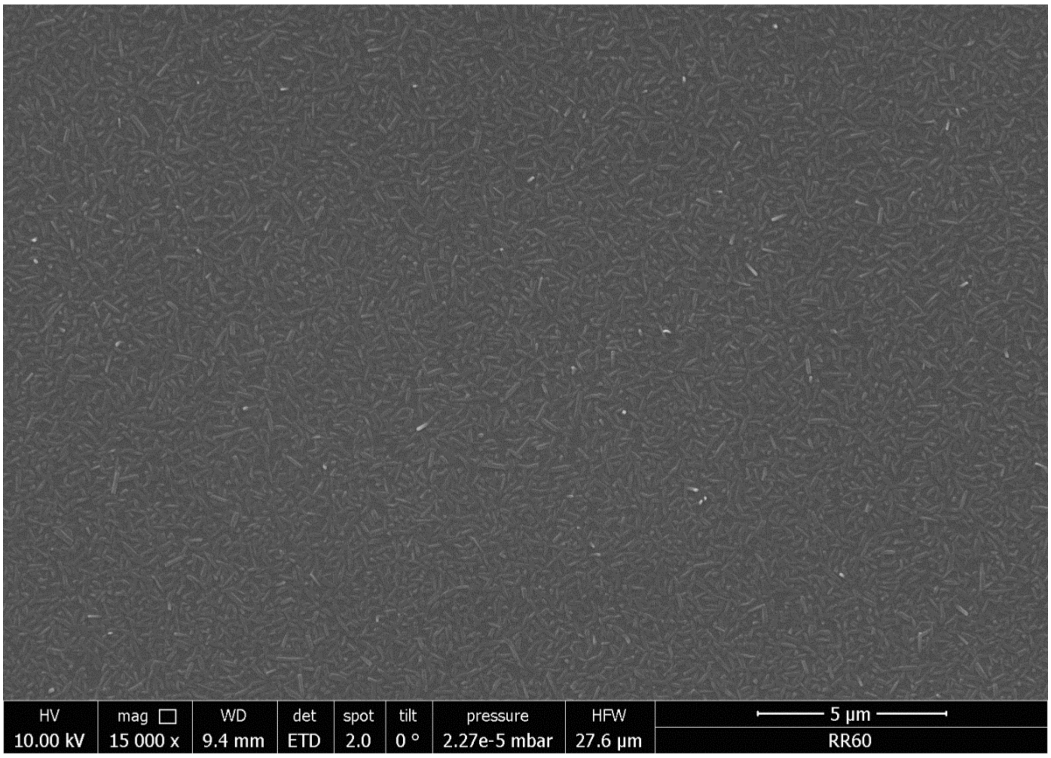


**Figure S17:** SEM image of DCP on $Al_2O_3$ (I)

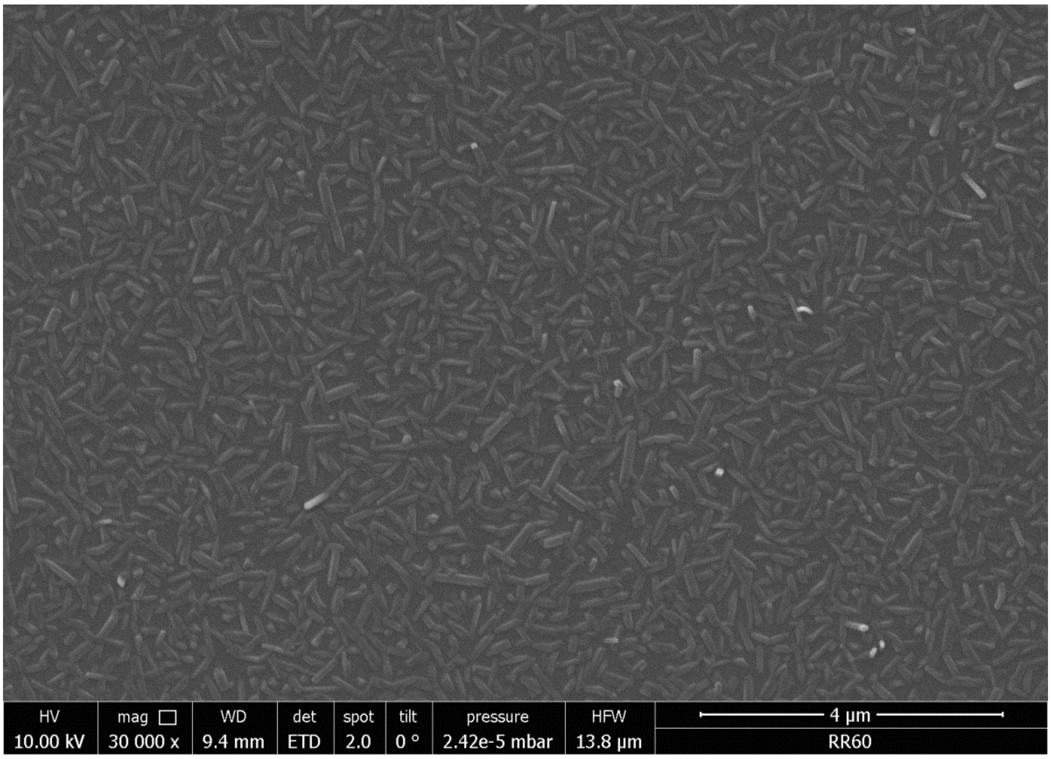


**Figure S18:** SEM image of DCP on $Al_2O_3$ (II)

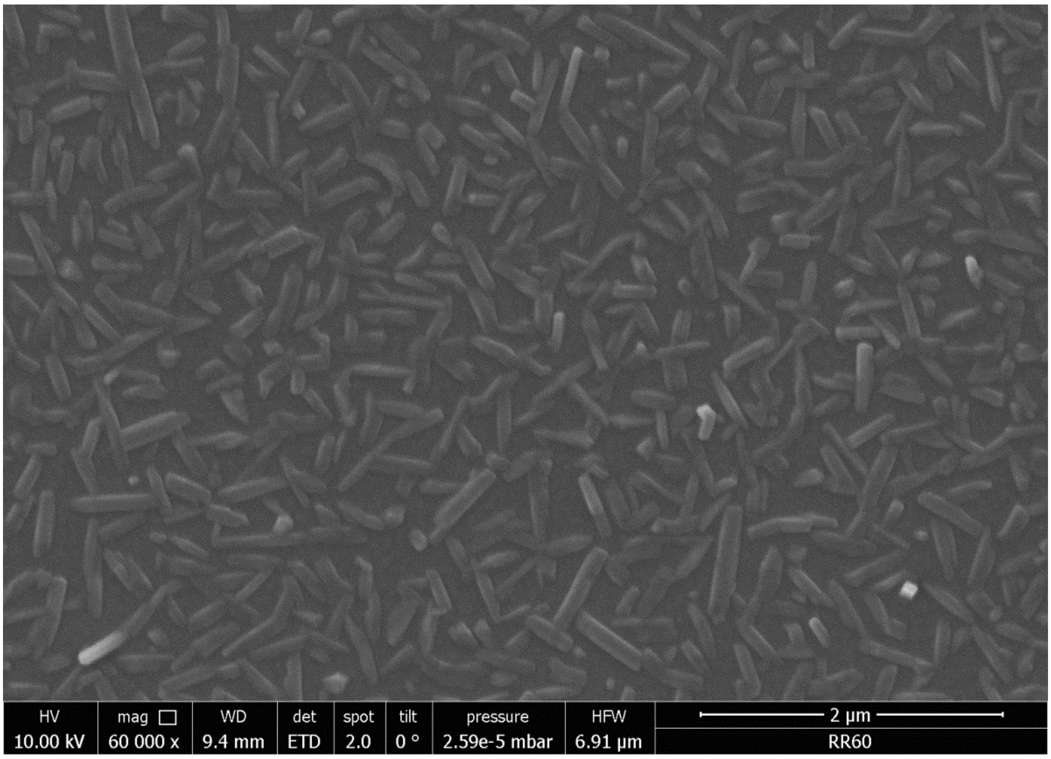


**Figure S19:** SEM image of DCP on $Al_2O_3$ (III)

*DCP on $Al_2O_3$ modified with a layer of PNDPE (poly((±)endo,exo-bicyclo[2.2.1]hept-5-ene-2,3-dicarboxylic acid, diphenylester):*

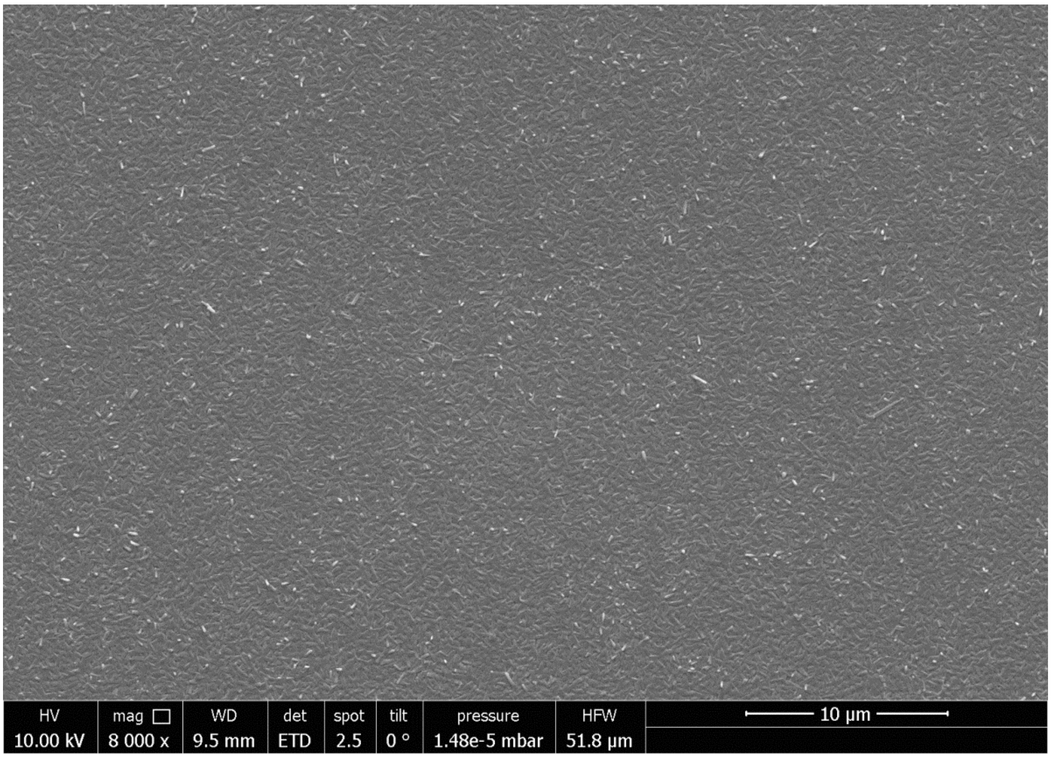


**Figure S20:** SEM image of DCP on $Al_2O_3$ modified with a layer of PNDPE (I)

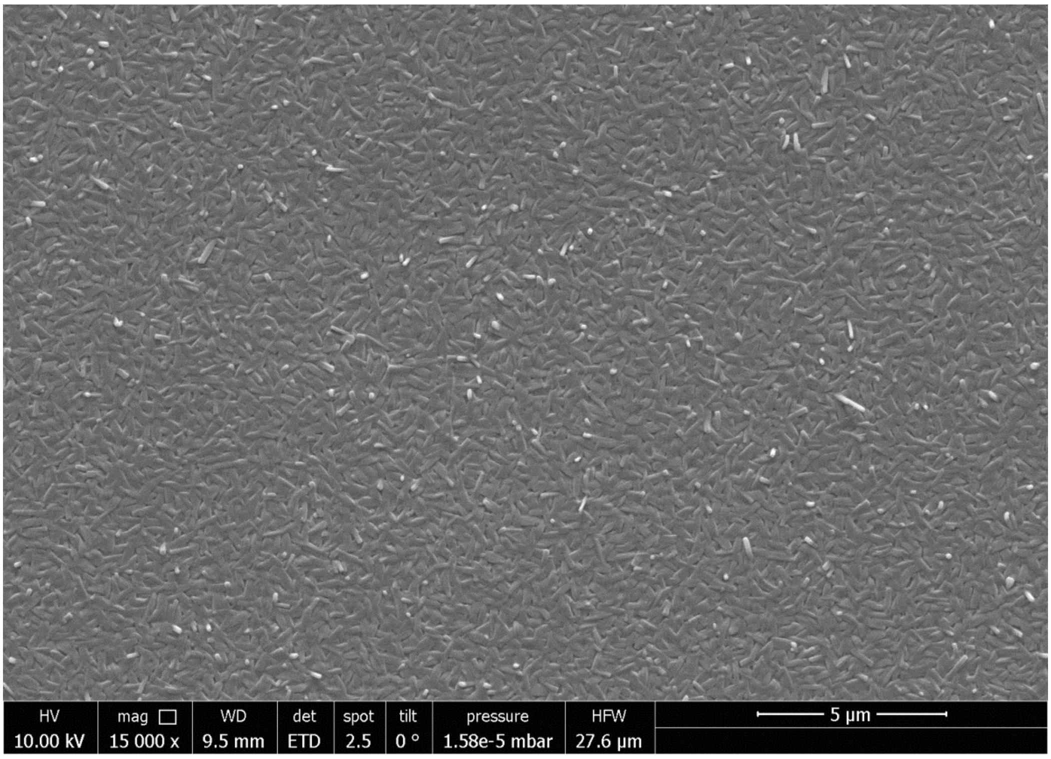


**Figure S21:** SEM image of DCP on $Al_2O_3$ modified with a layer of PNDPE (II)

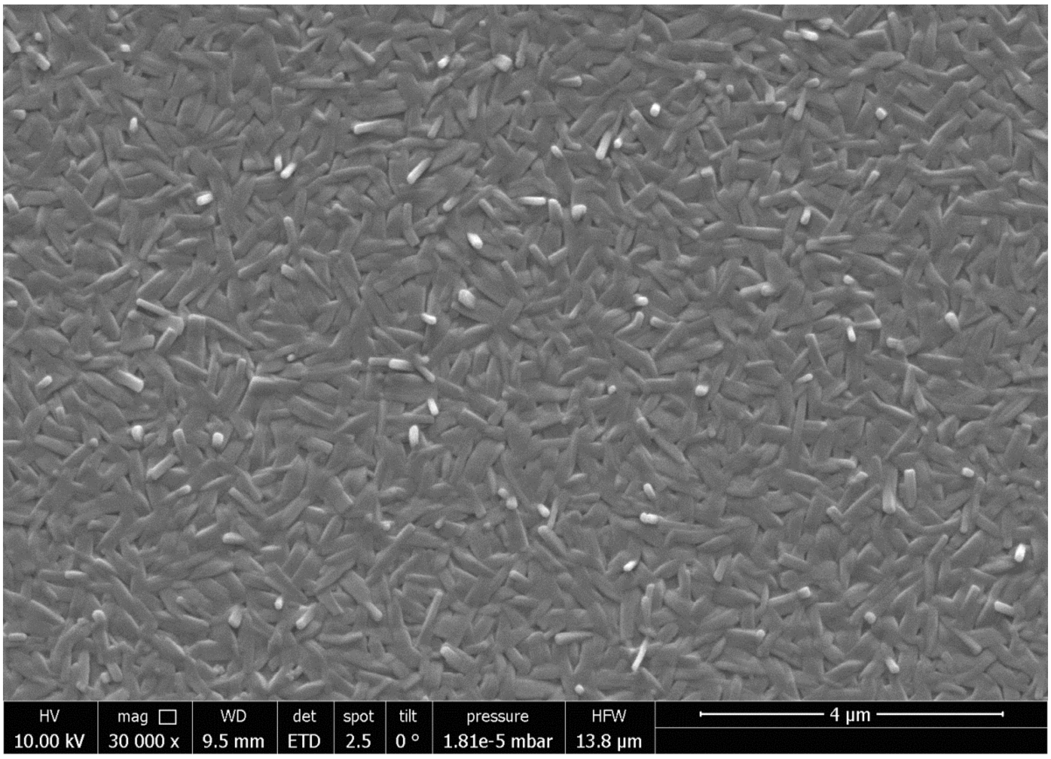


**Figure S22:** SEM image of DCP on $Al_2O_3$ modified with a layer of PNDPE (III)

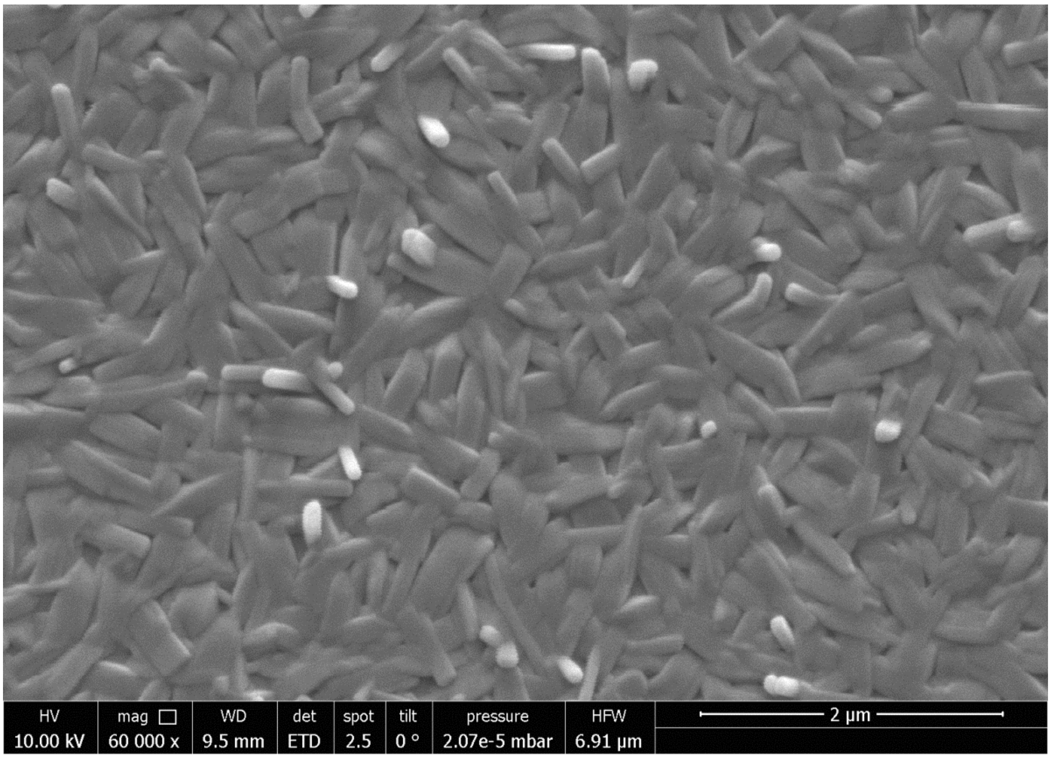


**Figure S23:** SEM image of DCP on $Al_2O_3$ modified with a layer of PNDPE (IV)

*DCP on $Al_2O_3$ modified with a self-assembled monolayer of fPA (fluoroalkyl phosphonic acid):*

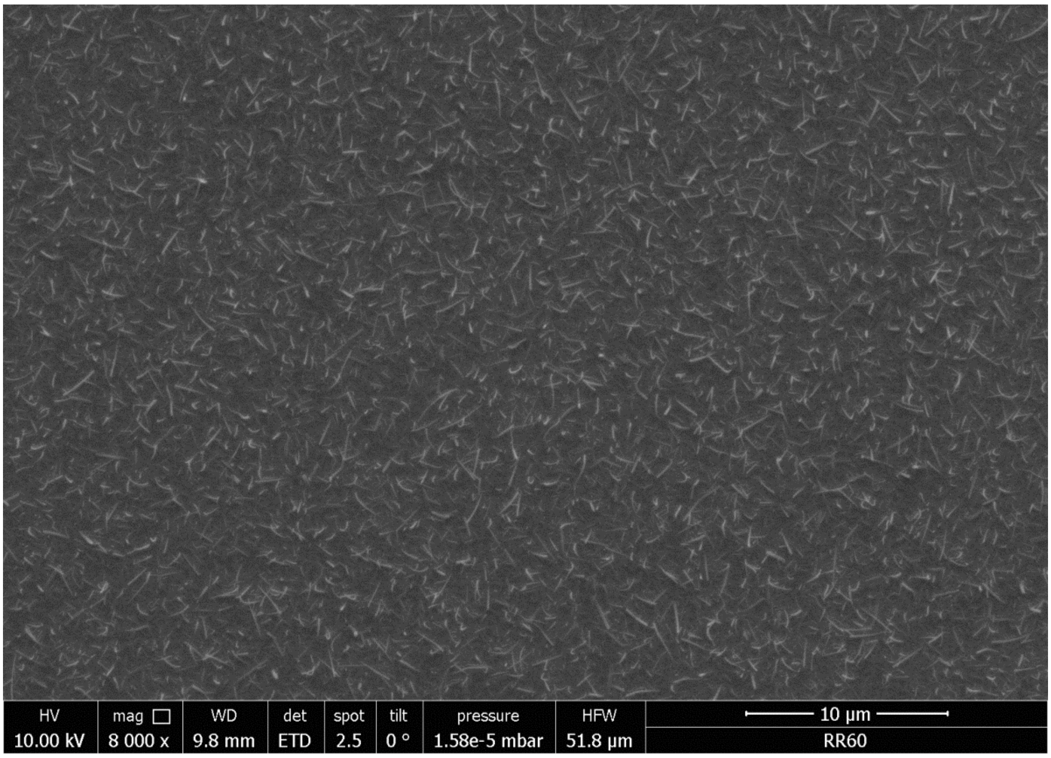


**Figure S24:** SEM image of DCP on $Al_2O_3$ modified with a self-assembled monolayer of fPA (I)

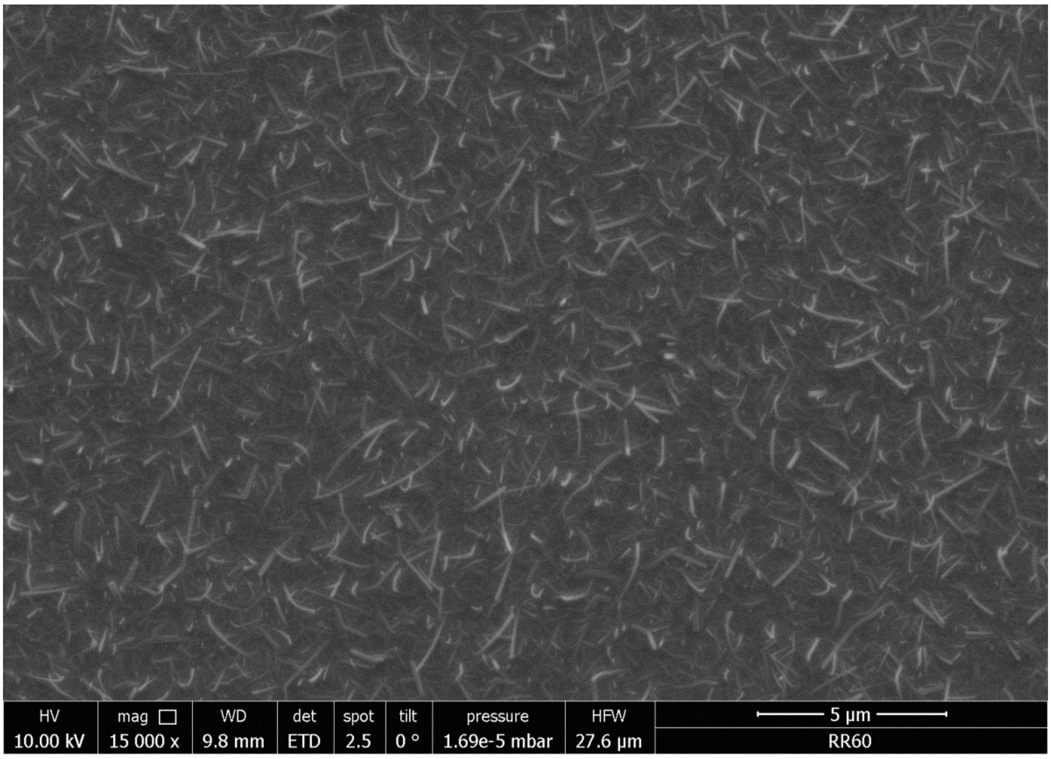


**Figure S25:** SEM image of DCP on $Al_2O_3$ modified with a self-assembled monolayer of fPA (II)

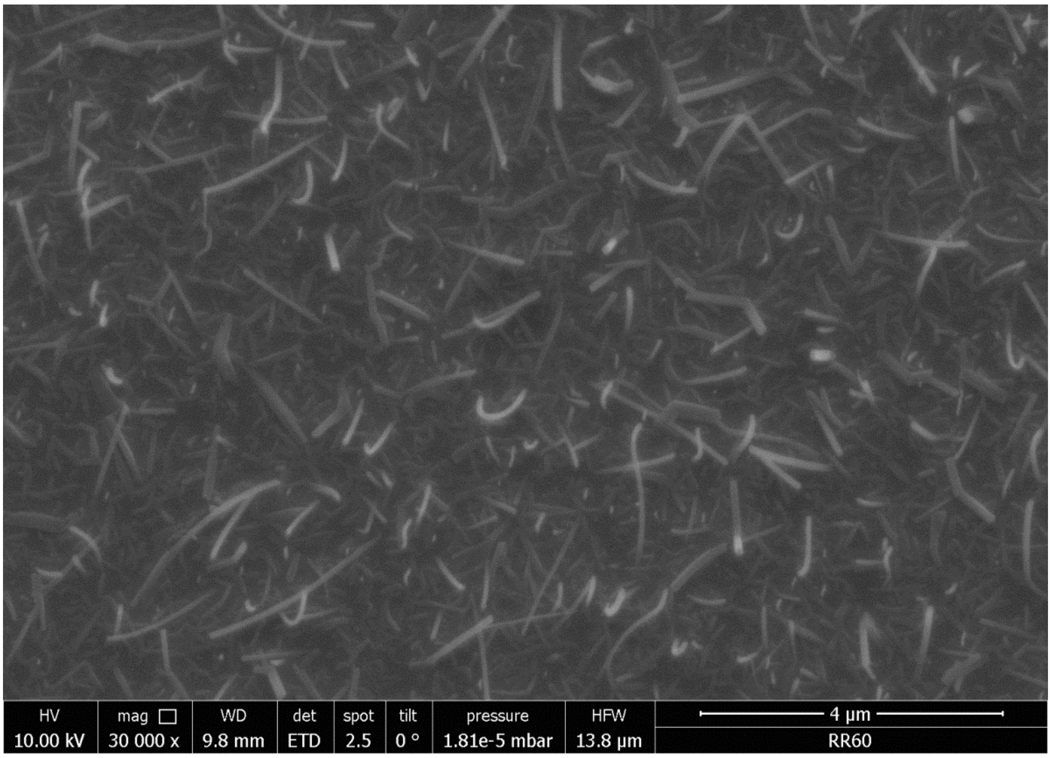


**Figure S26:** SEM image of DCP on $Al_2O_3$ modified with a self-assembled monolayer of fPA (III)

*DCP on $Al_2O_3$ modified with a layer of cellulose:*

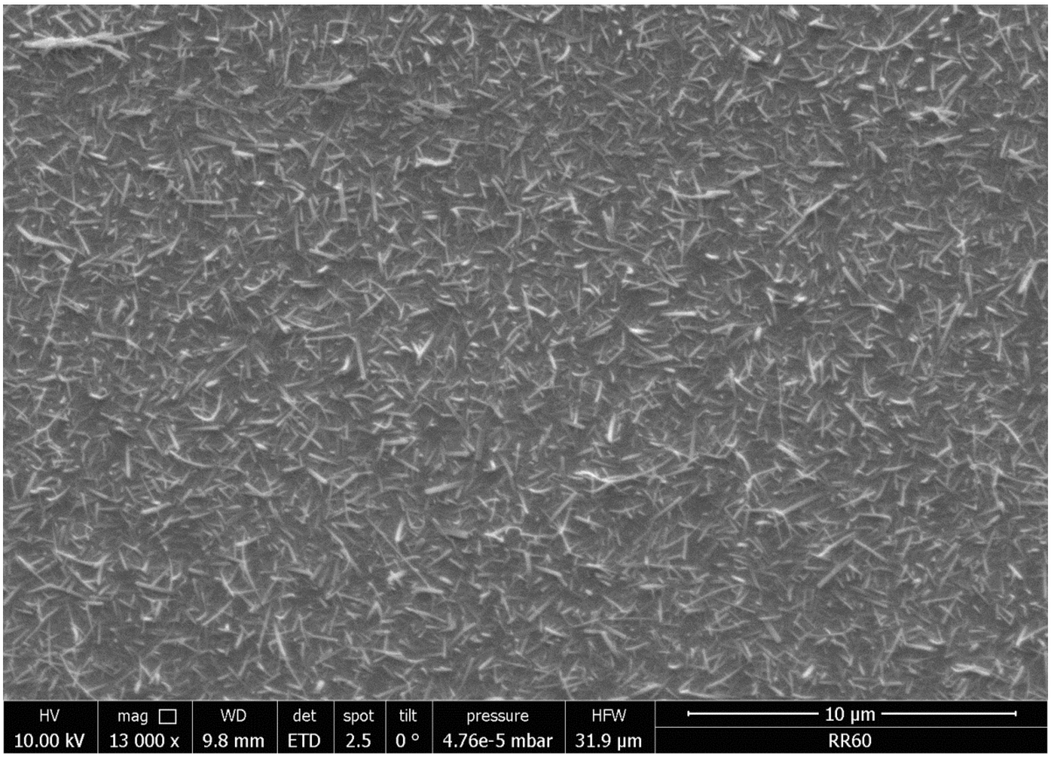


**Figure S27:** SEM image of DCP on $Al_2O_3$ modified with a layer of cellulose (I)

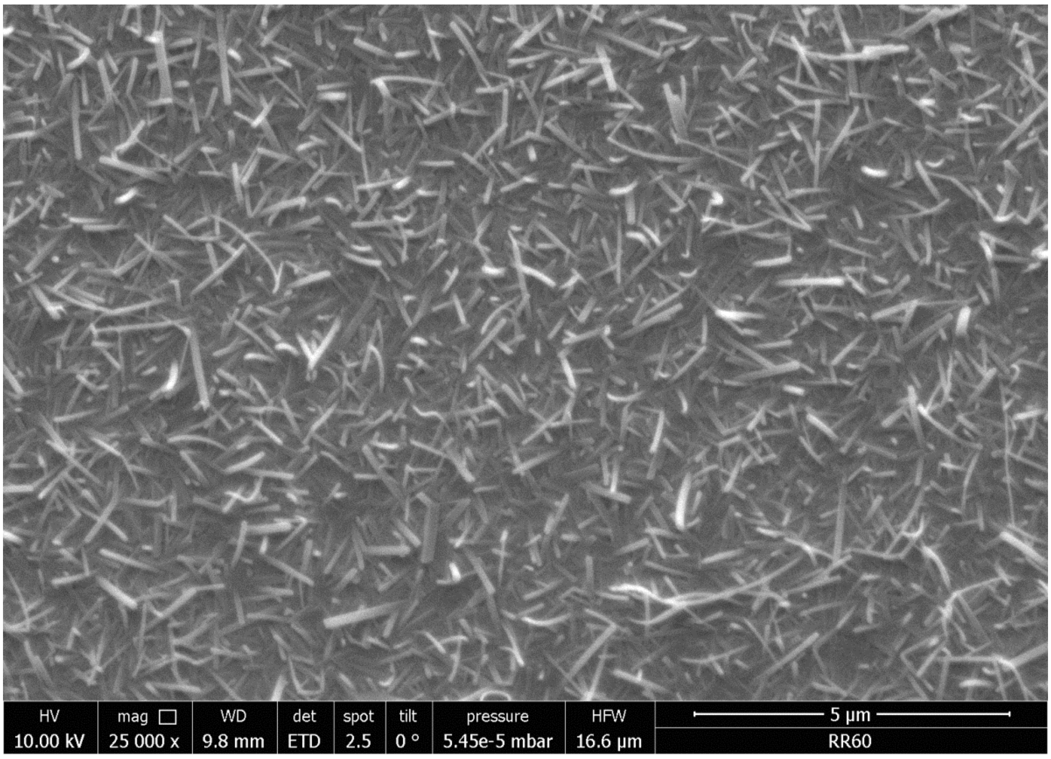


**Figure S28:** SEM image of DCP on $Al_2O_3$ modified with a layer of cellulose (II)

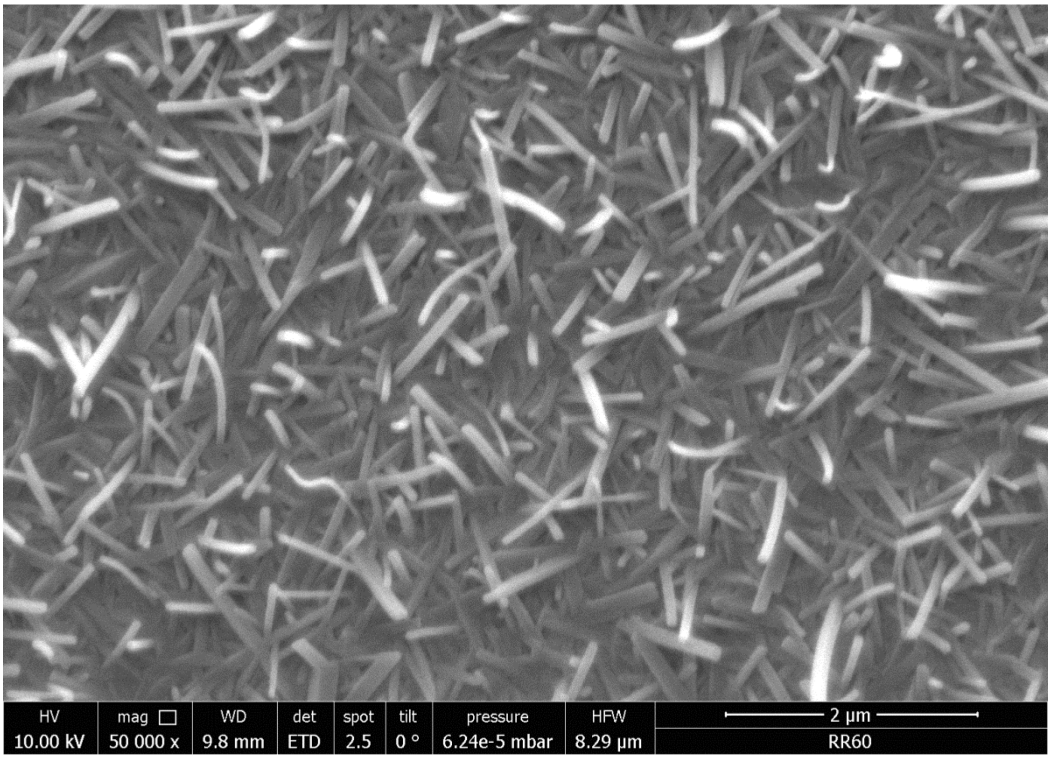


**Figure S29:** SEM image of DCP on $Al_2O_3$ modified with a layer of cellulose (III)

*DCP on $Al_2O_3$ modified with a layer of TMSC (trimethylsilylcellulose)*:

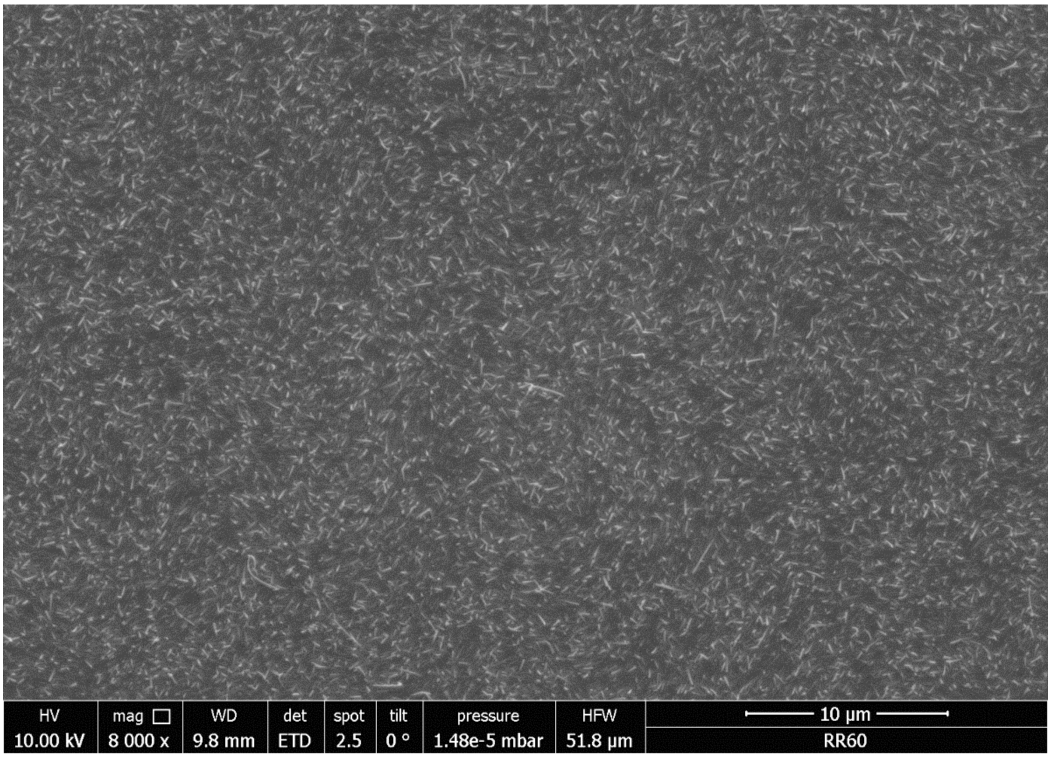


**Figure S30:** SEM image of DCP on $Al_2O_3$ modified with a layer of TMSC (I)

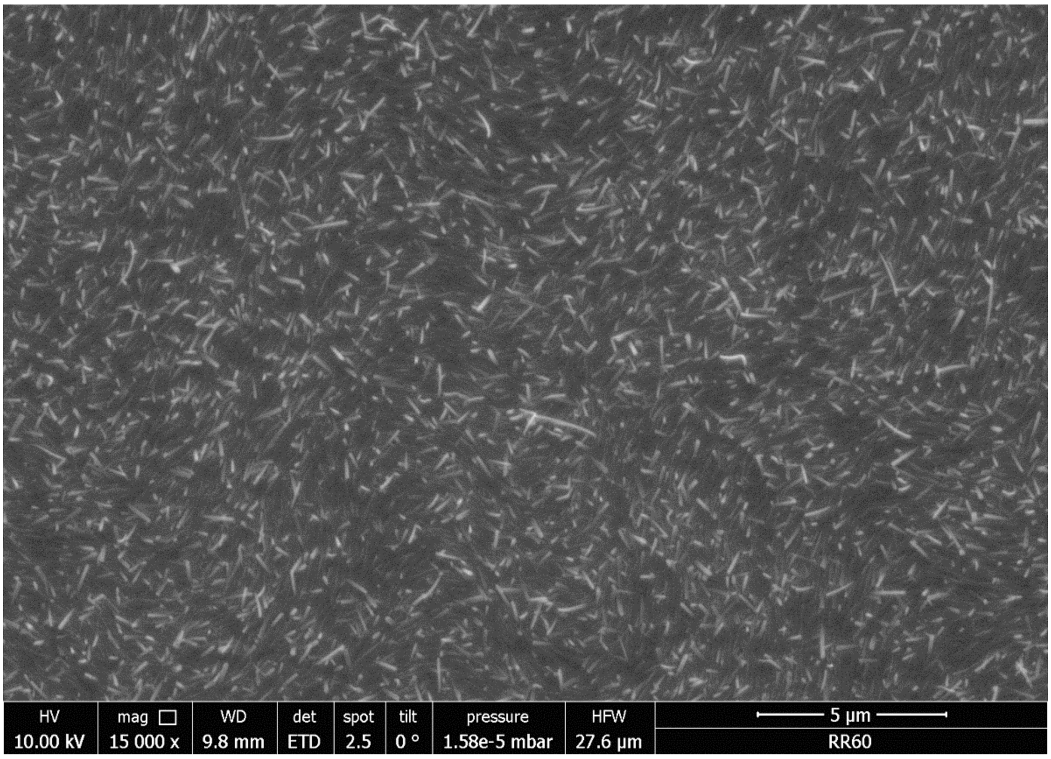


**Figure S31:** SEM image of DCP on $Al_2O_3$ modified with a layer of TMSC (II)

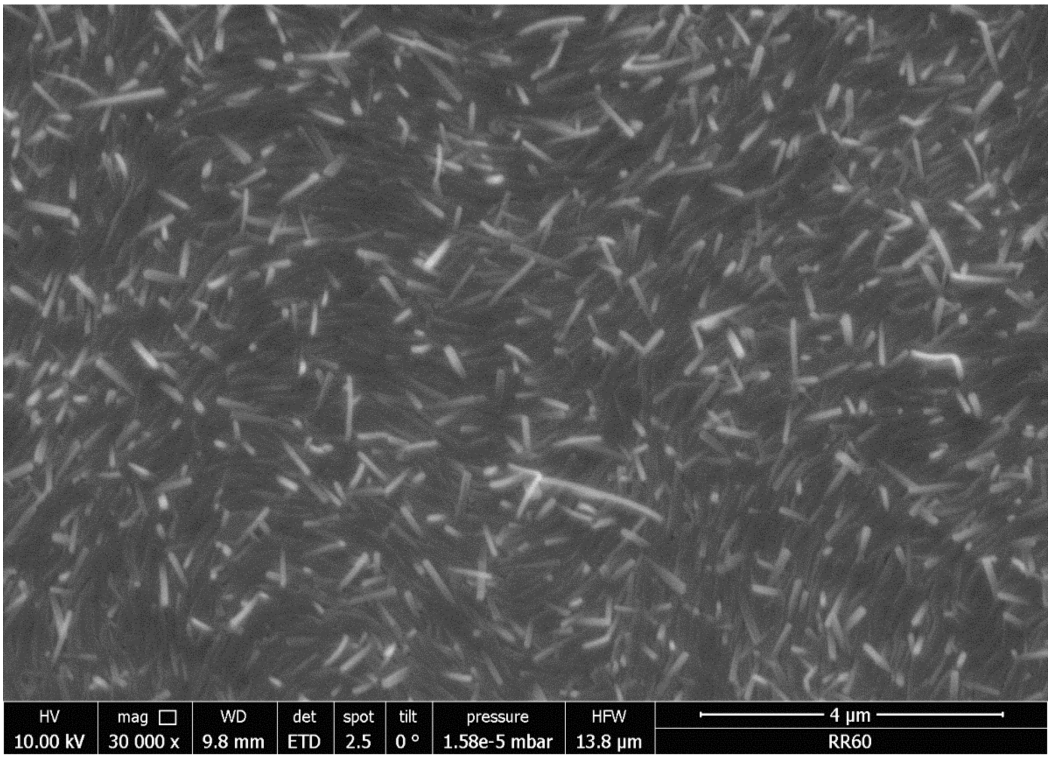


**Figure S32:** SEM image of DCP on $Al_2O_3$ modified with a layer of TMSC (III)

*TCP on $Al_2O_3$:*

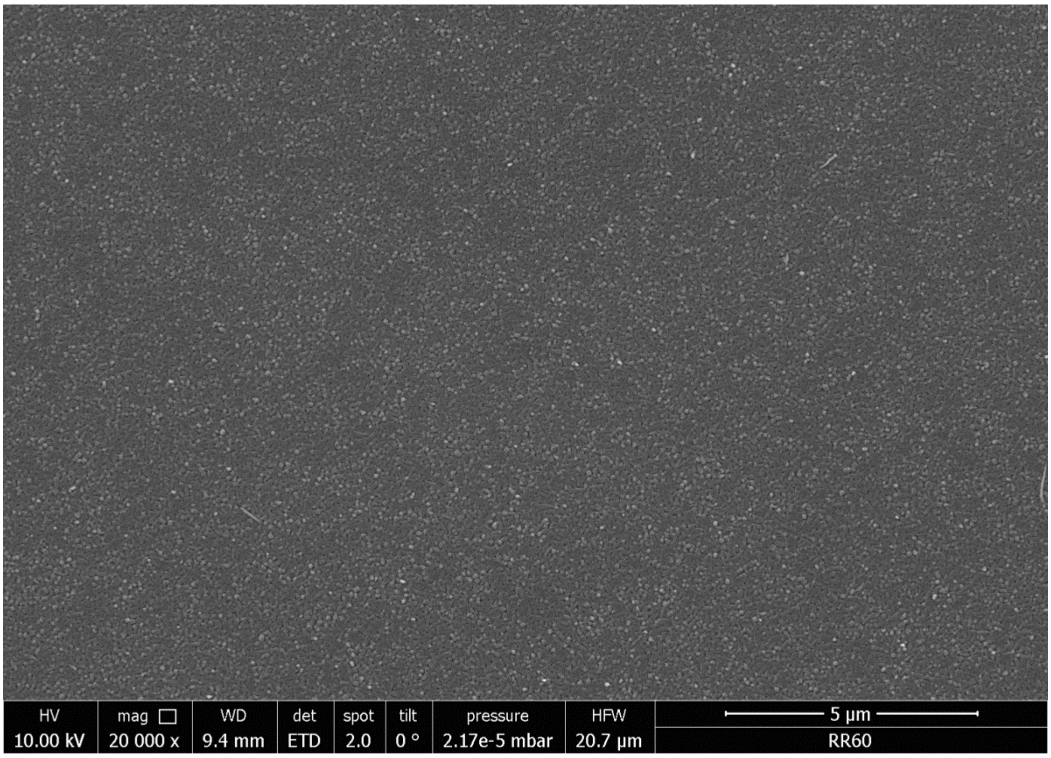


**Figure S33:** SEM image of TCP on $Al_2O_3$ (I)

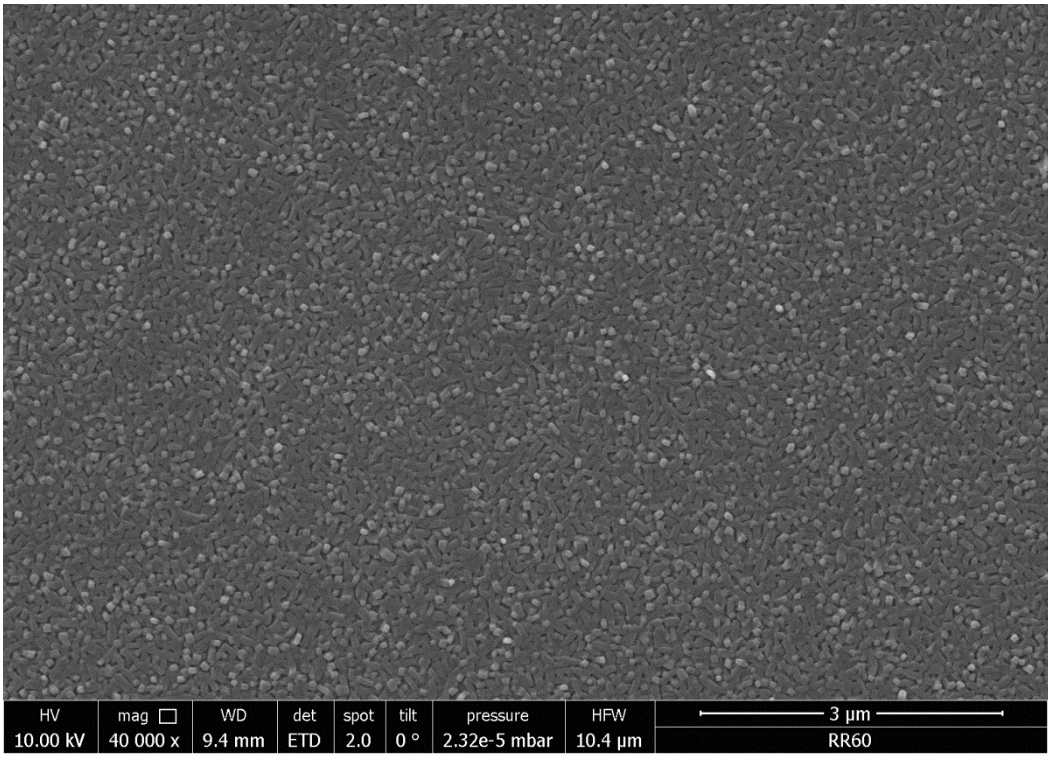


**Figure S34:** SEM image of TCP on $Al_2O_3$ (II)

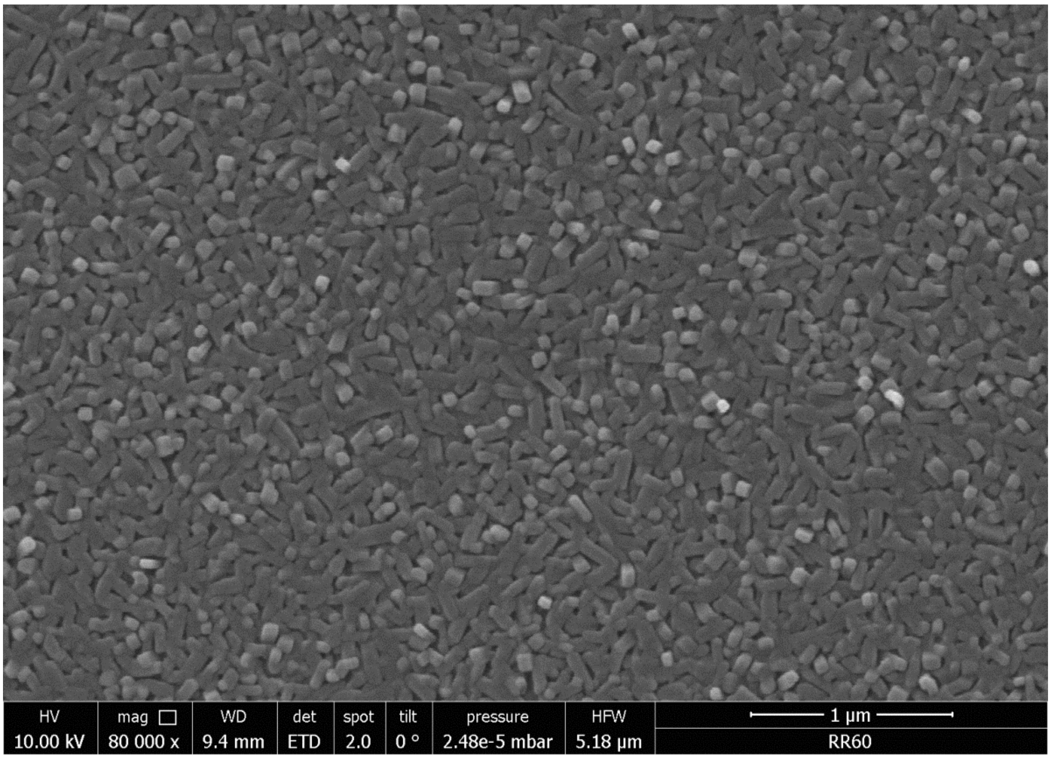


**Figure S35:** SEM image of TCP on $Al_2O_3$ (III)

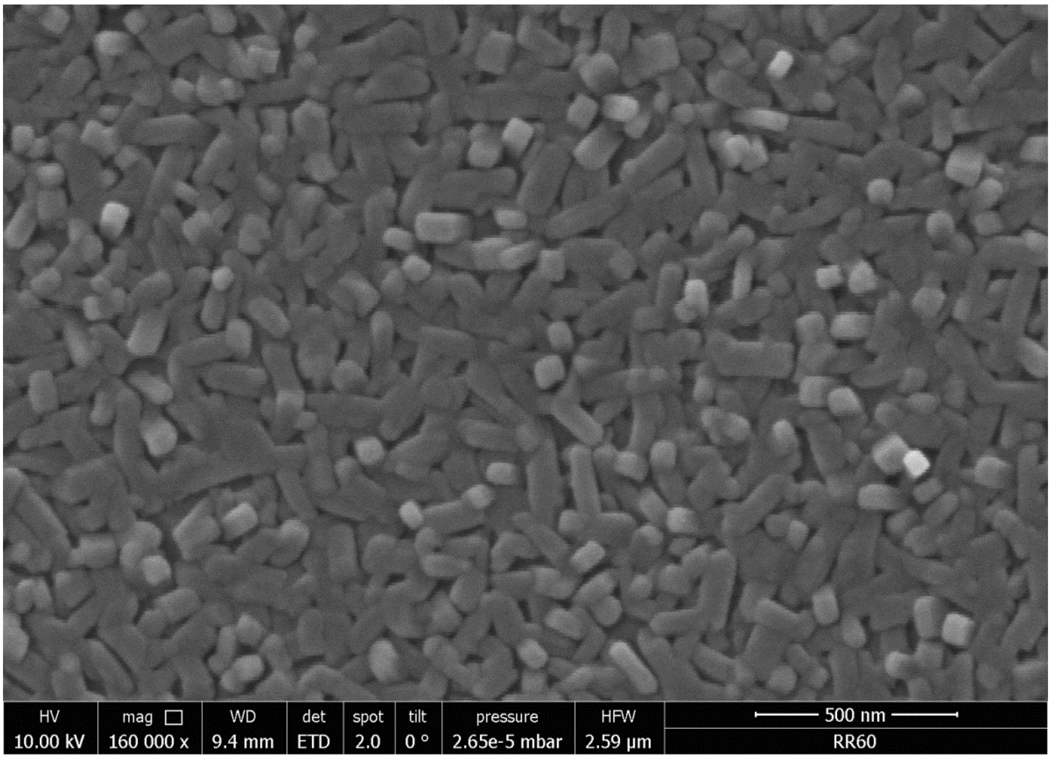


**Figure S36:** SEM image of TCP on $Al_2O_3$ (IV)

*TCP on $Al_2O_3$ modified with a layer of PNDPE (poly((±)endo,exo-bicyclo[2.2.1]hept-5-ene-2,3-dicarboxylic acid, diphenylester):*

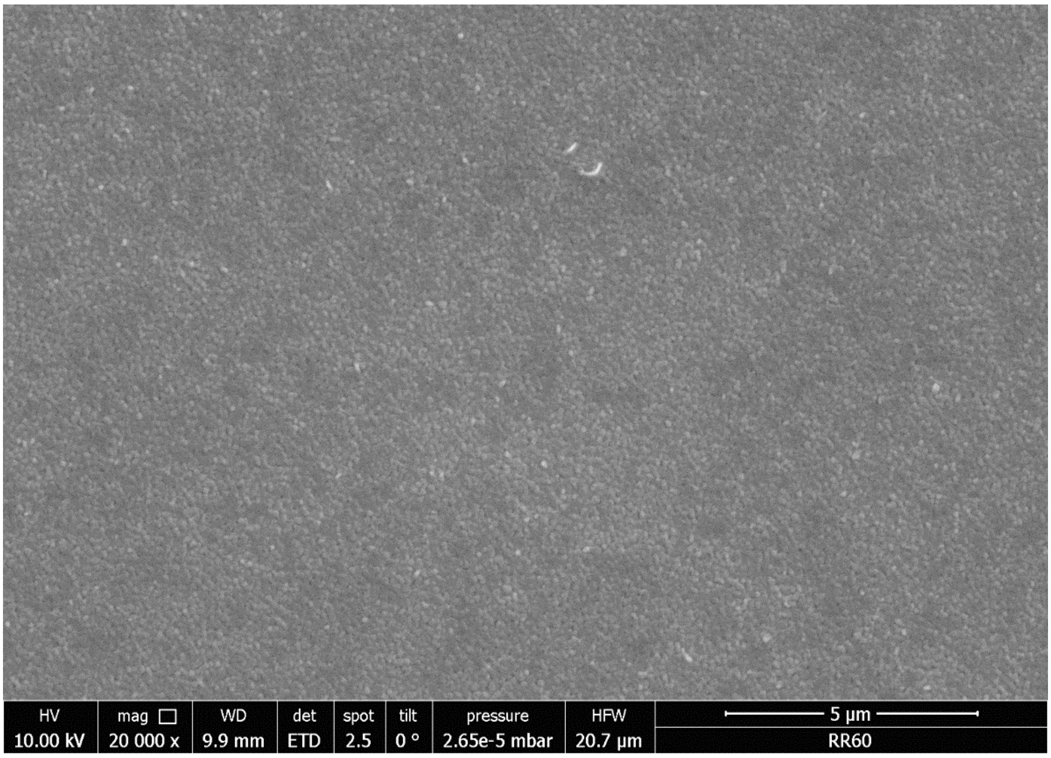


**Figure S37:** SEM image of TCP on $Al_2O_3$ modified with a layer of PNDPE (I)

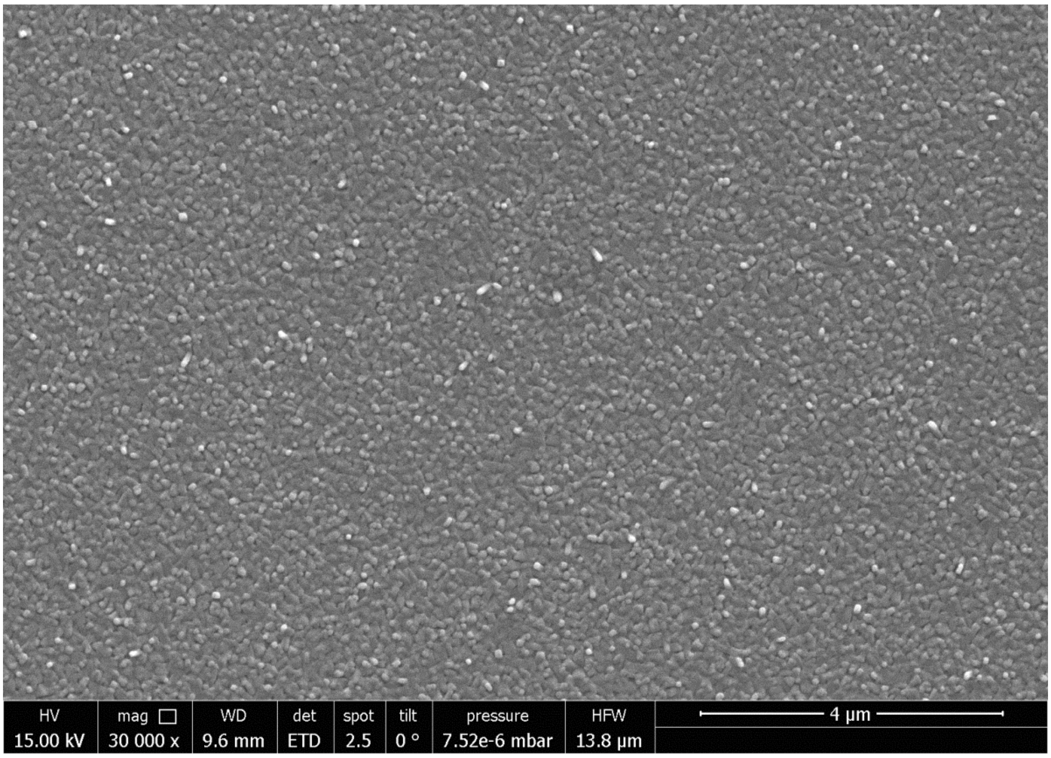


**Figure S38:** SEM image of TCP on $Al_2O_3$ modified with a layer of PNDPE (II)

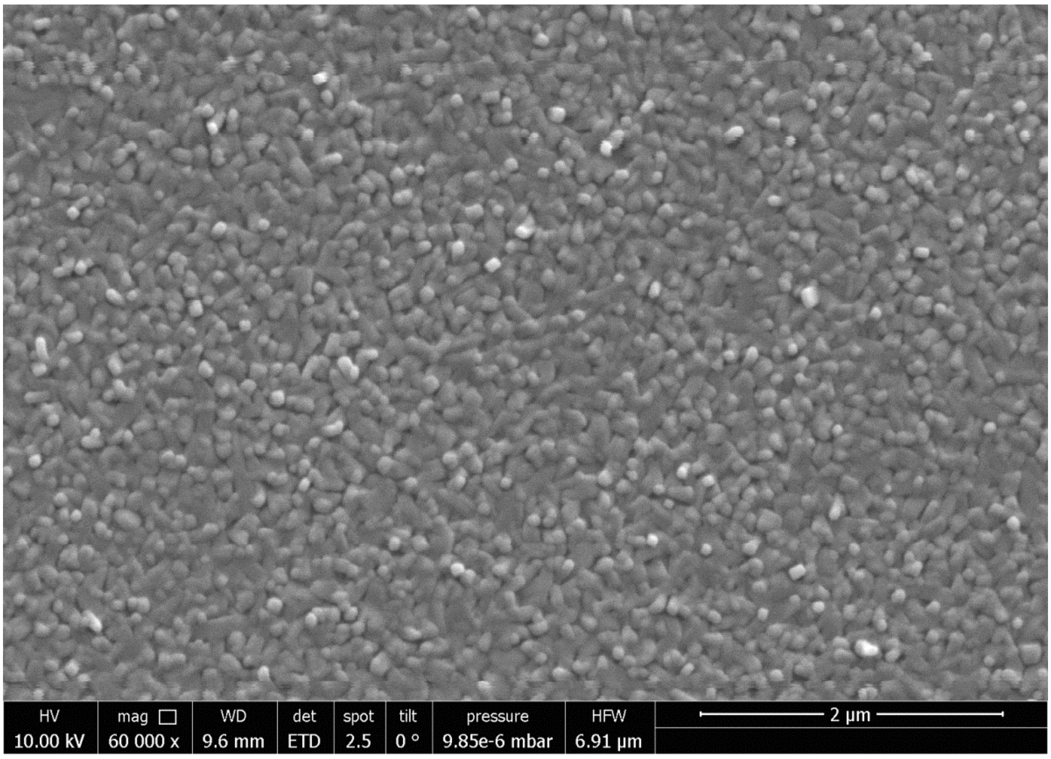


**Figure S39:** SEM image of TCP on $Al_2O_3$ modified with a layer of PNDPE (III)

*TCP on $Al_2O_3$ modified with a self-assembled monolayer of fPA (fluoroalkyl phosphonic acid):*

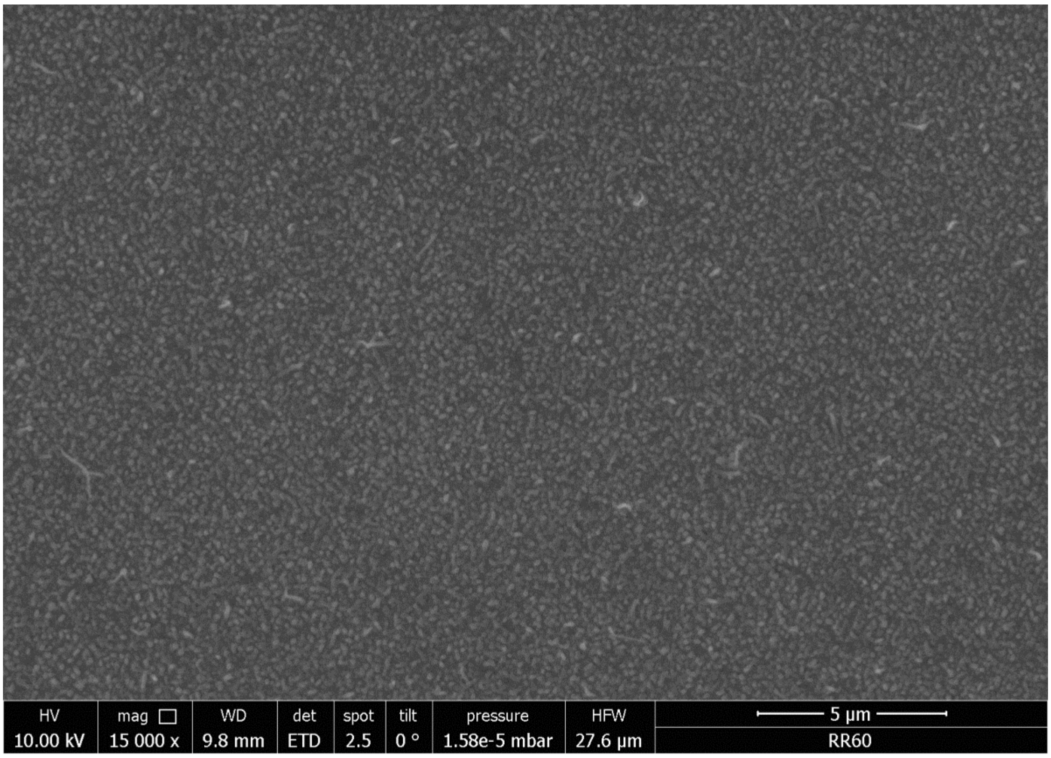


**Figure S40:** SEM image of TCP on $Al_2O_3$ modified with a self-assembled monolayer of fPA (I)

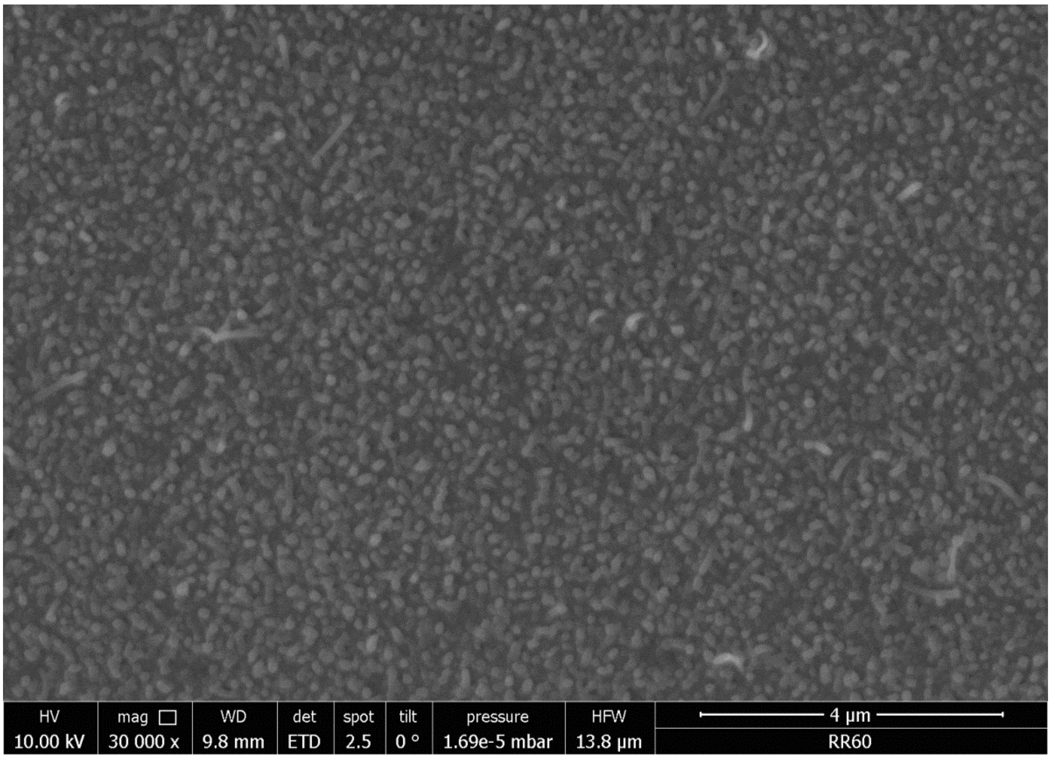


**Figure S41:** SEM image of TCP on $Al_2O_3$ modified with a self-assembled monolayer of fPA (II)

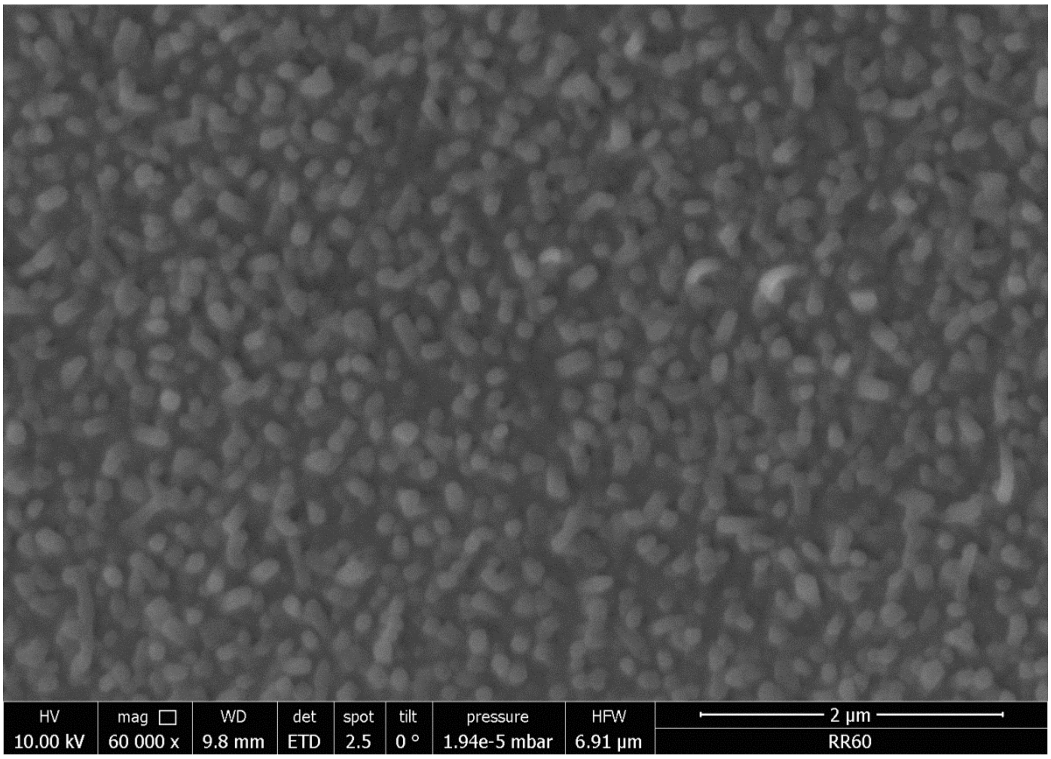


**Figure S42:** SEM image of TCP on $Al_2O_3$ modified with a self-assembled monolayer of fPA (III)

*TCP on $Al_2O_3$ modified with a layer of cellulose:*

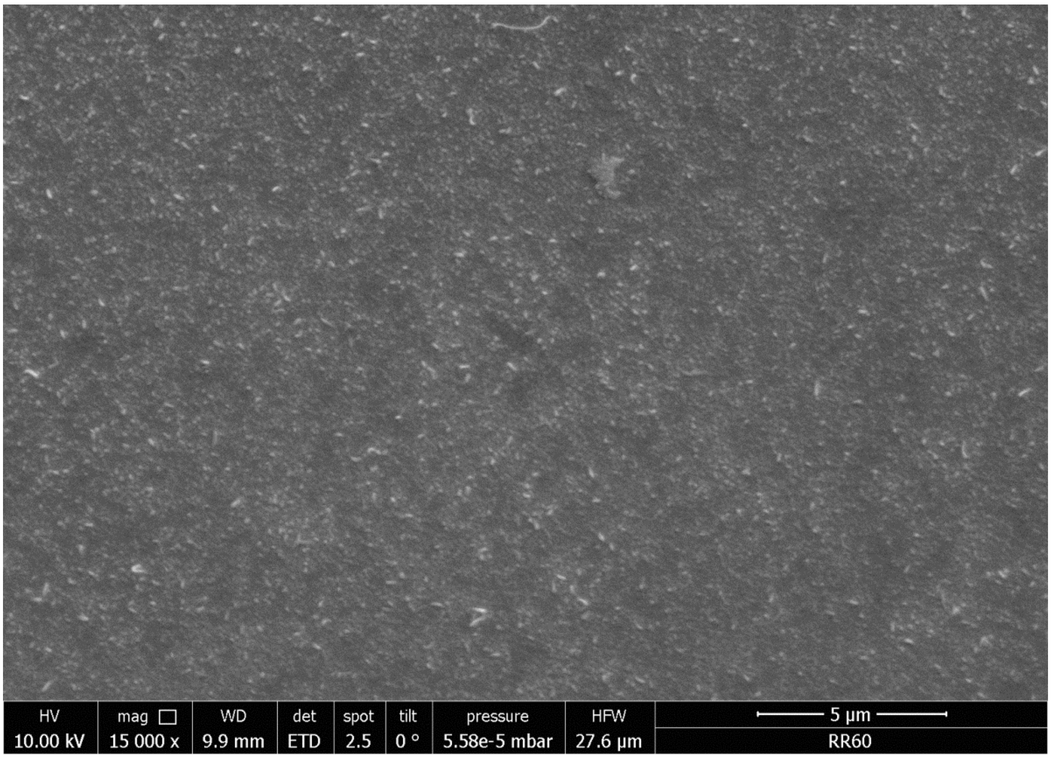


**Figure S43:** SEM image of TCP on $Al_2O_3$ modified with a layer of cellulose (I)

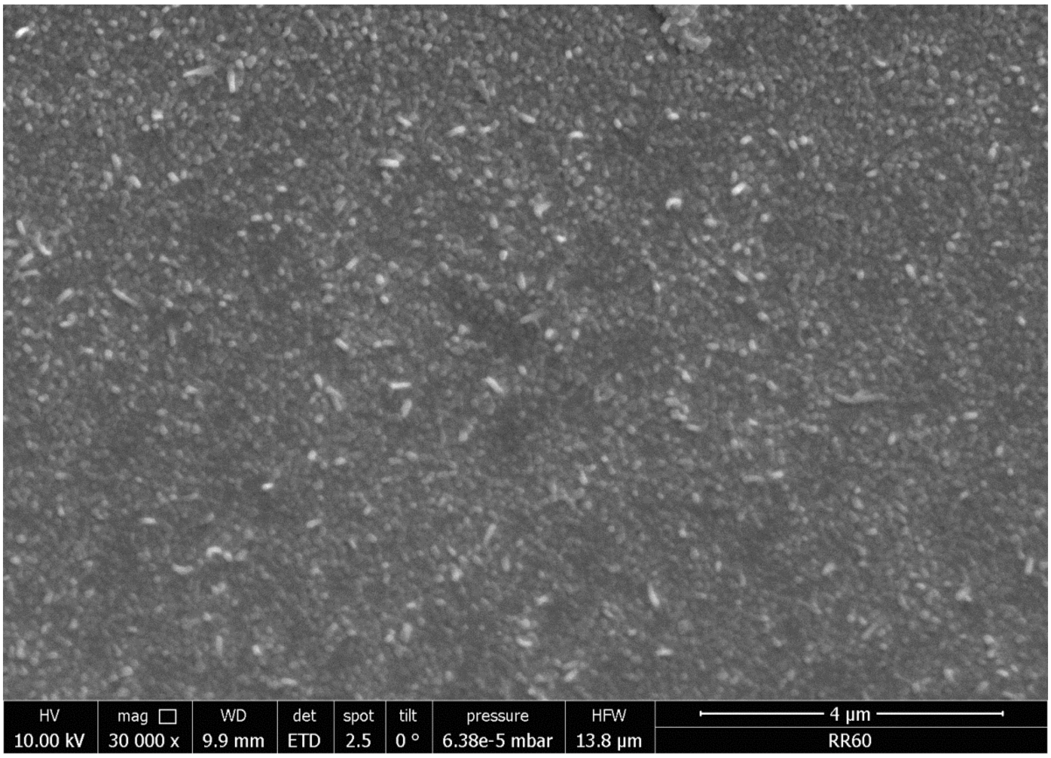


**Figure S44:** SEM image of TCP on $Al_2O_3$ modified with a layer of cellulose (II)

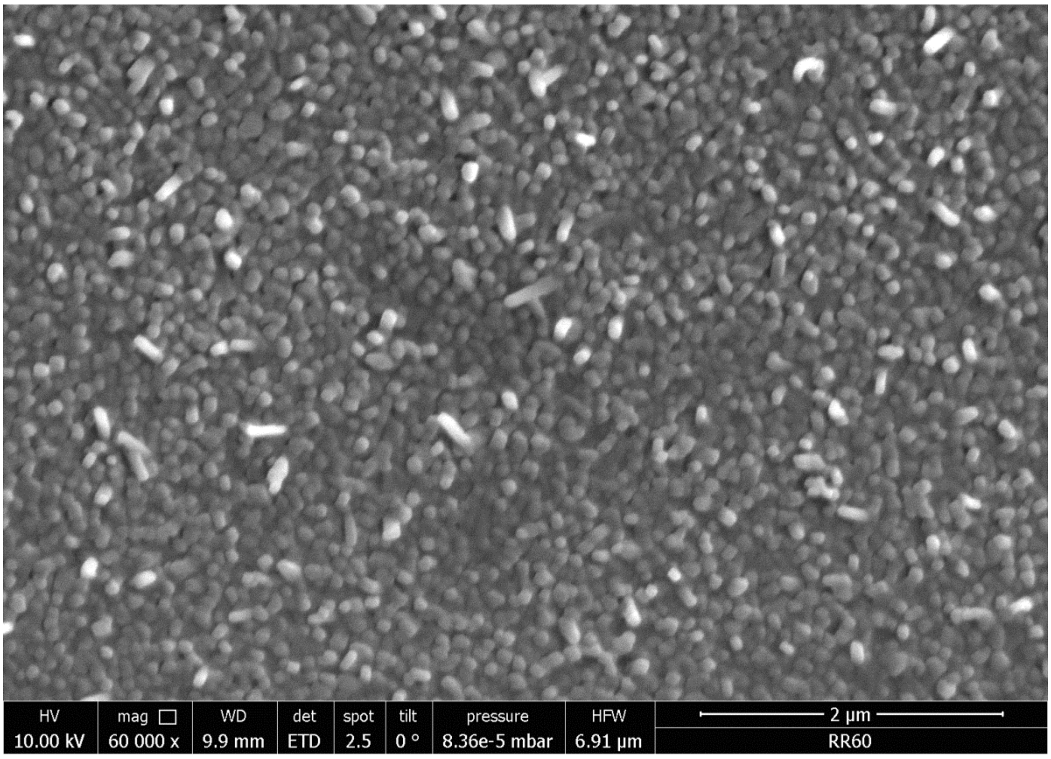


**Figure S45:** SEM image of TCP on $Al_2O_3$ modified with a layer of cellulose (III)

*TCP on $Al_2O_3$ modified with a layer of TMSC (trimethylsilylcellulose)*:

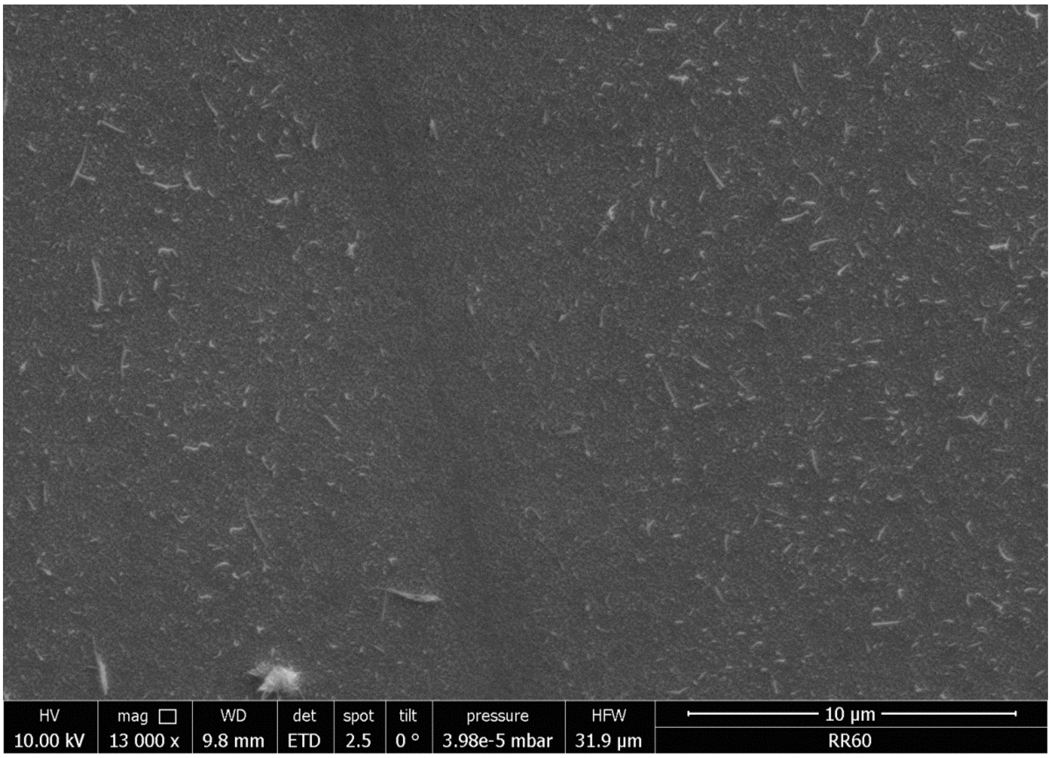


**Figure S46:** SEM image of TCP on $Al_2O_3$ modified with a layer of TMSC (I)

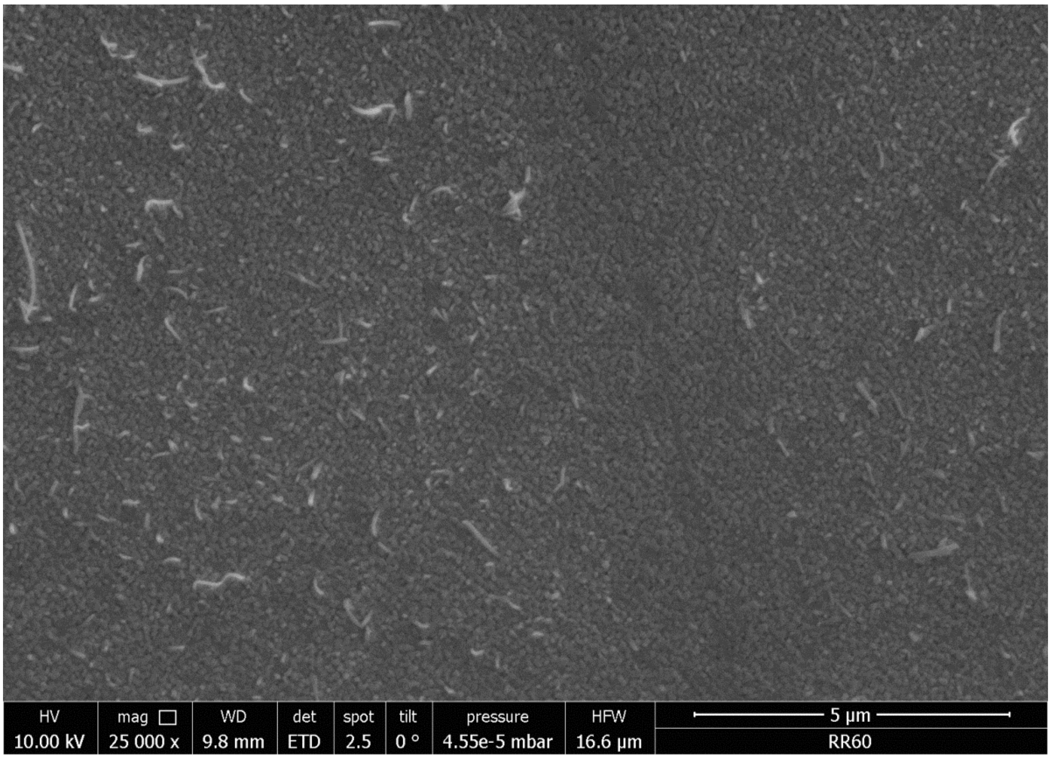


**Figure S47:** SEM image of TCP on $Al_2O_3$ modified with a layer of TMSC (II)

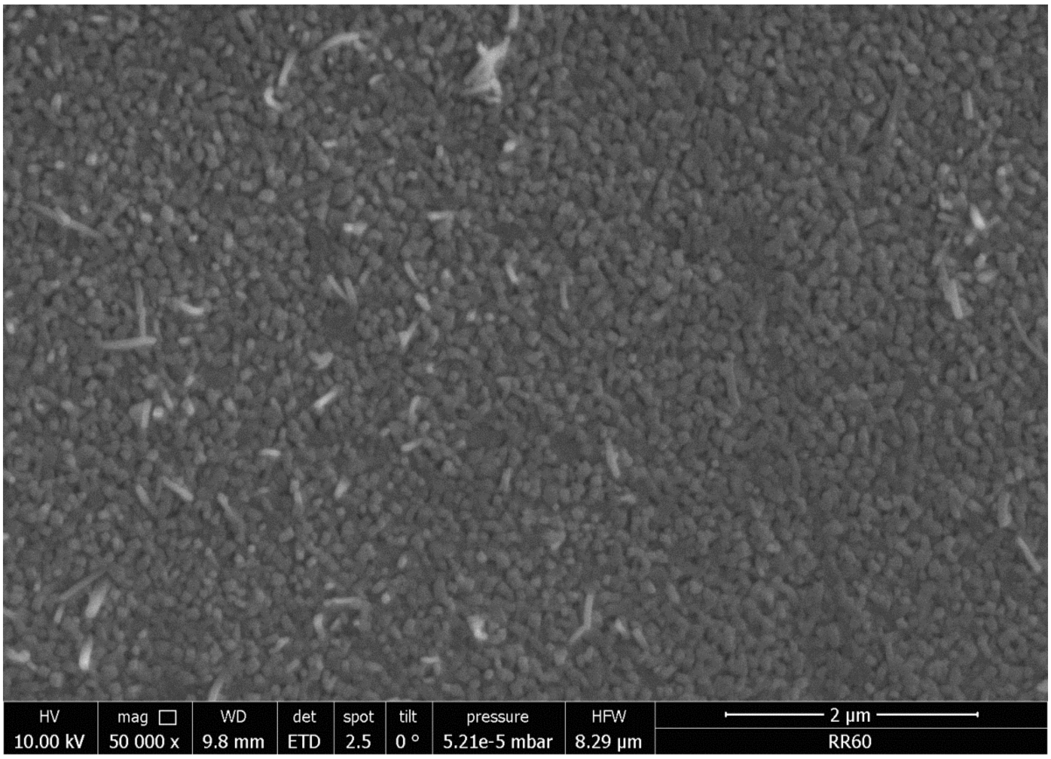


**Figure S48:** SEM image of TCP on $Al_2O_3$ modified with a layer of TMSC (III)

### *8.2.2 DCP and TCP powders*

*DCP (crystallized from $PhNO_2$)*:

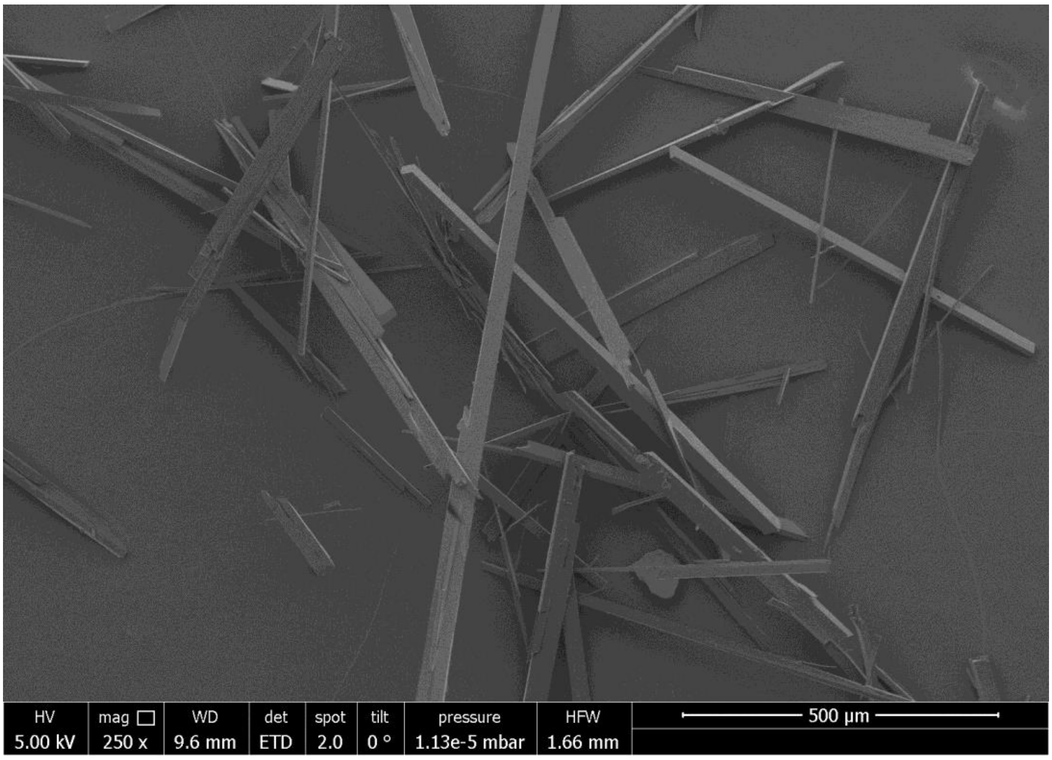


**Figure S49:** SEM image of DCP crystals (I)

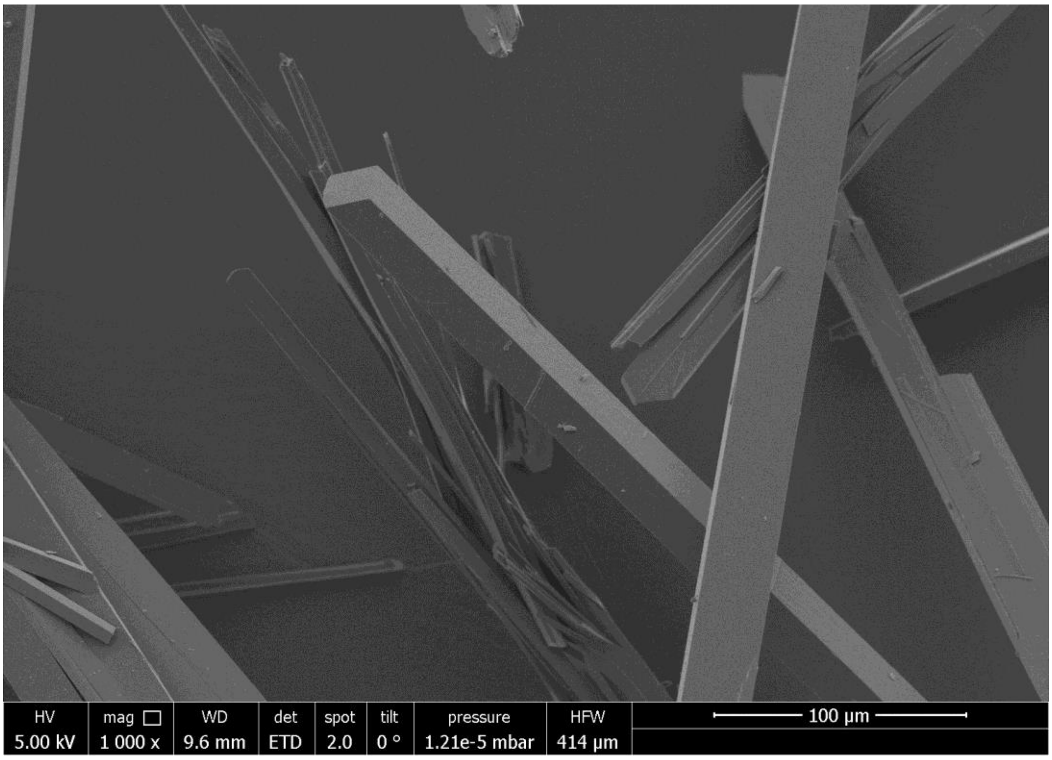


**Figure S50:** SEM image of DCP crystals (II)

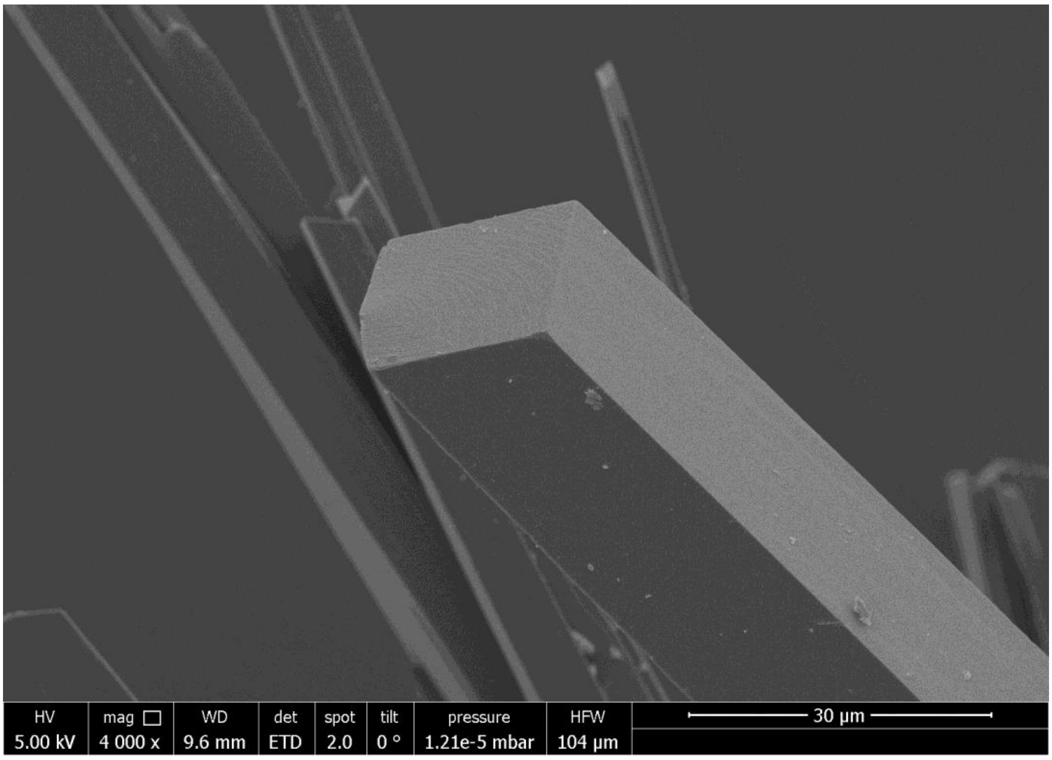


**Figure S51:** SEM image of DCP crystals (III)

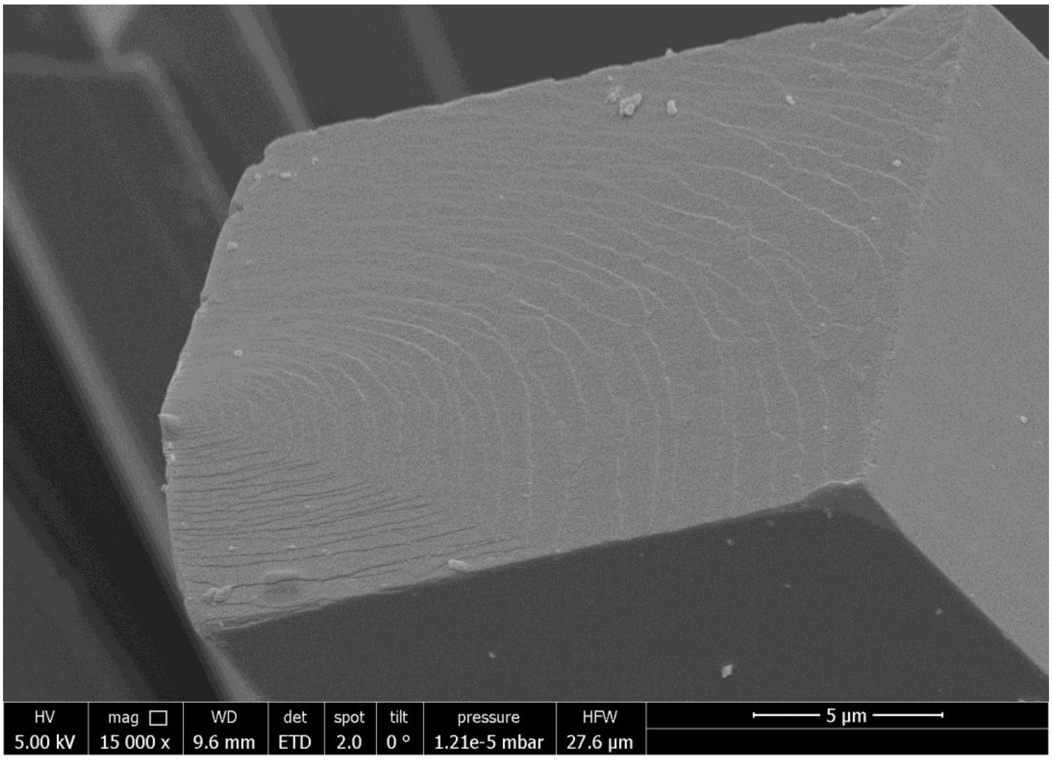


**Figure S52:** SEM image of DCP crystals (IV)

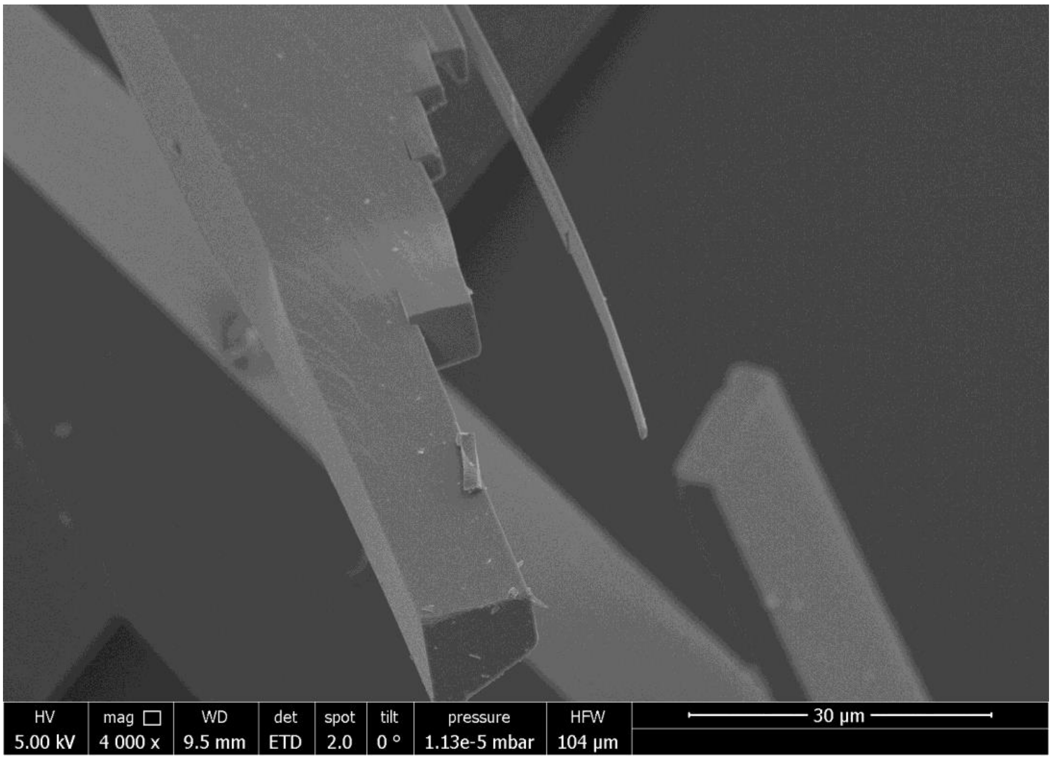


**Figure S53:** SEM image of DCP crystals (V)

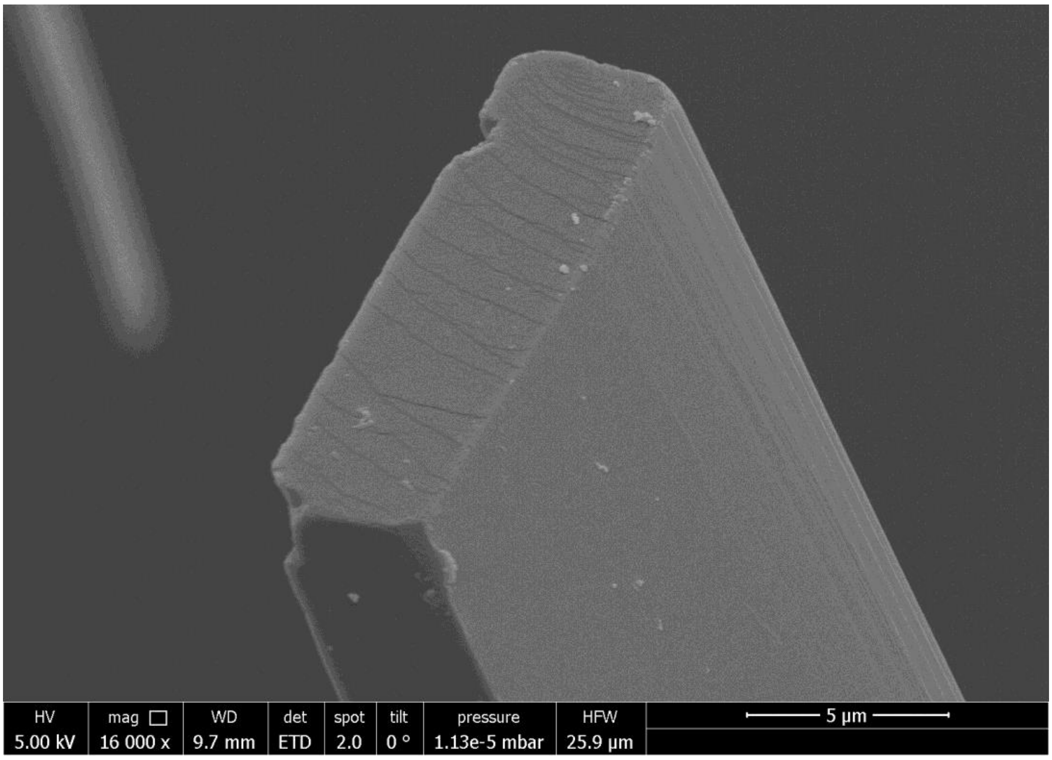


**Figure S54:** SEM image of DCP crystals (VI)

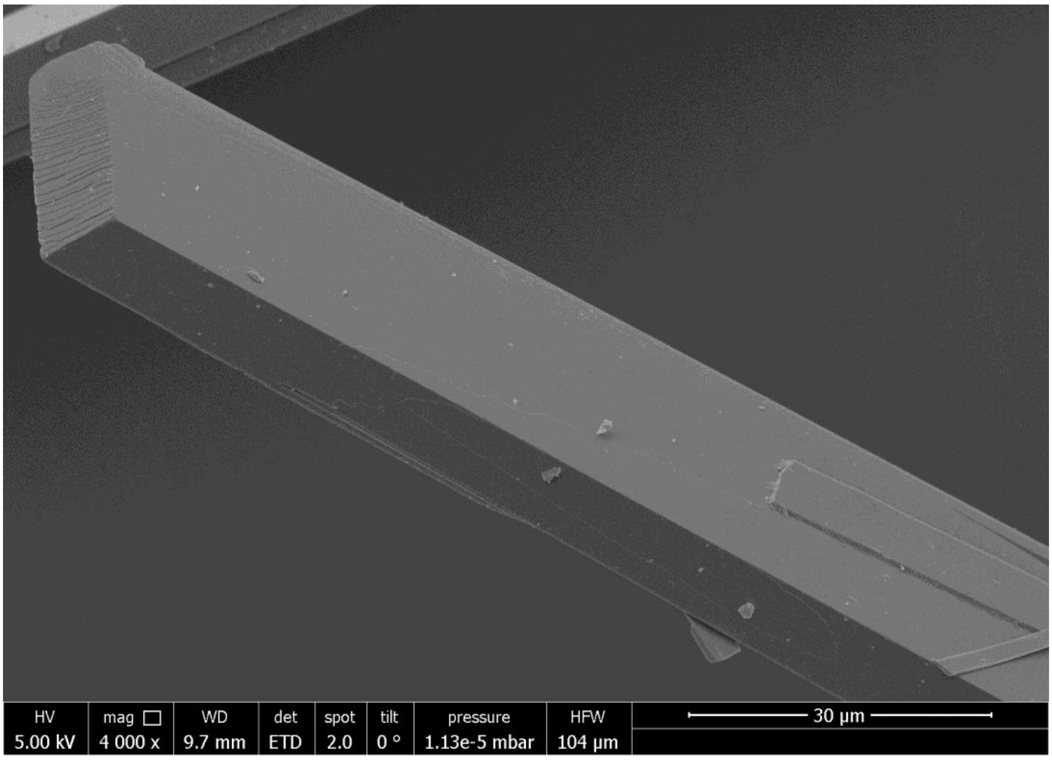


**Figure S55:** SEM image of DCP crystals (VII)

*TCP(˙PhNO$_2$) (crystallized from PhNO$_2$)*:

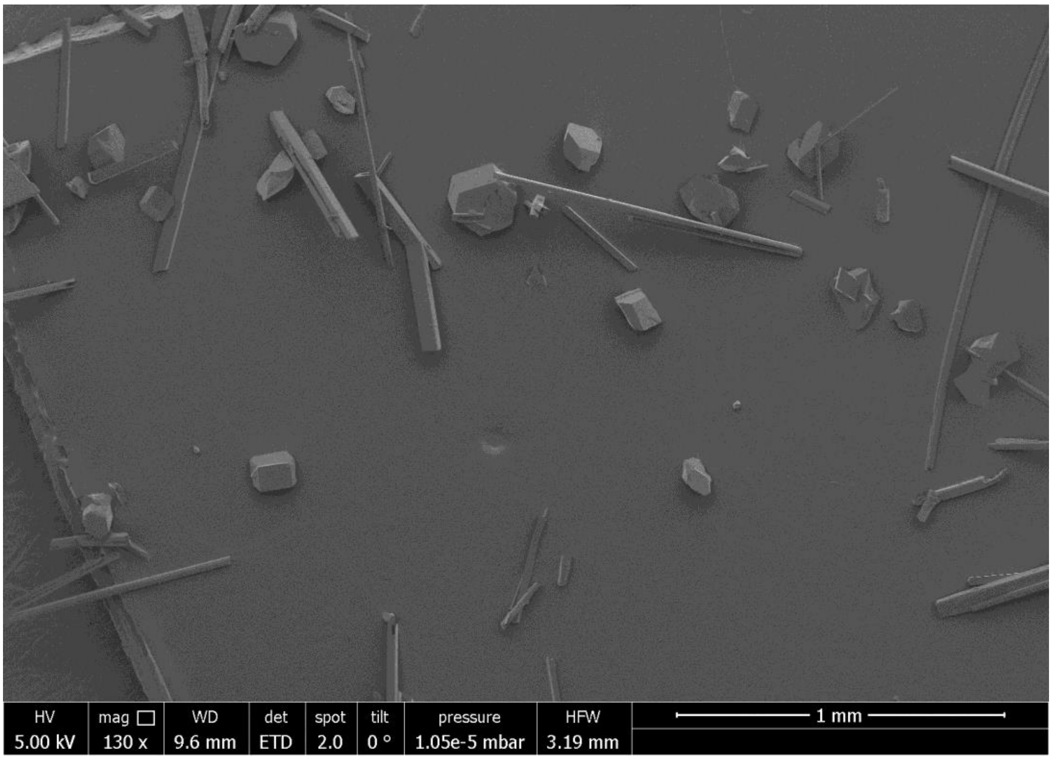


**Figure S56:** SEM image of TCP(˙PhNO$_2$) crystals (I)

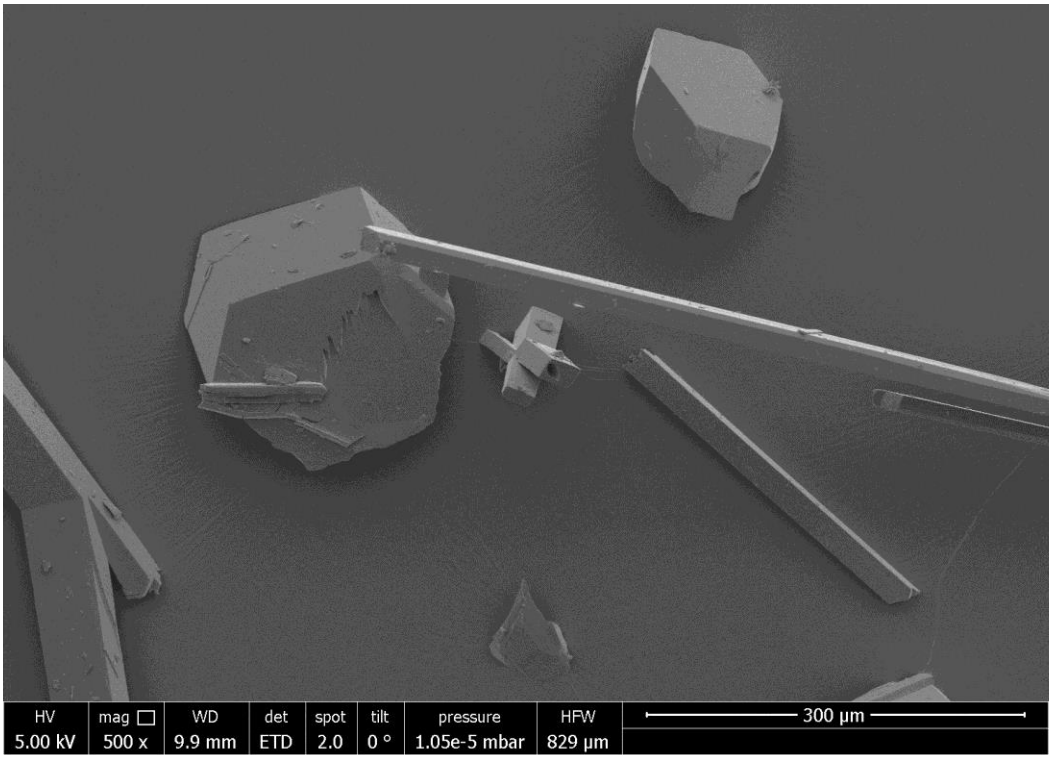


**Figure S57:** SEM image of TCP(·PhNO$_2$) crystals (II)

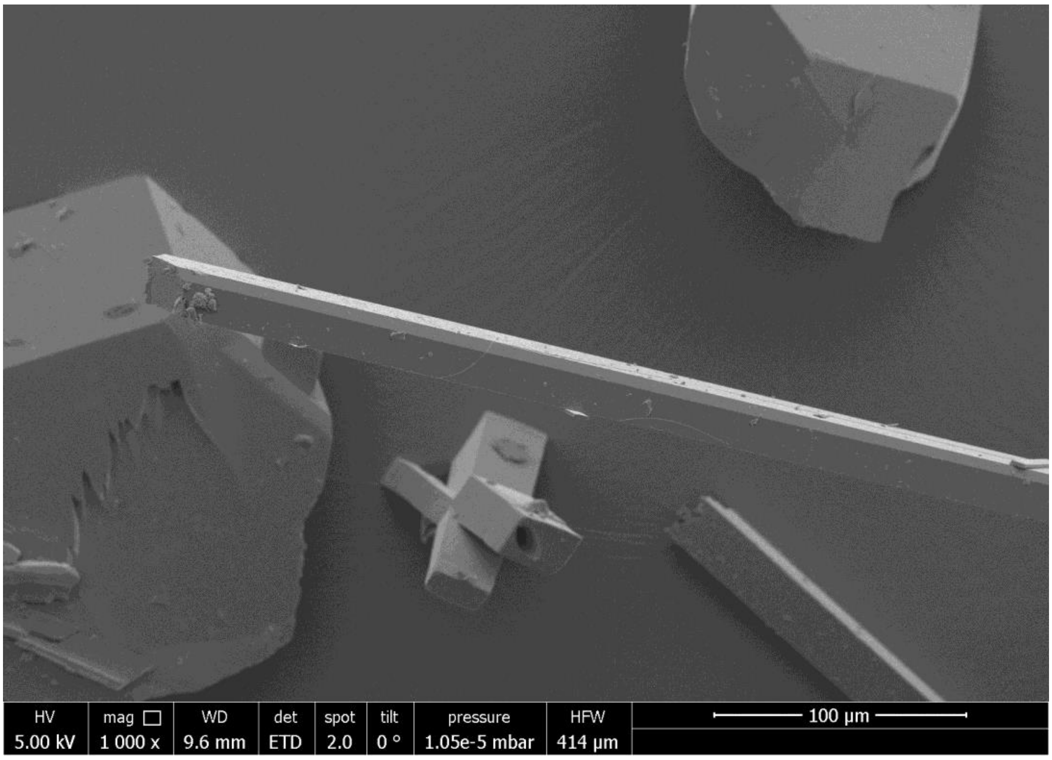


**Figure S58:** SEM image of TCP(·PhNO$_2$) crystals (III)

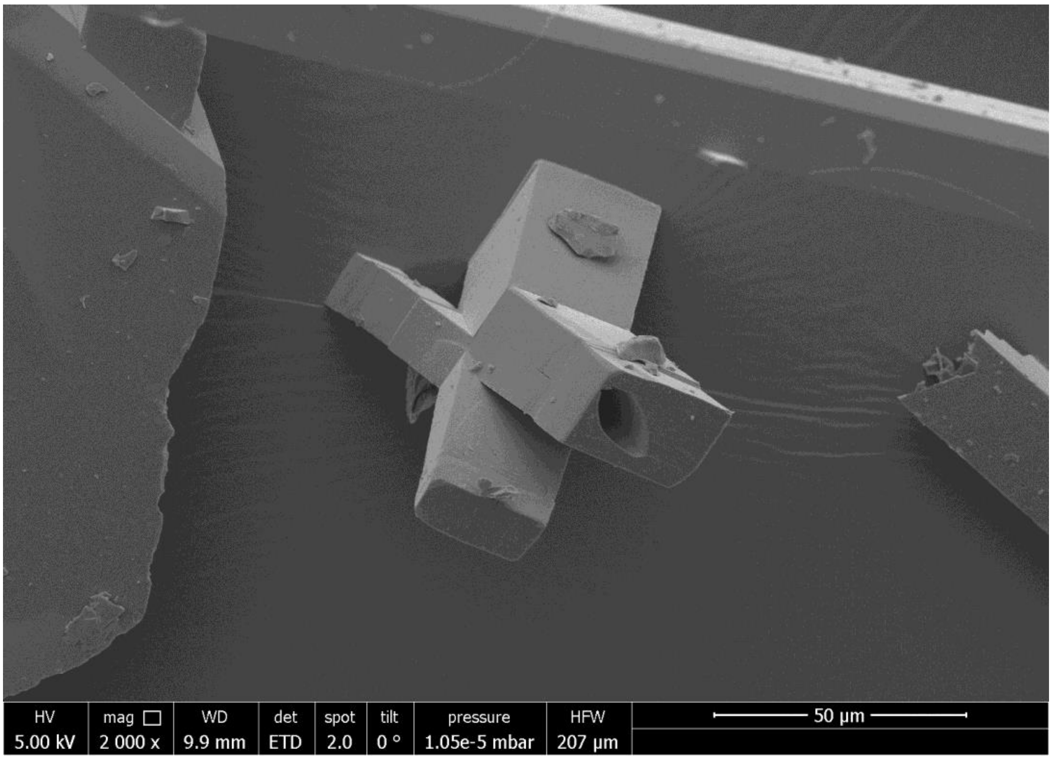


**Figure S59:** SEM image of TCP(·$PhNO_2$) crystals (IV)

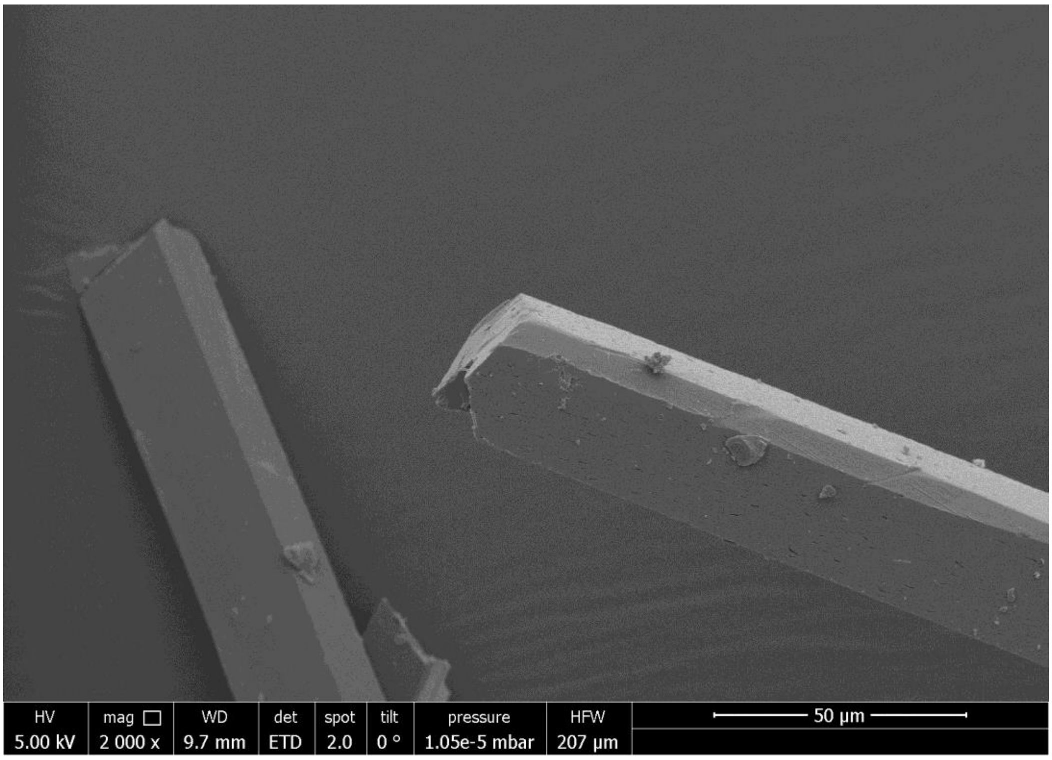


**Figure S60:** SEM image of TCP(·$PhNO_2$) crystals (V)

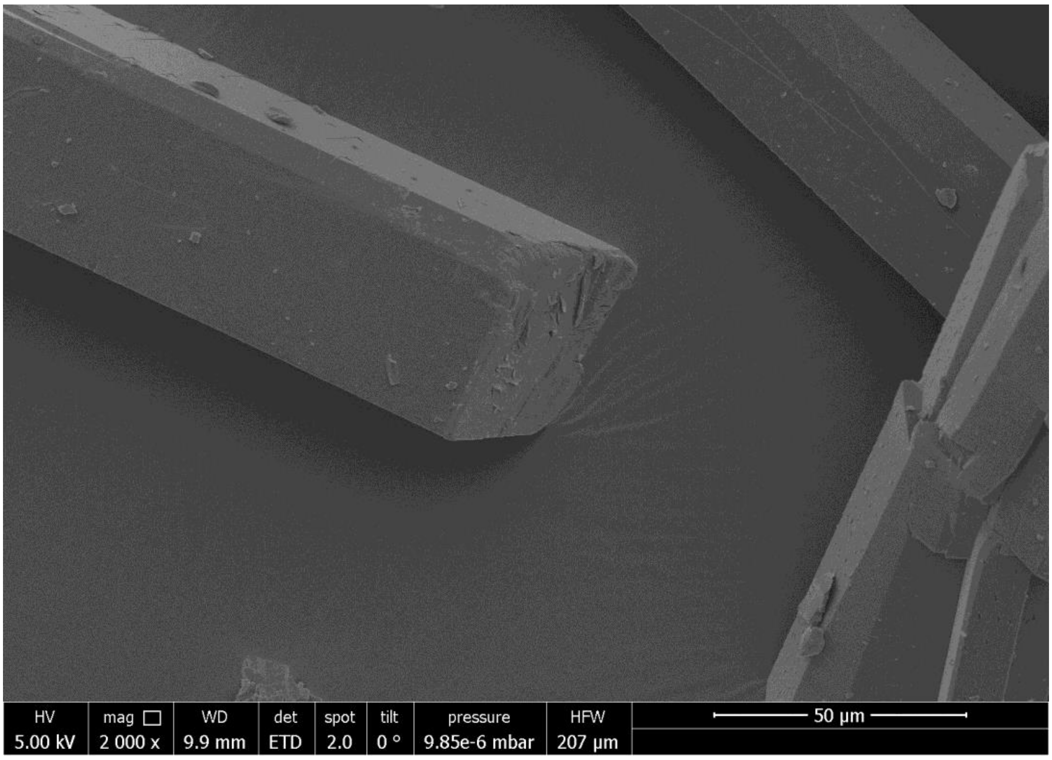


**Figure S61:** SEM image of TCP(·PhNO$_2$) crystals (VI)

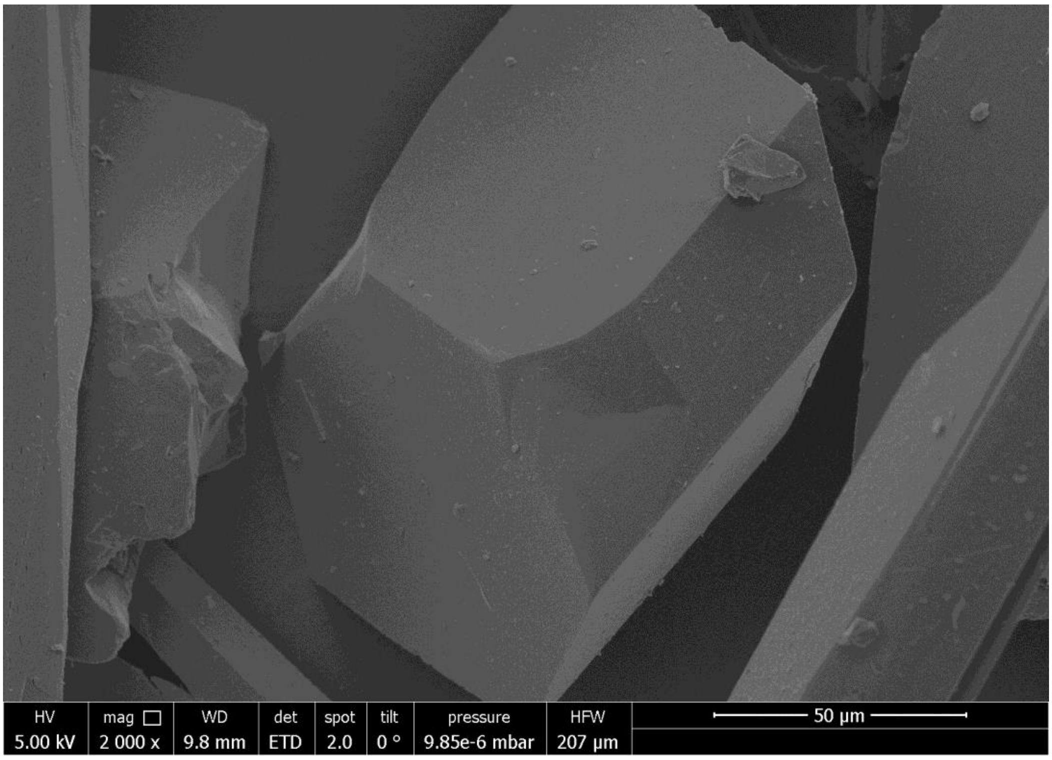


**Figure S62:** SEM image of TCP(·PhNO$_2$) crystals (VII)

*TCP (sublimated)*:

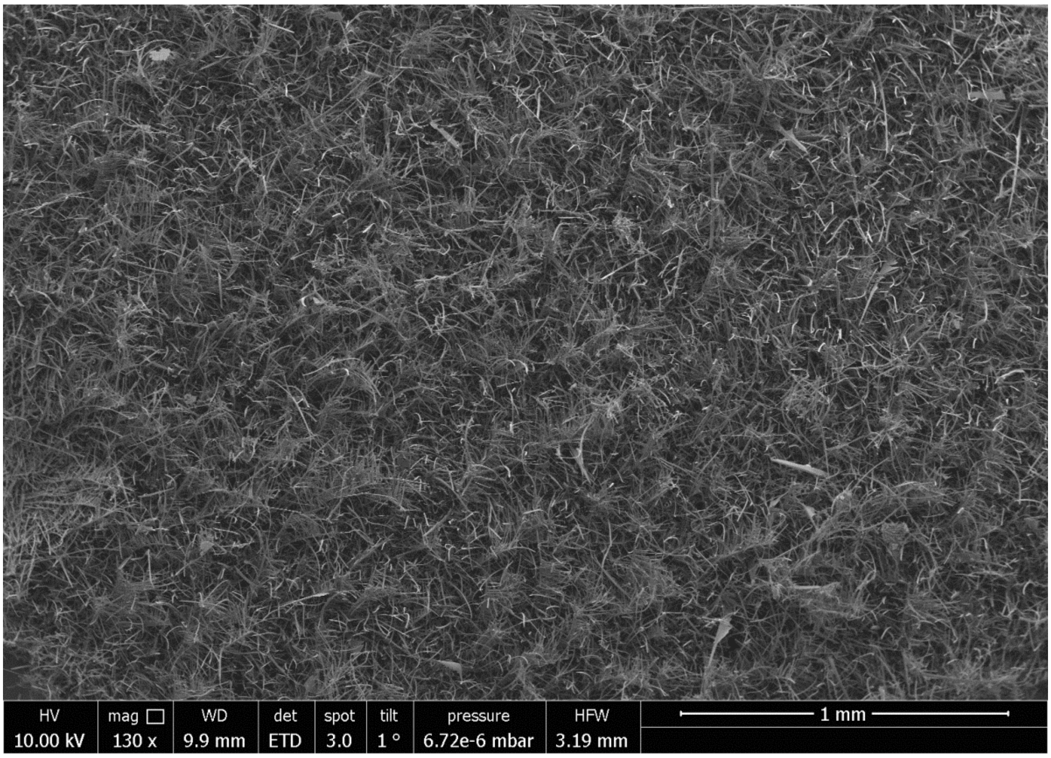


**Figure S63:** SEM image of sublimated TCP (I)

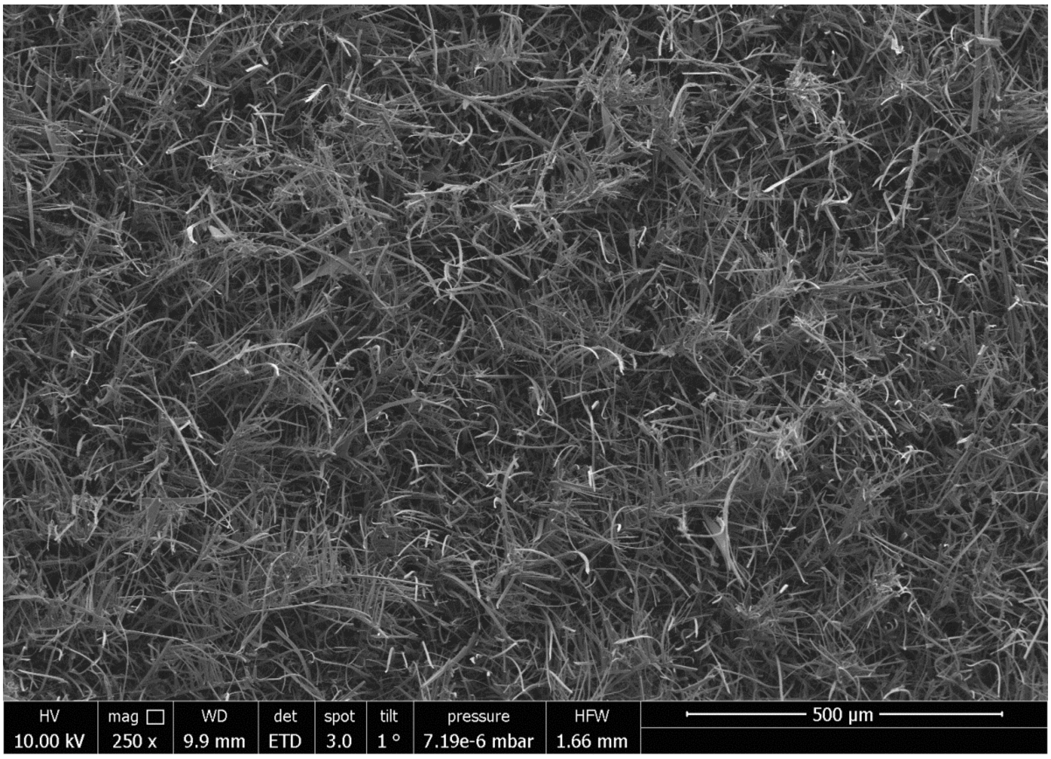


**Figure S64:** SEM image of sublimated TCP (II)

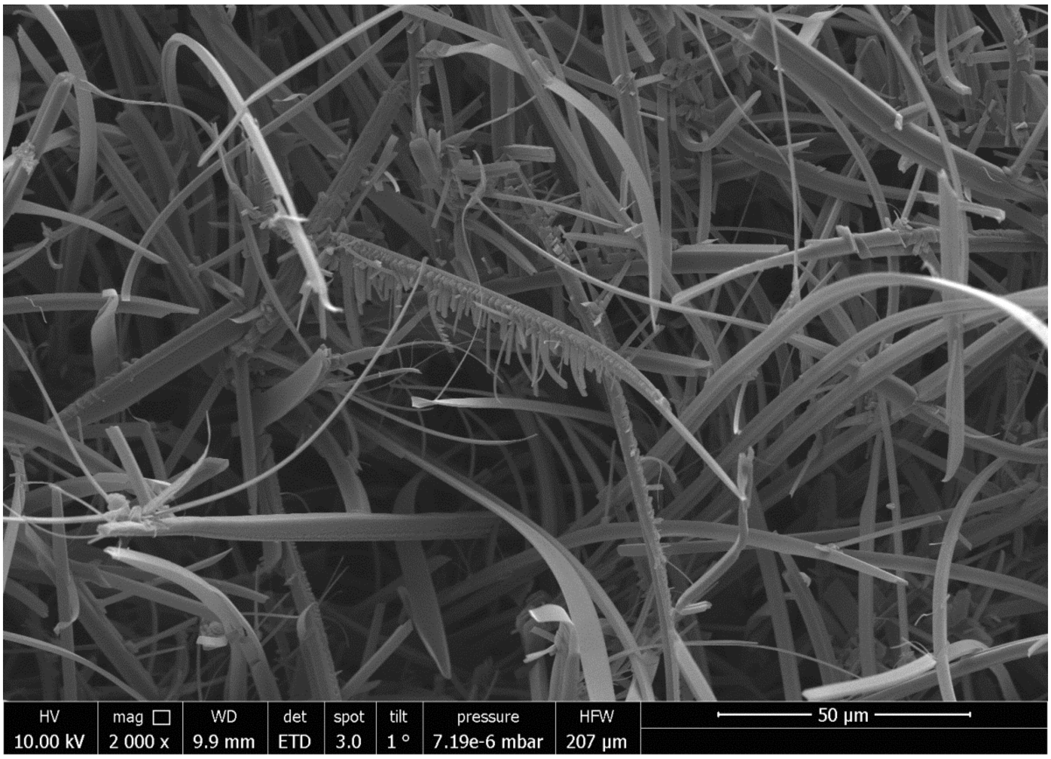


**Figure S65:** SEM image of sublimated TCP (III)

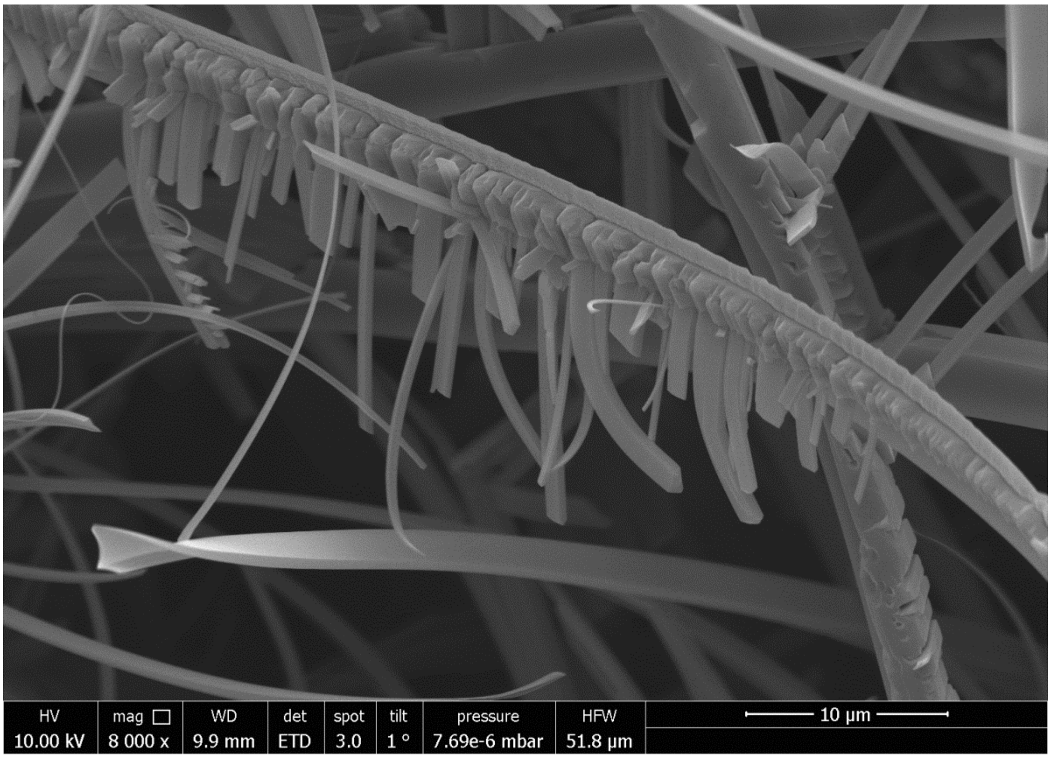


**Figure S66:** SEM image of sublimated TCP (IV)

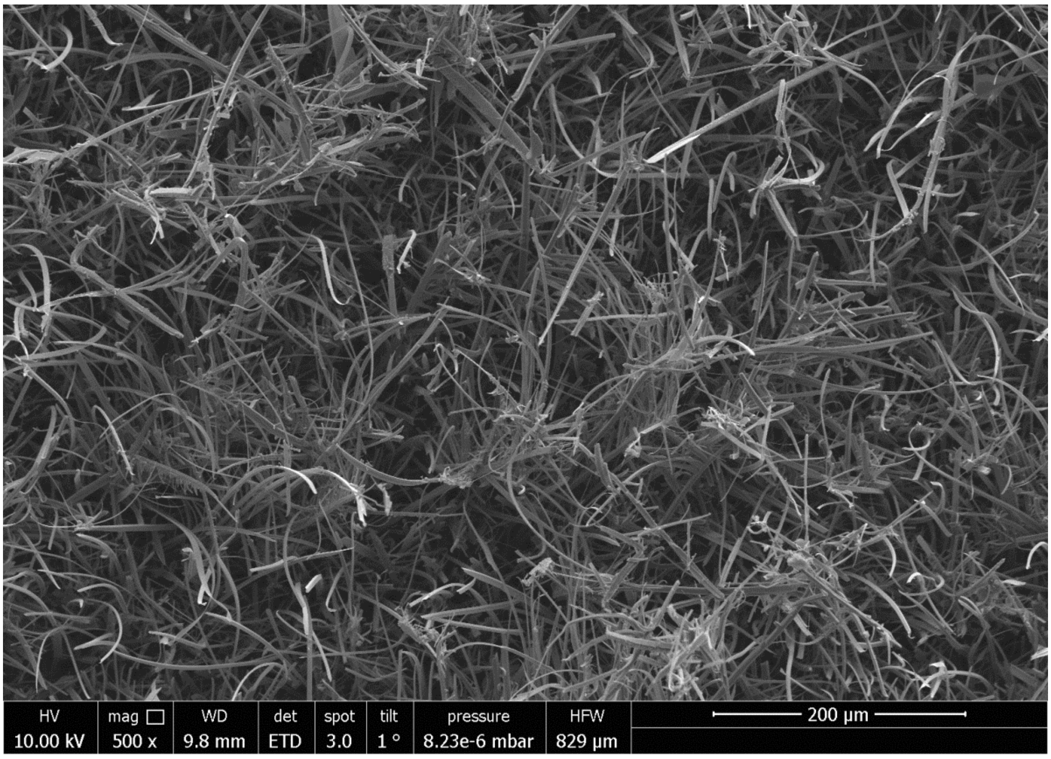


**Figure S67:** SEM image of sublimated TCP (V)

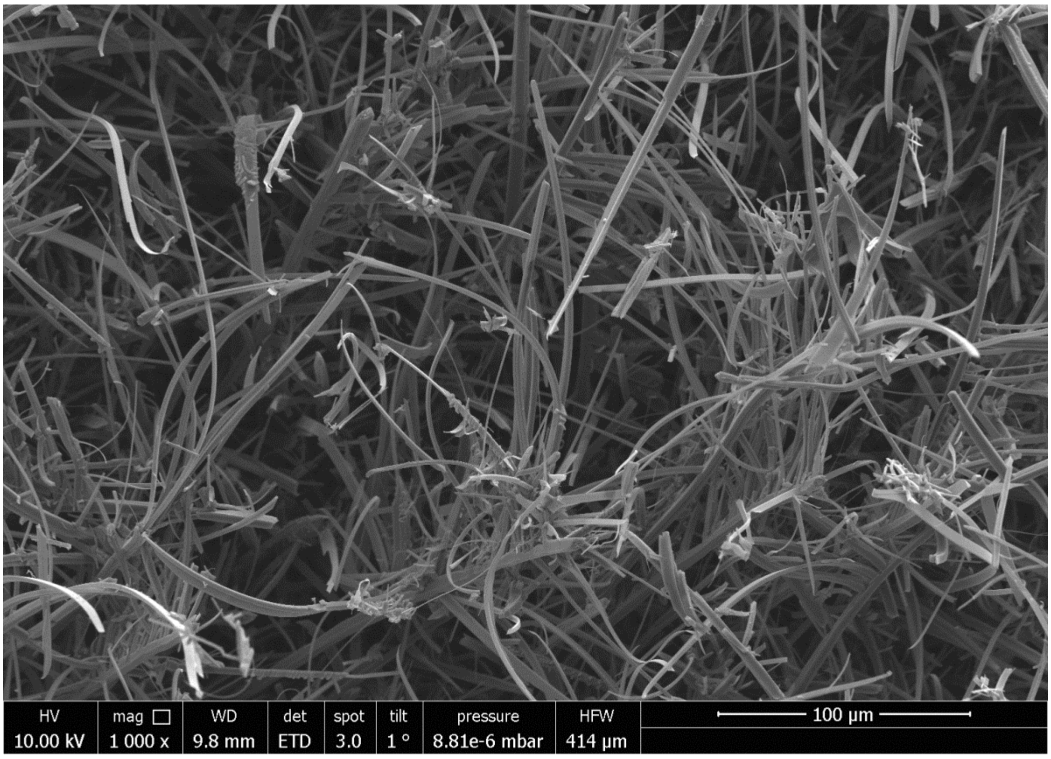


**Figure S68:** SEM image of sublimated TCP (VI)

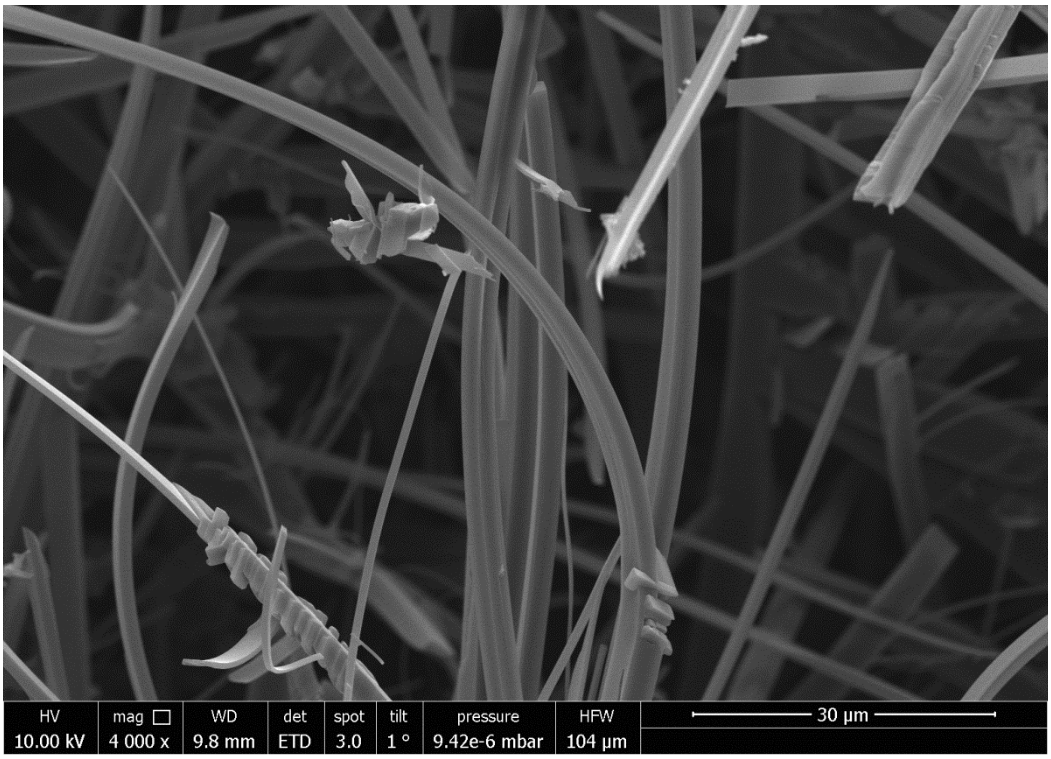


**Figure S69:** SEM image of sublimated TCP (VII)

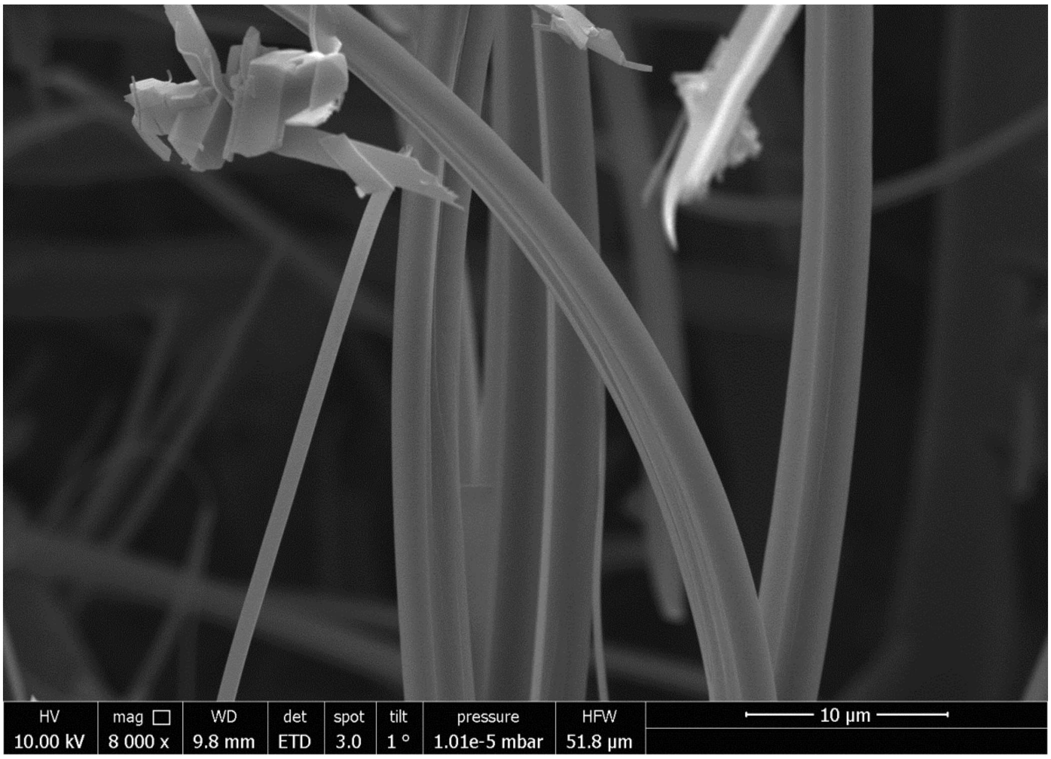


**Figure S70:** SEM image of sublimated TCP (VIII)

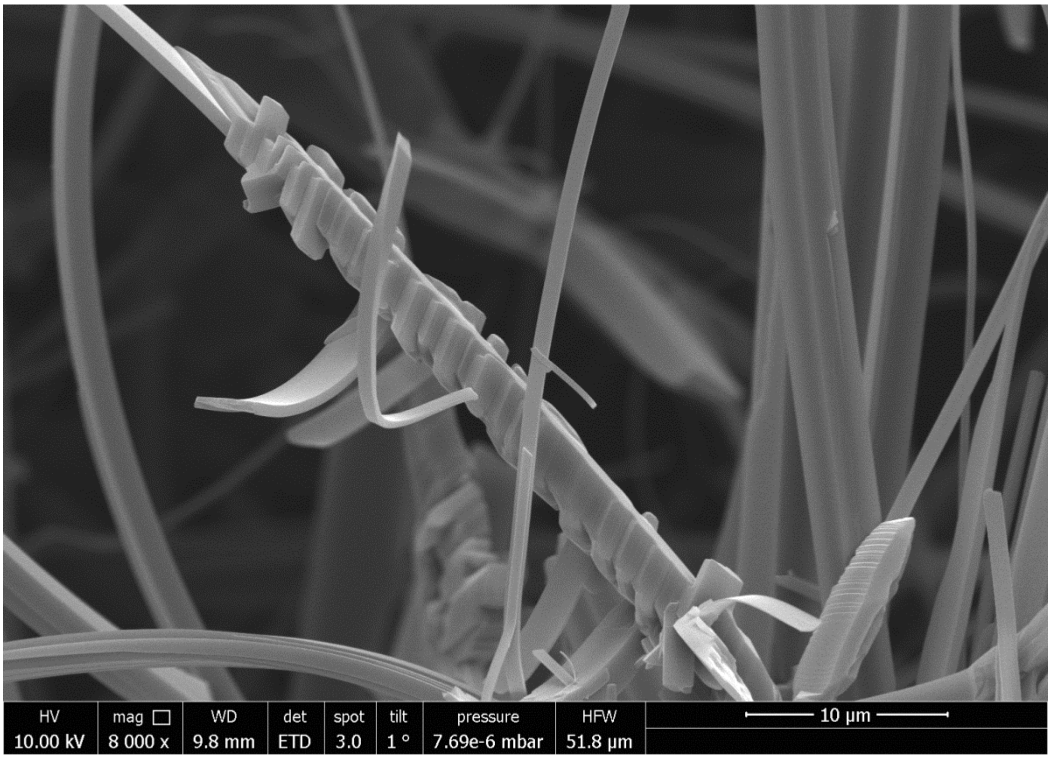


**Figure S71:** SEM image of sublimated TCP (IX)

# 9. Transistor fabrication and electrical characterization

Organic thin film transistors (OTFTs) were fabricated in a staggered bottom gate, top contact architecture according to the layer setup illustrated in Figure S72.

## 9.1. Fabrication and characterization of devices using $Al_2O_3$ with and without PNDPE capping layer as the gate dielectric

The gate electrode was processed under high vacuum condition on a pre-cleaned glass substrate. The aluminum gate electrode was deposited by thermal evaporation and deposition of a 60 nm thick aluminum layer through a shadow mask at a rate of 1 nm $s^{-1}$, followed by anodization of the aluminum to create 28 nm thick $Al_2O_3$.[S11] For the devices with an additional PNDPE capping layer, 40 nm thick PNDPE was spin coated atop of the $Al_2O_3$. The layer thicknesses of PNDPE films were determined by profilometer measurements (VEECO Dektak 150).
Thermal evaporation (DCP: ~140°C, TCP: ~230°C) and deposition of a 50 nm thick DCP or TCP layer was carried out using a high vacuum system (deposition rate: ~0.1 Å$s^{-1}$ (DCP), ~0.2-0.3 Å$s^{-1}$ (TCP); substrate temperatures: rt).
Source and drain electrodes were deposited by thermal evaporation and deposition of gold through a shadow mask (tungsten wires fixed over a gap) in order to form 50 nm thick contacts. Different channel widths and lengths were tested for all fabricated OTFTs (4 mm (W) / 50 µm (L); 3 mm / 35 µm; 2 mm / 25 µm; 1 mm / 10 µm) (see also Figure S75, Table S1).

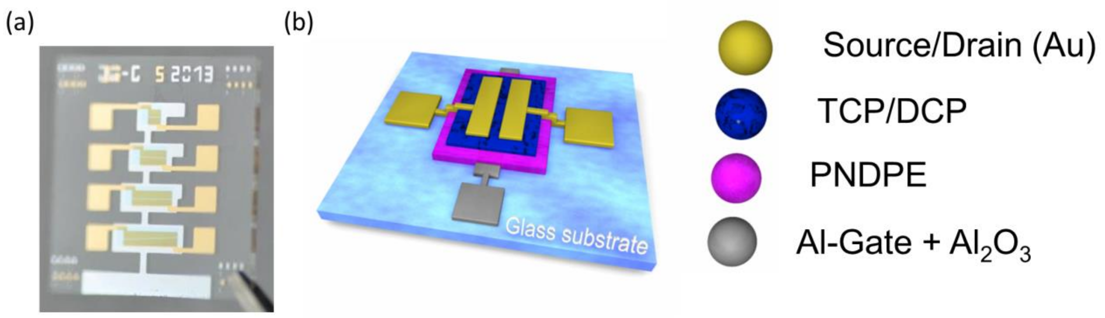


**Figure S72:** (a) Photograph showing four TCP based OTFTs on a glass substrate.
(b) Schematic of the fabricated OTFTs, showing the glass substrate (light blue), aluminum gate electrode + $Al_2O_3$ (dark grey), PNDPE (poly((±)endo,exo-bicyclo[2.2.1]hept-5-ene-2,3-dicarboxylic acid, diphenylester) (violet), organic semiconductor (TCP/DCP) (dark blue), and source- and drain electrodes (Au) (yellow).

Electrical measurements of OTFTs were carried out in a glove box under argon and outside the glovebox under ambient condition, both with exclusion of light, using a parameter analyzer from MB-Technologies. The $I_D(V_{GS})$ and $I_G(V_{GS})$, are plotted in semi-logarithmic representation; the threshold voltage $V_{Thr}$ and the semiconductor charge carrier mobility $\mu$ were extracted from the plot of the square root of the drain current as a function of the gate bias. The mobility $\mu$ was obtained from the slope of a linear fit through the data in this plot, the threshold voltage $V_{Thr}$ corresponds to the extrapolation of the line to zero current. The subthreshold swing S is the inverse of the subthreshold slope extracted as the maximum slope of the (quasi)linear part of the subthreshold current.

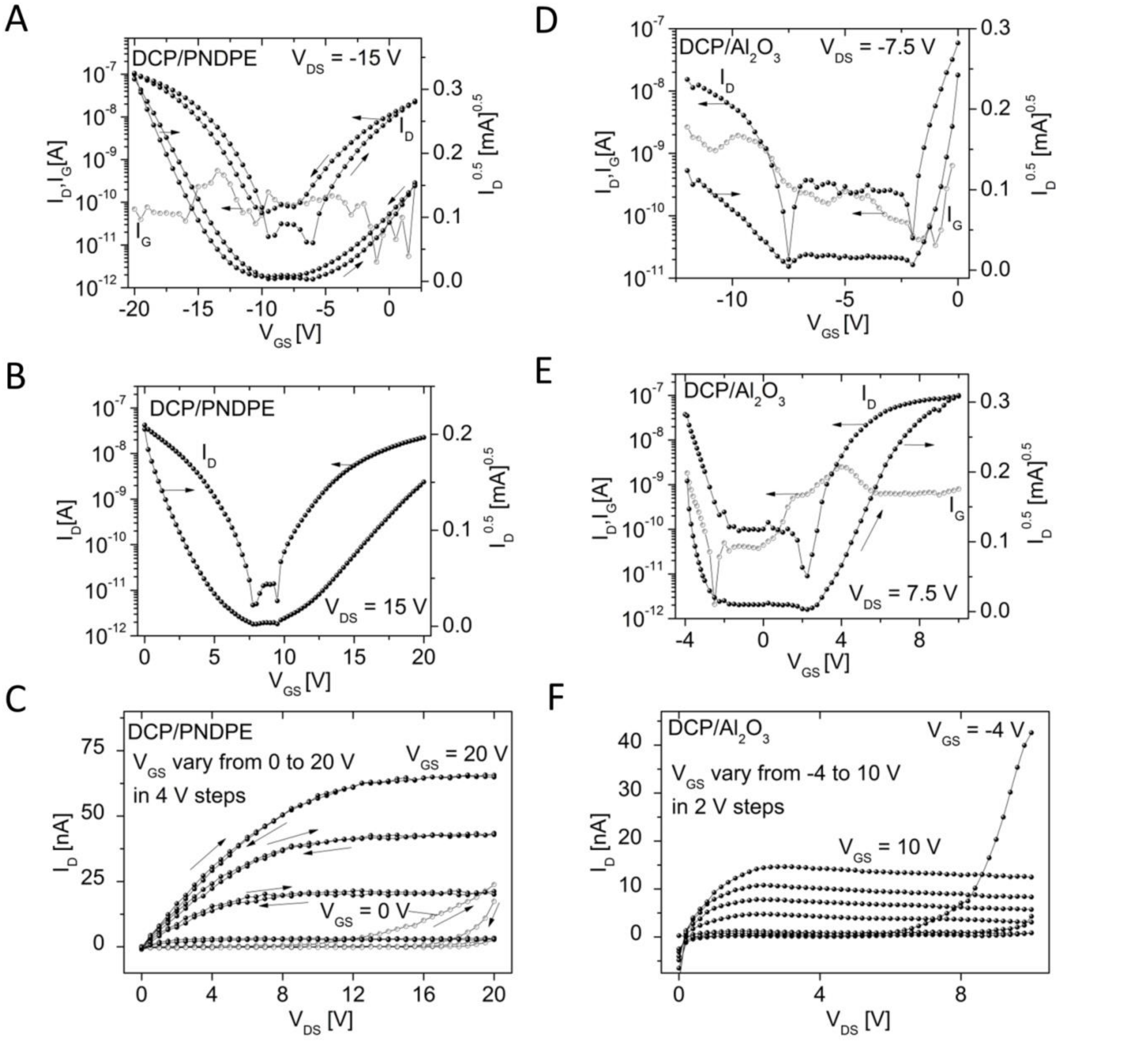


**Figure S73:** Transfer- (A,B,D,E) and output-characteristics (C,F) of DCP based OTFTs under argon with (A,B,C) and without (D,E,F) an additional PNDPE capping layer on the $Al_2O_3$ gate dielectric. The gate leakage current characteristics $I_G\,(V_{GS})$ are plotted in grey in (A), (D), and (E).

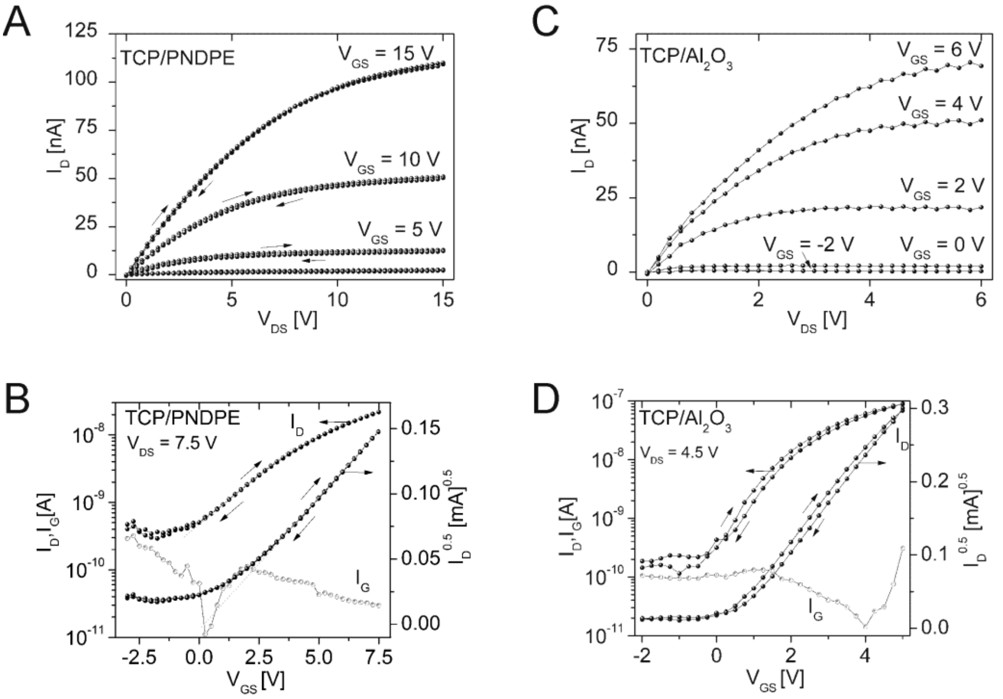


**Figure S74:** Output- (A,C) and transfer-characteristics (B,D) of TCP based OTFTs with (A,B) or without (C,D) an additional PNDPE capping layer on the $Al_2O_3$ gate dielectric. The gate leakage current characteristics $I_G$ ($V_{GS}$) are plotted in grey in (B) and (D).

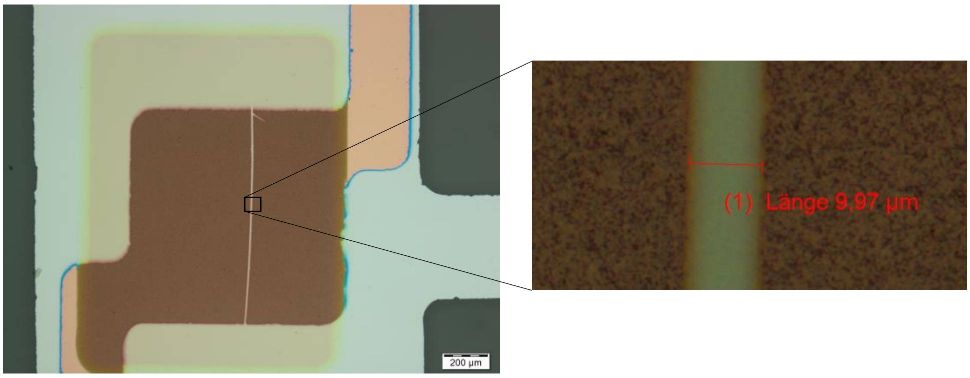


**Figure S75:** Microscope image of a TCP based OTFT with a bilayer gate dielectric ($Al_2O_3$ + cellulose) and a channel length of 10 µm.

**Table S1.** Mean channel lengths of all fabricated OTFTs (with standard deviations; measured under the microscope) depending on the diameter of the shadow mask.

| Diameter of shadow mask (tungsten wire) | Channel lengths [µm] of fabricated devices |
|---|---|
| 50 | 51,2 ± 1,3 |
| 35 | 36,6 ± 1,0 |
| 25 | 25,3 ± 0,9 |
| 10 | 11,2 ± 1,1 |

### 9.2. Overview of tested dielectrics

A proper choice of the gate dielectric is essential to achieve good transistor characteristics. In Table S2 all tested dielectric configurations of DCP and TCP based transistors are listed.

TCP and DCP films were investigated on low ($Al_2O_3$ + fluoroalkyl phosphonic acid SAM, $Al_2O_3$ + TMSC) and high surface energy materials ($Al_2O_3$, $Al_2O_3$ + PNDPE, $Al_2O_3$ + Cellulose) with low and high polar fraction and surface roughness below 3 nm. The deposition conditions for TCP and DCP were similar for all dielectric configurations (see above for the detailed conditions).

For TCP based OTFTs n-type operation is observed for all different gate dielectric configurations. The best results under ambient and inert conditions were obtained on high surface energy materials ($Al_2O_3$, $Al_2O_3$ + PNDPE) (Table S3). Hole transport (p-type operation) is excluded for TCP, since the injection barrier for holes is too high in the case of gold electrodes. DCP based devices show p- and n-type operation on low ($Al_2O_3$ + TMSC) and high energy surface materials ($Al_2O_3$, $Al_2O_3$ + PNDPE) (Table S3). On cellulose no transistor performance is observed. DCP on fPA-SAM modified anodized aluminum show only p-type operation.

In summary, for TCP films the best transistor results were obtained by using high surface energy materials. For DCP based devices reasonable result were observed on high and low surface energy materials. However, there is still a lot of room for improvements: Careful optimization of the processing of the semiconductor materials (purification by thermal gradient sublimation, improved deposition by identifying the optimum deposition parameters, i.e. vacuum deposition rate and substrate temperature) may lead to an improved growth of the semiconductor crystals and may improve the transistor performance significantly.

**Table S2.** Overview of all tested dielectric configurations and the obtained results

| | | Dielectric configurations [a)] | | | | |
|---|---|---|---|---|---|---|
| | | $Al_2O_3$ | $Al_2O_3$ + PNDPE | $Al_2O_3$ + fPA | $Al_2O_3$ + Cellulose | $Al_2O_3$ + TMSC |
| TCP | n-type operation | Yes | Yes | Yes* | Yes* | Yes* |
| | p-type operation | - | - | - | - | - |
| DCP | n-type operation | Yes | Yes | - | - | Yes** |
| | p-type operation | Yes | Yes | Yes** | - | Yes** |

[a)] Gate dielectric configurations: 28 nm $Al_2O_3$ with or without additional layers. The additional layers are (1) ~40 nm PNDPE (poly((±)endo,exo-bicyclo[2.2.1]hept-5-ene-2,3-dicarboxylic acid, diphenylester), (2) fPA (fluoroalkyl phosphonic acid self-assembled monolayer), (3) ~15 nm Cellulose, (4) ~50 nm TMSC (trimethylsilylcellulose).
*The charge carrier mobility of these devices is below $1 \times 10^{-5}$ cm$^2$ V$^{-1}$s$^{-1}$. **The charge carrier mobility of these devices is in the range of $1 \times 10^{-4}$ to $5 \times 10^{-4}$ cm$^2$ V$^{-1}$s$^{-1}$.
Preparation of the additional layers: (1) PNDPE: see above. (2) fPA: The SAM was applied by immersing the substrate for 3 hours in a 2 mM solution of 1H,1H,2H,2H-perfluorooctane phosphonic acid in 2-propanol, allowing an organic monolayer to self-assemble on the oxidized surface. Prior to immersing the substrate, a brief oxygen plasma treatment was performed in order to activate the surface for the SAM-treatment. The preparation of cellulose (3) and TMSC (4) thin-films is described in Ref. S12.

**Table S3.** Transistor parameters for DCP and TCP based OTFTs under inert and ambient conditions using different gate dielectrics.

| | Dielectric [a)] | | *ambient* | | | *inert* | | |
|---|---|---|---|---|---|---|---|---|
| | | | $V_{Thr}$ [V] [b)] | $S$ [V dec.$^{-1}$] [c)] | $\mu_{sat}$ [cm$^2$ V$^{-1}$ s$^{-1}$] [d)] | $V_{Thr}$ [V] | $S$ [V dec.$^{-1}$] [c)] | $\mu_{sat}$ [cm$^2$ V$^{-1}$ s$^{-1}$] [d)] |
| DCP | $Al_2O_3$ | n-type | n/a | n/a | n/a | 3.4 (±0.4) | 0.6 (±0.2) | $3.9 \times 10^{-4}$ (±$1.7 \times 10^{-4}$) |
| | | p-type | n/a | n/a | n/a | -7.8 (±0.6) | 0.5 (±0.1) | $2.0 \times 10^{-4}$ (±$0.9 \times 10^{-4}$) |
| | $Al_2O_3$ + PNDPE | n-type | 10.5 (±1.5) | 1.0 (±0.3) | $1.9 \times 10^{-4}$ (±$1.3 \times 10^{-4}$) | 8.7 (±1.4) | 1.7 (±0.4) | $1.8 \times 10^{-4}$ (±$1.4 \times 10^{-4}$) |
| | | p-type | -12.3 (±0.4) | 0.9 (±0.2) | $2.9 \times 10^{-4}$ (±$1.9 \times 10^{-4}$) | -11.3 (±0.4) | 1.1 (±0.3) | $5.3 \times 10^{-4}$ (±$1.8 \times 10^{-4}$) |
| TCP | $Al_2O_3$ | n-type | 0.45 (±0.4) | 2.1 (±0.3) | $1.9 \times 10^{-4}$ (±$0.5 \times 10^{-4}$) | 0.55 (±0.35) | 0.9 (±0.2) | $2.9 \times 10^{-4}$ (±$1.4 \times 10^{-4}$) |
| | $Al_2O_3$ + PNDPE | n-type | -0.35 (±1.3) | 5.5 (±0.4) | $1.8 \times 10^{-4}$ (±$0.4 \times 10^{-4}$) | -0.4 (±2.0) | 5.8 (±0.4) | $2.2 \times 10^{-4}$ (±$0.2 \times 10^{-4}$) |

a) gate dielectric configurations: 28 nm anodized $Al_2O_3$ (capacitance per cm$^2$ $C$ = 285 ±10 nF cm$^{-2}$) or 40 nm PNDPE on anodized Al (28 nm $Al_2O_3$; $C$ = 46 ± 5 nF cm$^{-2}$)); b) threshold voltage; c) subthreshold swing; d) charge carrier mobility in the saturation regime; b-d) values are calculated mean values with their standard deviations extracted of device parameters from four OTFTs.

# 10. References


[S1] F. Glöcklhofer, M. Lunzer, B. Stöger, J. Fröhlich, *Chem. Eur. J.*, 2016, **22**, 5173-5180.
[S2] Bruker, *APEX2*, *SAINT-Plus* and *SADABS* 2013, Bruker AXS Inc., Madison, Wisconsin, USA.
[S3] L. Palatinus, G. Chapuis, *J. Appl. Crystallogr.*, 2007, **40**, 786-790.
[S4] V. Petříček, M. Dušek, L. Palatinus, *Z. Kristallogr. - Cryst. Mater.*, 2014, **229**, 345-352.
[S5] A. L. Spek, *Acta Crystallogr. Sect. D*, 2009, **65**, 148-155.
[S6] a) A. Le Bail, H. Duroy, J. L. Fourquet, *Mater. Res. Bull.*, 1988, **23**, 447-452; b) G. S. Pawley, *J. Appl. Crystallogr.*, 1981, **14**, 357-361.
[S7] H. Sun, *J. Phys. Chem. B.*, 1998, **102**, 7338-7364.
[S8] G. E. Engel, S. Wilke, O. König, K. D. M. Harris, F. J. J. Leusen, *J. Appl. Crystallogr.*, 1999, **32**, 1169-1179.
[S9] S. K. Wolff, D. J. Grimwood, J. J. McKinnon, M. J. Turner, D. Jayatilaka, M. A. Spackman, *CrystalExplorer (Version 3.1)*, University of Western Australia, 2012.
[S10] a) M. A. Spackman, D. Jayatilaka, *CrystEngComm*, 2009, **11**, 19-32. b) M. A. Spackman, J. J. McKinnon, *CrystEngComm*, 2002, **4**, 378-392. c) J. J. McKinnon, D. Jayatilaka, M. A. Spackman, *Chem. Commun.*, 2007, 3814-3816.
[S11] A. Petritz, A. Wolfberger, A. Fian, T. Griesser, M. Irimia-Vladu, B. Stadlober, *Adv. Mat.*, 2015, **27**, 7645-7656.
[S12] A. Petritz, A. Wolfberger, A. Fian, M. Irimia-Vladu, A. Haase, H. Gold, T. Rothländer, T. Griesser, B. Stadlober, *Appl. Phys. Lett.*, 2013, **103**, 153303.
